# Rational incorporation of any unnatural amino acid into proteins by machine learning on existing experimental proofs

**DOI:** 10.1016/j.csbj.2022.08.063

**Published:** 2022-09-05

**Authors:** Haoran Zhang, Zhetao Zheng, Liangzhen Dong, Ningning Shi, Yuelin Yang, Hongmin Chen, Yuxuan Shen, Qing Xia

**Affiliations:** State Key Laboratory of Natural and Biomimetic Drugs, Department of Chemical Biology, School of Pharmaceutical Sciences, Peking University, Beijing 100191, China

**Keywords:** Protein design, Unnatural amino acid incorporation, Genetic code expansion, Machine learning, Virtual screening

## Abstract

The unnatural amino acid (UAA) incorporation technique through genetic code expansion has been extensively used in protein engineering for the last two decades. Mutations into UAAs offer more dimensions to tune protein structures and functions. However, the huge library of optional UAAs and various circumstances of mutation sites on different proteins urge rational UAA incorporations guided by artificial intelligence. Here we collected existing experimental proofs of UAA-incorporated proteins in literature and established a database of known UAA substitution sites. By program designing and machine learning on the database, we showed that UAA incorporations into proteins are predictable by the observed evolutional, steric and physiochemical factors. Based on the predicted probability of successful UAA substitutions, we tested the model performance using literature-reported and freshly-designed experimental proofs, and demonstrated its potential in screening UAA-incorporated proteins. This work expands structure-based computational biology and virtual screening to UAA-incorporated proteins, and offers a useful tool to automate the rational design of proteins with any UAA.

## Graphical abstract

**Figure:**
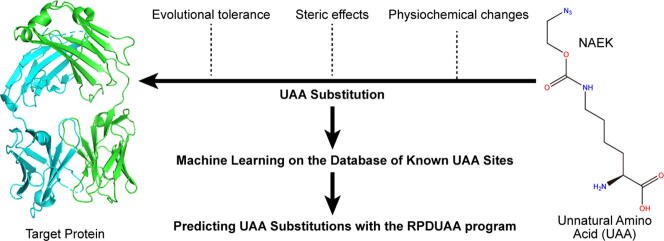

Site-specific incorporation of unnatural amino acids (UAAs) into proteins can be realized by nonsense codon suppression with the engineered tRNA and aminoacyl-tRNA synthetase (aaRS) pairs, a powerful technique developed by Schultz et al. in 2001 for the study of protein structures and functions [1]. In the last two decades, over 150 UAAs have been incorporated into various proteins in bacterial, yeast, and mammalian cells [2]. More recently, this technique has been expanded to transgenic animals including worms, fruit flies, zebrafish, and mice [3]. Since proteins are mostly composed of 20 canonical or natural amino acid (NAA) residues, the UAA substitution of a specific NAA residue, also termed as the NAA → UAA mutation, can render the protein new features or altered functions [4], such as photo-crosslinking [5], biorthogonal labelling [6], site-specific conjugation [7] or improved enzymatic activities [8]. The reported UAA-incorporated proteins could serve as a good database resource for investigating the rules of UAA substitutions.

Several factors are generally considered to influence the efficiency or outcome of UAA substitutions [9], which can be classified into three categories: the evolutional tolerance of the protein sequence, the steric effects of the protein structure, and the physiochemical changes accompanying the NAA → UAA mutation (Fig. 1a). Some conserved sites crucial for protein functions tend to be less tolerant for UAA substitutions [10]. Surface-exposed residues are preferred for UAA substitutions due to their little perturbation to protein structure and easy accessibility of subsequent labelling [11]. The substitutions are location-dependent and the circumstances vary among different residues and proteins. Besides, UAAs and NAAs may have physiochemical differences in aspects of polarity, hydrophobicity, and hydrogen bonds. UAAs with similar physiochemical properties to NAAs are more likely to make a successful substitution, in which previous researchers tend to acquiesce [12]. So, the UAA substitution is a multivariate process that remains challenging to predict [13]. A recent study has shown the ability of machine learning in predicting the site tolerability for one UAA [14]. For the general case of any UAA substituting any site on any protein, a reliable model or program integrating all relevant factors is still needed for instructive predictions or virtual screening of UAA-incorporated proteins.

**Fig. 1:**
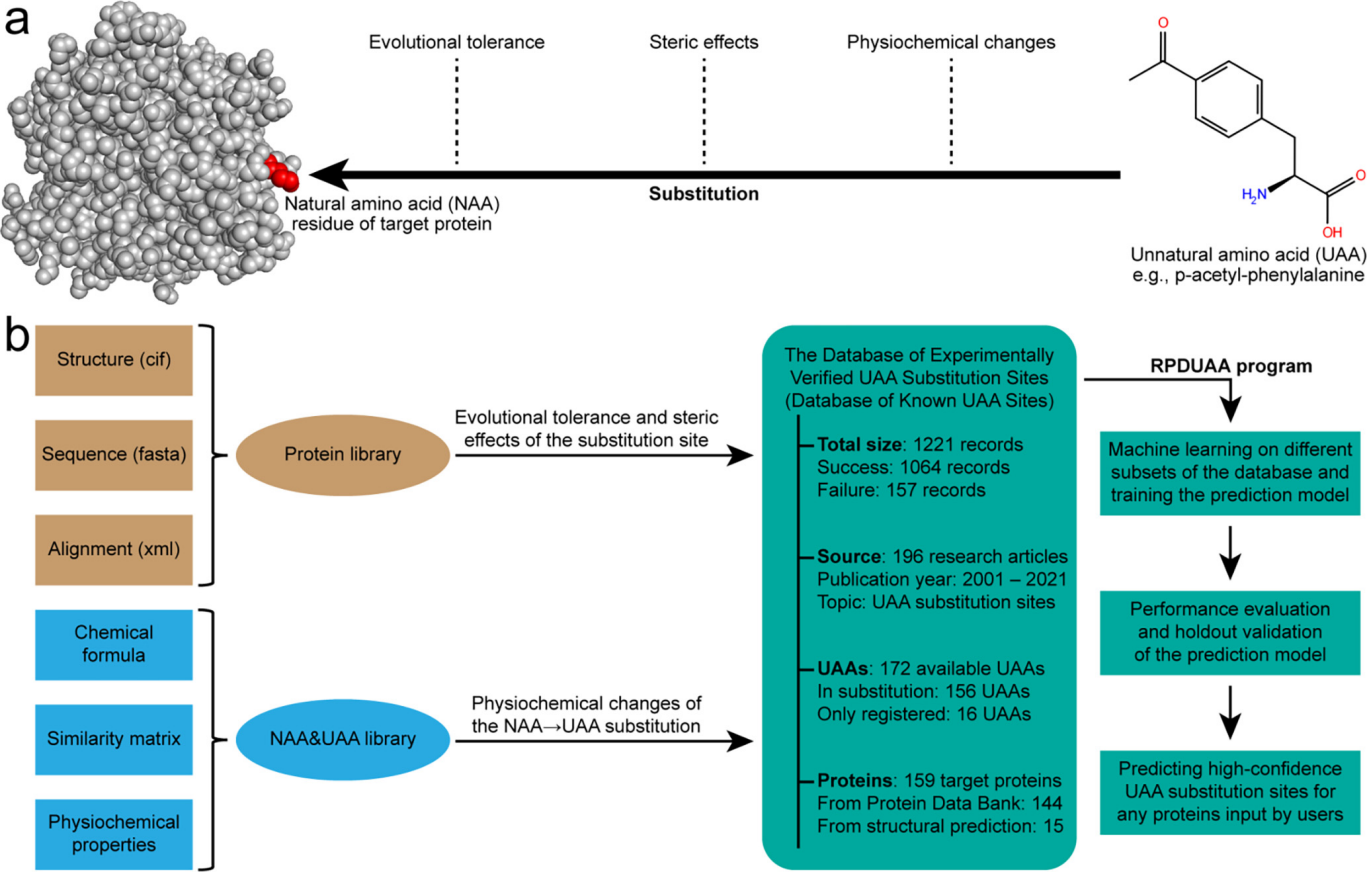
**Architecture of this study.** (a) Schematic illustration of a typical UAA substitution and the related influencing factors. (b) Flow chart of preparing the database and designing the RPDUAA program.

In this research, we systematically collected existing records of UAA-incorporated proteins from 196 articles published between 2001 and 2021, and created a database of experimentally verified UAA substitution sites (the database of known UAA sites, see Fig. 1b and Supplementary Data) for machine learning. The target proteins, NAAs and UAAs were registered in local libraries to compute evolutional tolerances, steric effects and physiochemical changes as observation factors. By designing a program called “Rational Protein Design with Unnatural Amino Acids” (the RPDUAA program, see Supplementary Guide) and performing logistic regression between the UAA substitution outcome (success or failure) and multiple observation factors, we tested and validated the performance of the prediction model. Finally, we designed 42 UAA-incorporated proteins and examined the strategy of probability-based UAA incorporations using RPDUAA.

## 1 Results

### 1.1 Preparation of the database and libraries

The UAA-incorporated proteins reported in previous researches can provide insight into the process of UAA substitutions, and we started by reorganizing them into a database. By searching in PubMed for articles that contain “unnatural/non-canonical amino acid incorporation/substitution/replacement” in the title or abstract, we found more than 500 entries. Further, 196 research articles which give definite information of the target protein, the substitution site, the substituted NAA, the substituting UAA, and the substitution outcome were considered. To improve the quality and creditability of the database, several inclusion and exclusion criteria were applied during data collection as detailed in the Methods section. For example, the target protein should have preferentially a coordinate structure file in the Protein Data Bank (PDB) or at least a full sequence for structure predictions. The UAA substitution site and the original NAA there were manually confirmed in the protein structure or sequence. UAA substitutions on linkers, peptides or proteins less than 50 residues were excluded, because their loose or small structures usually contain few stably interacting domains for studying the steric effects. The UAA substitution outcome (success or failure) was judged by different experimental methods as reported and further graded as direct, indirect or other related proofs. Direct proofs include mass spectrometry of UAA-incorporated proteins or critical peptide fragments [12], [15], [16], gels or blots confirming the expressions or non-expressions of UAA-incorporated proteins [17], or some well-established optical reporters [18]. Indirect proofs include miscellaneous results such as retaining protein functions [19] or enzymatic activities [20], or the virus package of UAA-incorporated viral proteins [21]. Other related proofs include UAA replacement using the auxotrophic strains [22] or UAA incorporations by the ligated UAA-dCA-tRNA method [23]. For failure substitutions, the researches had better demonstrate the UAA/aaRS/tRNA system was functional elsewhere [24] and the failure was mainly attributed to incompatible UAAs at current positions instead of a deficient system. When available, the UAA incorporation efficiency relative to wild-type was calculated to supplement the UAA incorporation outcome and efficiency >0.01 was considered as a successful incorporation.

After manually collecting the information of the target protein, the substitution site, the substituted NAA, the substituting UAA, the substitution outcome, the proof level and the related methods, we input it to the RPDUAA program to extract more information from files and format a record into the database. For each protein, three files were prepared: the cif file contains the protein structure information which could be downloaded from PDB or generated through structural predictions with AlphaFold2 or RoseTTAFold; the fasta file contains the protein sequence information; the xml file contains the multiple sequence alignment information obtained by a BLASTP query. By inputting the PDB ID of a protein, the RPDUAA program can automatically download and analyze the three files, extract the evolutional or structural information of interest for the protein, and process the key information into a fourth csv file for further use. The chemical formulae of all NAAs and UAAs were drawn in the Schrodinger Canvas software, which also supported calculating and exporting physiochemical properties and similarity matrix of NAAs and UAAs. In the first release, 20 NAAs, 172 UAAs, and 159 target proteins were registered in local libraries whose properties could be easily called by the RPDUAA program. Finally, the manually inputted and automatically extracted information of a NAA → UAA substitution was integrated by the RPDUAA program as a standard record in the database. At present, the database of experimentally verified UAA substitution sites has enrolled 1221 records from literature, comprising 1064 success records and 157 failure records (Fig. 1b). The database and related libraries are updatable and users can append their own UAAs, proteins or substitution records. For flexible use of the database, we also created several practical subsets, such as the direct proof subset, the amber suppression subset, the non-predicted PDB subset, and the non-viral protein subset. Machine learning on these featured subsets will give more perspectives (see Pages 48–51 of Supplementary Guide).

### 1.2 Establishment of the prediction model

We aimed to establish a model that can predict the outcome of UAA substitutions from observed evolutional, steric and physiochemical factors. The evolutional tolerance was evaluated by searching the protein sequence in BLAST and calculating the site entropy from the multiple sequence alignment result, which was conveyed by a xml file. The steric effects could be directly obtained from the protein structure in the cif or pdb format. Typical indexes of the steric effects include the relative accessible surface area of a residue [25], the half sphere exposure beta of a residue [26], and secondary structure types such as helix, strand and turn. The physiochemical properties exported from the Schrodinger Canvas software include atomic logP (AlogP), electrotopological states (Estate), polar surface area (PSA), molecular polarizability (Polar), hydrogen bond acceptors (HBA), hydrogen bond donors (HBD), rotatable bonds (RB), and molecular weight (MW). The differences between NAAs and UAAs on these physiochemical properties were calculated. The UAA similarity to the substituted NAA was based on the binary fingerprint of chemical formula and stored in a csv file. Besides, the expanded codon type and proof level were also considered. The full interpretations and preprocessing of these observed variables were detailed in Table 1 with their sources and categories annotated. The anticipated prediction model should simulate the tendency that substitutions at the evolutionally conserved or sterically hindered sites generally require UAAs with strictly similar properties to the original NAAs, while substitutions at the evolutionally mutable or sterically exposed sites may be more tolerant and less picky on UAAs.

**Table 1:** The variables or features used for machine learning in this study.

**Coefficient**	**Variable**	**Interpretation**	**Source**	**Category**
k1	entropy	the **site entropy** calculated from a BLASTP multiple sequence alignment, which correlates negatively with the site conserve level	xml file	evolutional tolerance
k2	rasa	the **relative accessible surface area** of the NAA residue in the protein, which ranges between 0 (fully buried) and 1 (fully exposed)	cif file	steric effects
k3	hsebup	the **half sphere exposure beta** in the stretched direction (radius = 12 Å) of the NAA side chain at a given site in the protein		
k4	hsebdn	the **half sphere exposure beta** in the opposite direction (radius = 12 Å) of the NAA side chain at a given site in the protein		
k5	sstype	the **secondary structure type** of the substitution sitetransformed to 3 dummy variables (isHelix, isStrand, isTurn)		
k6	uaasimi	the **UAA similarity** to the substituted NAA at a given site in the protein, based on the binary fingerprint of chemical formula	canvas	physiochemical changes
k7	△AlogP	the difference between UAA and NAA on **atomic logP** Preprocessing: △AlogP = AlogP_UAA_ – AlogP_NAA_		
k8	△Estate	the difference between UAA and NAA on **electrotopological states** Preprocessing: △Estate = Estate_UAA_ – Estate_NAA_		
k9	△PSA	the difference between UAA and NAA on **polar surface area** Preprocessing: △PSA = PSA_UAA_ – PSA_NAA_		
*k*10	△Polar	the difference between UAA and NAA on **molecular polarizability** Preprocessing: △Polar = Polar_UAA_ – Polar_NAA_		
*k*11	△HBA	the difference between UAA and NAA on the number of **hydrogen bond acceptors**. Preprocessing: △HBA = HBA_UAA_ – HBA_NAA_		
k12	△HBD	the difference between UAA and NAA on the number of **hydrogen bond donors**. Preprocessing: △HBD = HBD_UAA_ – HBD_NAA_		
k13	△RB	the difference between UAA and NAA on the number of **rotatable bonds**. Preprocessing: △RB = RB_UAA_ – RB_NAA_		
k14	△MW	the relative difference between UAA and NAA on **molecular weight** Preprocessing: △MW = (MW_UAA_ – MW_NAA_) / MW_NAA_		
k15	cdtype	the **expanded codon type** (TAG, TAA, TGA, or others)transformed to 3 dummy variables (isAmber, isOchre, isOpal)	other	other factors
k16	prooflv	the **proof level** (direct, indirect, or others) of a substitution recordtransformed to 2 dummy variables (isDirect, isIndirect)		

For all substitution records in the database, we collected and preprocessed the variables as listed in Table 1, which formed a feature matrix. The outcome of UAA substitutions (success or failure) is a binomial response variable, and we chose logistic regression as the prediction model (see Equation (1) below).

(1) $$\log\left(\frac{P}{1-P}\right)=b+\sum_{i=1}^{16}k_{\text{i}}\cdot V_{i}$$

Here, P represents the probability of successful UAA substitution at a given site in the protein; b denotes the bias of machine learning or the fitted intercept of logistic regression; k_i_ and V_i_ are the paired coefficients and variables as listed in Table 1, which were empirically considered to be crucial for UAA substitutions. The model mainly considered the protein structure information and amino acid properties to judge the possibility of incorporating one UAA at a given protein site. There are more variables affecting the UAA incorporation process, such as the codon context in the mRNA, kinetic property of protein biosynthesis, tRNA abundance, synthetase activity, and competition from release factors in host cells [27]. However, our model did not include these variables due to limited information in the literature and difficulties in quantifying and comparing these variables among different researches.

### 1.3 Performance of the prediction model

**Fig. 2:**
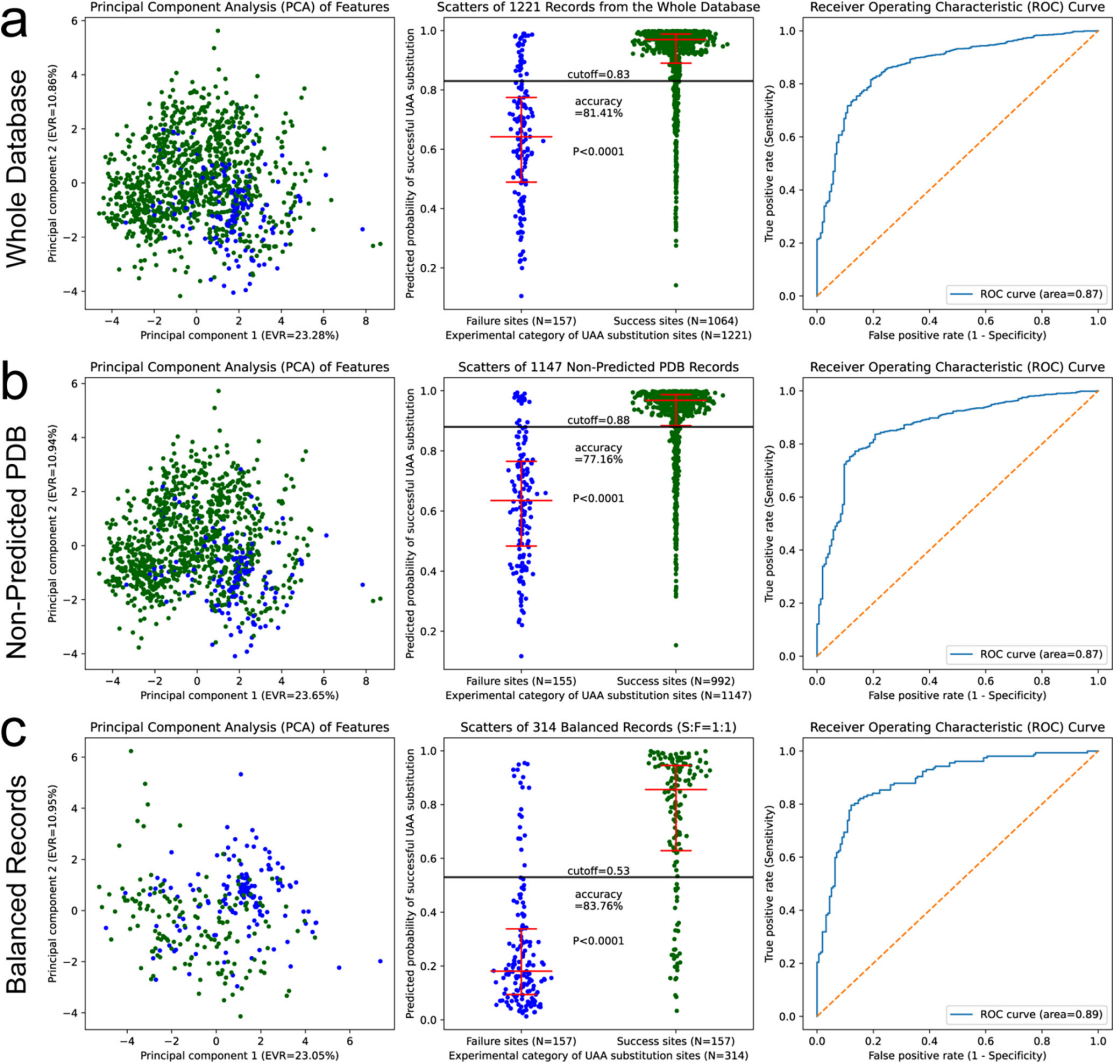
**Performance of the prediction model.** Principal component analyses of observed features (left panels), scatter plots of predicted probability (middle panels, where the error bars denote 25%, 50% and 75% quartiles, and P values for the Mann-Whitney test) and receiver operating characteristic curves (right panels) were shown for (a) the whole database, (b) the non-predicted PDB subset, and (c) the balanced subset.

Next, we embedded the prediction model in the RPDUAA program and tested its performance on the whole database using three different methods (Fig. 2a). Principal component analysis (PCA) of the feature matrix showed a splitting trend between success and failure records, implying that success UAA substitutions may possess a different set of features from failure ones. However, the clusters of the two groups still overlapped seriously on the PCA graph, partly because of the low explained variance ratios (EVR1 + EVR2 < 50 %) by principal components 1 and 2 in the two axes. After training the prediction model with the whole database, we plotted the predicted probability of successful UAA substitutions. As expected, success records exhibited significantly higher probabilities than failure records, and a probability cutoff might work in separating the majority of success records and failure records. After optimizing the probability cutoff to 0.83 by maximizing the sum of sensitivity and specificity, the prediction model yielded an accuracy of 81.41 % when trained and tested with the whole database. The receiver operating characteristic (ROC) curve was used to determine the overall diagnostic capability of the prediction model on the UAA substitution outcome. For the whole database, the area under the ROC curve was 0.87, indicating a reliable diagnostic capability.

The majority of target proteins in the database were downloaded from PDB, while a minority were predicted from the protein sequence by AlphaFold2 or RoseTTAFold due to the lack of a PDB coordinate. We created a non-predicted PDB subset containing all records with a PDB coordinate but excluding those with in silico predicted protein structures. When the model was trained and tested by this subset, a similar splitting trend was seen on the PCA graph, the prediction accuracy at the optimal cutoff was 77.16 %, and the ROC area was 0.87 (Fig. 2b). Moreover, we tested the model with some featured subsets, such as the amber suppression subset, the miscellaneous proofs subset, or the non-redundant subset (see Page 50 of Supplementary Guide), which also demonstrated similar performances.

The whole database and most of its subsets were biased with overwhelming success records than failure ones, partly due to the fact that the literature tended to report more successes than failures. As a result, the optimal cutoff was much higher than 0.50 and the accuracy fluctuated heavily depending on the cutoff. However, the ROC curve was stable and the ROC area was above 0.85 for most subsets, which can serve as a golden standard of the model performance. To handle the bias, we created a balanced subset by resampling equal numbers of success records to failure records from the whole database. For such a balanced subset, the PCA graph showed evident clustering, the probability scatters showed distinct distributions between success and failure groups, the optimal cutoff went to 0.53, the accuracy was 83.76 %, and the ROC area was 0.89, which all supported good performances of the prediction model (Fig. 2c).

### 1.4 Stability of the prediction model

**Fig. 3:**
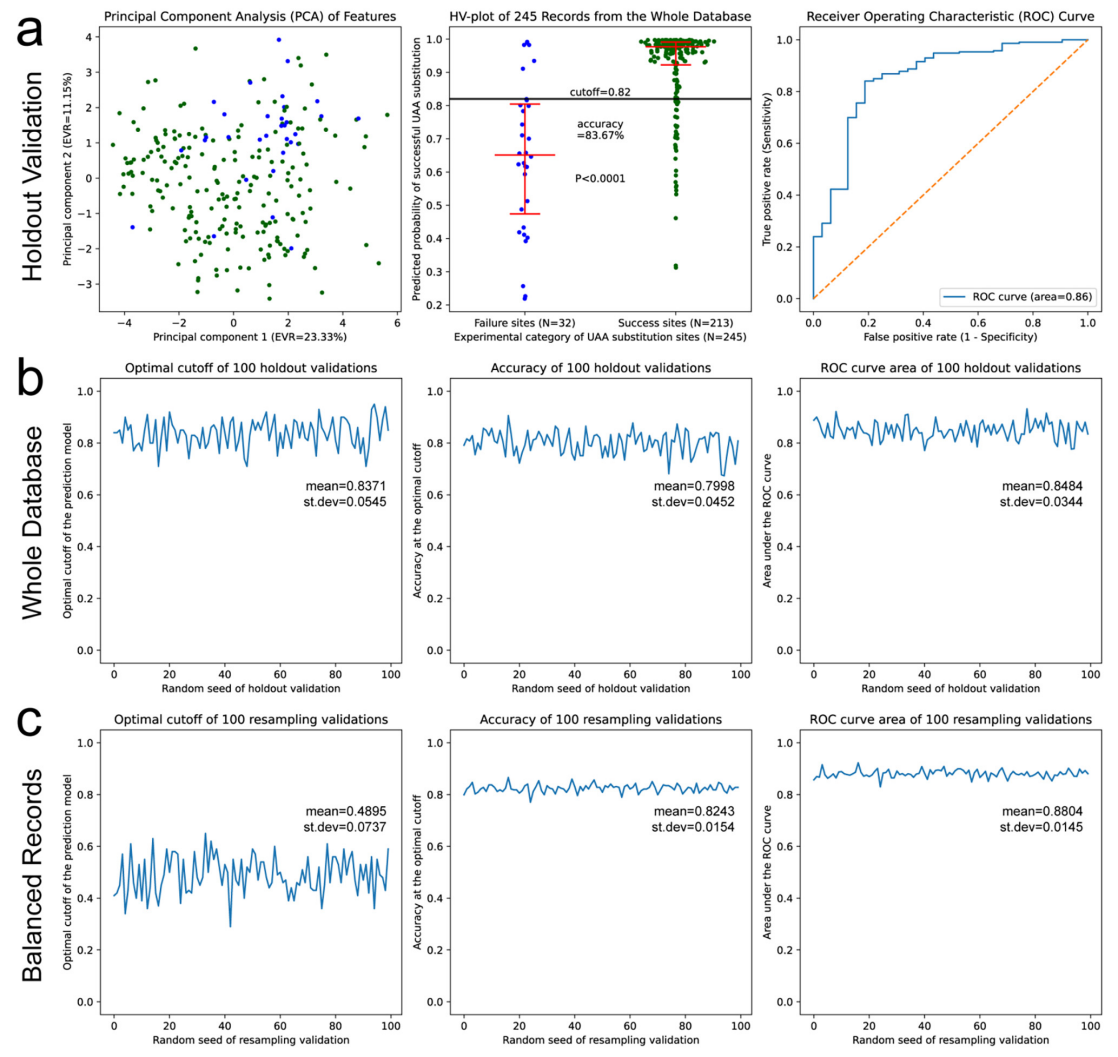
**Stability of the prediction model.** (a) Performance in a typical round of holdout validation. Graph definitions, error bars, and the statistical test are the same as previously described. (b) Changes of the optimal cutoff, accuracy and ROC area in 100 rounds of holdout validations using the whole database. (c) Changes of the optimal cutoff, accuracy and ROC area in 100 rounds of resampling validations using balanced subsets.

To confirm the stability of the prediction model, we performed holdout validations by randomly picking 80 % records from the whole database as training data and using the rest 20 % records as testing data to plot the graphs. In a typical round of holdout validation (Fig. 3a), we observed splitting trends on the PCA graph and the probability scatters, with an accuracy of 83.67 % and a ROC area of 0.86. Then, we performed 100 rounds of holdout validations and monitored the changes of the key reporting indexes (Fig. 3b). The optimal cutoff fluctuated around 0.84, the accuracy fluctuated around 0.80, and the ROC area fluctuated around 0.85 with standard deviations smaller than 0.06, demonstrating the stable performances of the prediction model.

For the stability using balanced records, we randomly resampled equal numbers of success records to failure records from the database for 100 times to make 100 balanced subsets. By training and testing the prediction model with these balanced subsets, the optimal cutoff normally ranged between 0.4 and 0.6 with an average of 0.49, the accuracy averaged at 0.82, and the ROC area averaged at 0.88 with small standard deviations, which supported stable performances of the prediction model on balanced records.

### 1.5 Timesplit validation of the prediction model

**Fig. 4:**
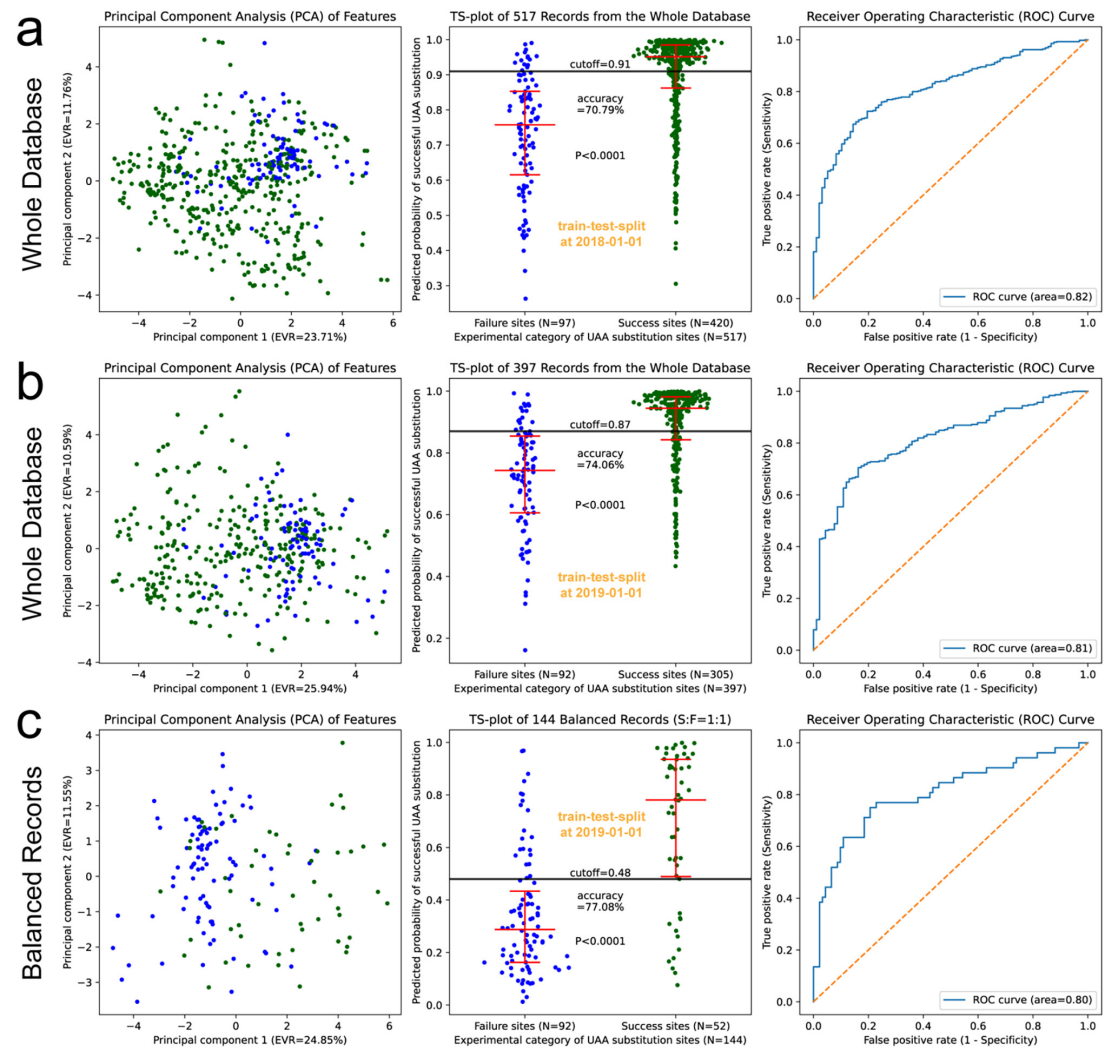
**Timesplit validation of the prediction model.** (a) Train-test-split at the time point 2018-1-1 for the whole database. (b) Train-test-split at the time point 2019-1-1 for the whole database. (c) Train-test-split at the time point 2019-1-1 for a balanced subset. Records before the split time point were used to train the model, while records after that time point were used to test the model and plot the graphs. Graph definitions, error bars, and the statistical test are the same as previously described.

Inspired by the parameter of max_template_date in AlphaFold2, we decided to include the function of train-test-split at a time point in the RPDUAA program, termed as the timesplit validation. By training the model with early records published before 2018-1-1, and testing the model and plotting the graphs with late records published after 2018-1-1, we observed splitting trends both on the PCA graph and probability scatters, with a prediction accuracy of 70.79 % and an ROC area of 0.82 (Fig. 4a). By performing train-test-split at another time point, 2019-1-1, similar performances were observed with a prediction accuracy of 74.06 % and an ROC area of 0.81 (Fig. 4b), which means that early records of UAA substitutions can predict late records reliably using the prediction model in the RPDUAA program.

Besides the whole database, the timesplit validation could also be performed on its subsets, as long as the training data and testing data after splitting are both enough. We performed train-test-split at 2019-1-1 on a balanced subset, which showed a prediction accuracy of 77.08 % and an ROC area of 0.80 (Fig. 4c). Unlike the whole database and most of its subsets whose optimal cutoffs were usually above 0.80, the optimal cutoff for a balanced subset was near 0.50. When predicting the outcome of UAA substitutions using the RPDUAA program, the predicted probability of successful UAA substitutions should to be compared with the optimal cutoff of the chosen subset (∼0.49 for balanced subsets, ∼0.84 for the whole database or other subsets). In summary, the timesplit validations proved reliable capabilities (ROC area > 0.80) of the prediction model.

### 1.6 Experimental validation of the prediction model

**Fig. 5:**
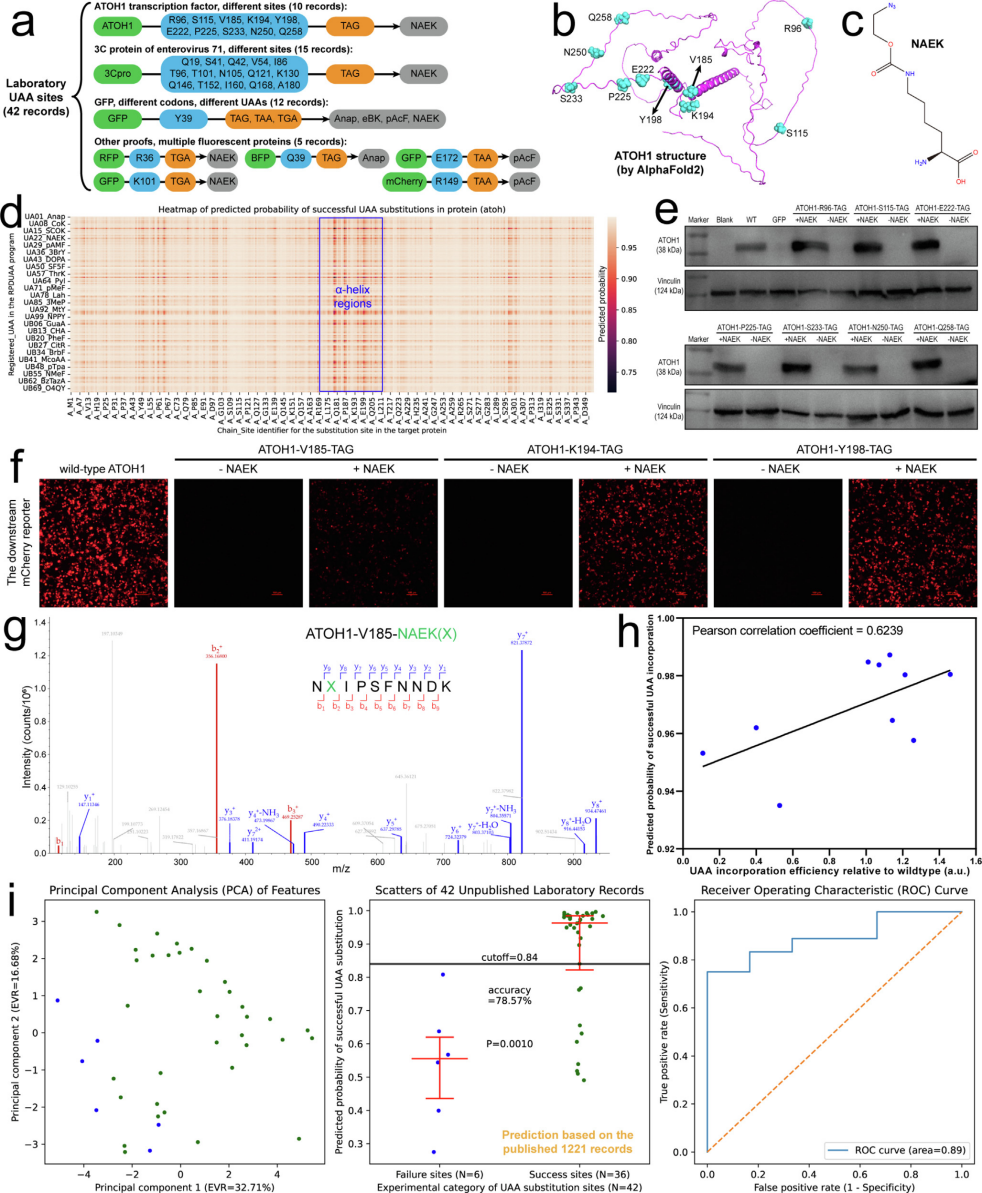
**Experimental validation of the prediction model.** (a) Composition of the laboratory database of freshly-designed UAA-incorporated proteins. (b) Structure of the ATOH1 protein predicted by AlphaFold2. (c) Chemical formula of NAEK. (d) Full probability matrix for all UAAs substituting any site of the ATOH1 protein. The probability was predicted by the RPDUAA program using the whole database. (e) Western blot images of NAEK-incorporated ATOH1. The NAEK-dependent ATOH1 expressions were an indicator of successful UAA substitution at a site. (f) Fluorescence images of a downstream mCherry reporter confirming NAEK incorporations in ATOH1 in HEK293T cells. Scale bar, 100 μm. (g) Mass spectrometry of fragments with NAEK incorporated at the V185 site of ATOH1. (h) Correlation plot between the predicted probability and UAA incorporation efficiency for the 10 sites of ATOH1. (i) Performances of the prediction model when trained with the whole database from the literature and tested with the freshly-prepared laboratory records. Graph definitions, error bars, and the statistical test are the same as previously described.

To further demonstrate the capability of the model in predicting UAA substitution outcomes, we designed 4 sets of UAA-incorporated proteins (Fig. 5a) and verified their outcomes experimentally, which made a small laboratory database of 42 records covering different proteins, sites, codons and UAAs. We selected 10 sites on the AOTH1 protein (Fig. 5b), and substituted each of them with NAEK (Nε-2-azidoethyloxycarbonyl-l-lysine, Fig. 5c). Using the RPDUAA program, we predicted the probability of successful UAA substitutions across all sites on the ATOH1 protein (Fig. 5d). Due to the relatively loose structure of ATOH1, the predicted probabilities were generally above 0.70 for most sites, implying high tolerances for UAA substitutions across the ATOH1 protein. However, the core structured regions (α-helix between residues 170–210) of the ATOH1 protein showed smaller probabilities than other unstructured regions, since the former contain more residue interactions that may hinder UAA substitutions. As demonstrated by experiments, NAEK was successfully incorporated into all 7 sites selected from the unstructured regions (Fig. 5e). Among the 3 sites in the core structured regions, K194 and Y198 tolerated NAEK substitutions well, but V185 showed a relatively weak efficiency (Fig. 5f). The mass spectrometry further confirmed the NAEK incorporation at the V185 site of ATOH1 (Fig. 5g). After calculating the UAA incorporation efficiency by grayscale analysis, we found that the predicted probability by the RPDUAA program showed a positive correlation (R = 0.6239) with the UAA incorporation efficiency (Fig. 5h). Besides ATOH1, we also tested UAA substitutions on multiple structure-solved proteins including 3Cpro (PDB ID: 3osy, a viral protein), GFP (PDB ID: 1gfl), BFP (PDB ID: 3 m24), RFP (PDB ID: 1zgo) and mCherry (PDB ID: 2h5q). We used the reported 1221 records (the public whole database from literature) as training data, and the 42 freshly-prepared records (the private laboratory database) as testing data to verify the performance of the prediction model (Fig. 5g). The resulting ROC area was 0.89, indicating that the prediction model had a good diagnostic capability in discriminating success records from failure ones. At the optimal cutoff of 0.84, the prediction model showed an accuracy of 78.57 %. The false positives were mainly observed on the 3osy viral protein, where amber codons may recover to normal codons during virus propagation according to a relevant research [21] but UAA incorporations were indirectly reported by virus package in this experiment. Excluding this viral protein which was proved by an insensitive method, the accuracy could exceed 90 % as tested with other normal proteins. For practices of virtual screening, users can customize or elevate the probability threshold to 0.90 or 0.95, which will give a dense set of successful UAA substitutions for high confidence.

### 1.7 Prediction of the UAA incorporation efficiency

Since the UAA incorporation efficiency is an index more meaningful than the predicted probability, we inset a preliminary function in the RPDUAA program for machine learning directly on the reported efficiencies. The UAA incorporation efficiency is defined as the relative ability to incorporate a UAA at a site compared with incorporating a wild-type NAA there, which is a continuous variable calculated as the expression ratio of UAA-incorporated proteins and wild-type proteins based on the protein yield or grayscale analysis. In the current database, 124 records possess an exact UAA incorporation efficiency calculated from the reported protein yield (the subset with exact yield), while 351 records possess a coarse UAA incorporation efficiency that allows both the protein yield and grayscale analysis of gels or fluorescence (the subset with coarse yield). The former subset has a better dataset quality but a smaller sample size than the latter subset.

**Fig. 6:**
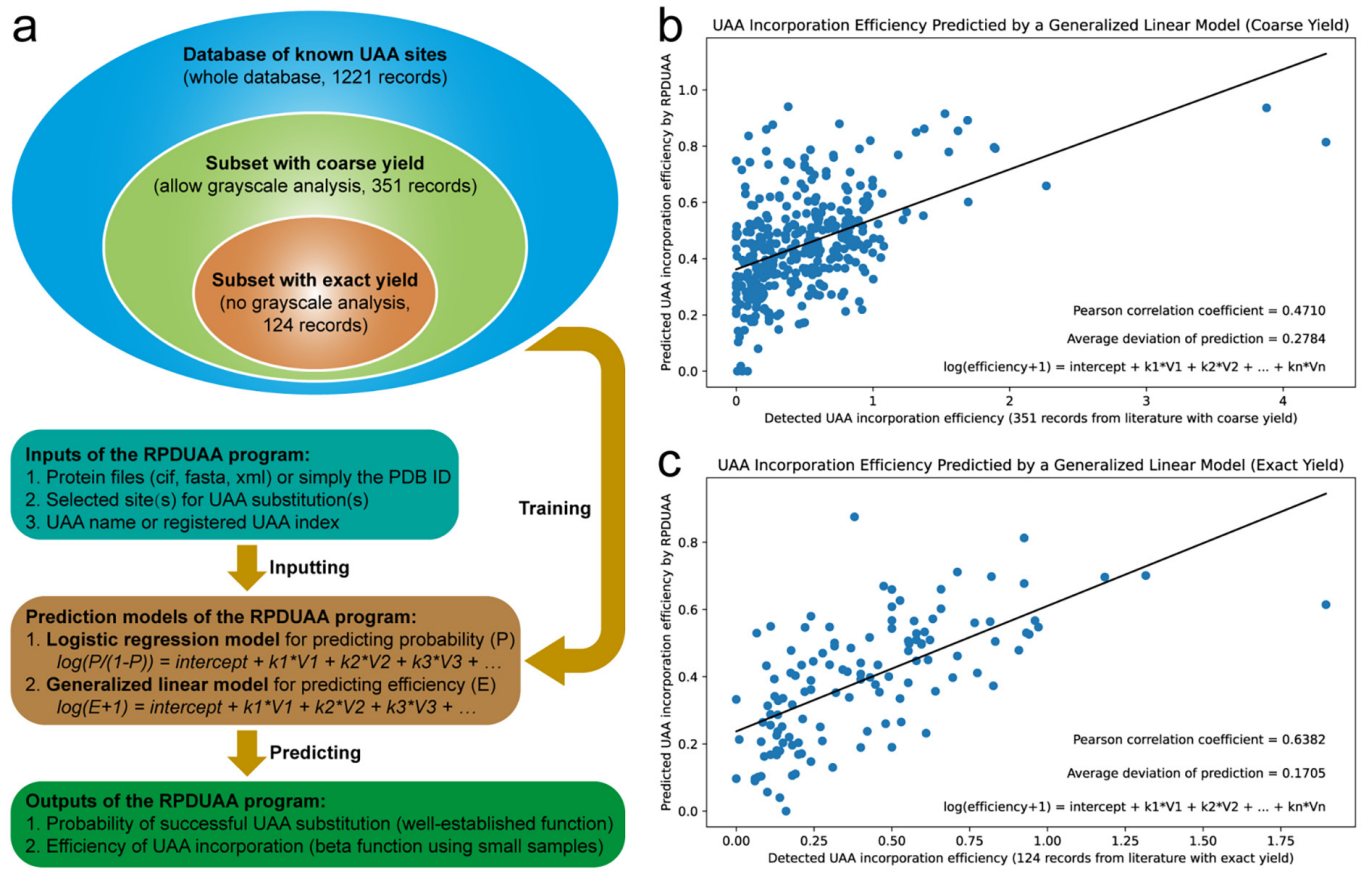
**Prediction of the UAA incorporation efficiency.** (a) Subsets with coarse or exact yields in the database and prediction models in the RPDUAA program. (b) Performance of the model using the subset of coarse yield. (c) Performance of the model using the subset of exact yield. The solid line represents the linear regression between the predicted and detected UAA incorporation efficiencies, and the Pearson’s correlation coefficient R is reported.

Using the same set of variables as listed in Table 1, we adopted a generalized linear model with the log(E + 1) preprocessing to predict the UAA incorporation efficiency (Fig. 6a). When the model was trained by the subset with coarse yield, the predicted UAA incorporation efficiency correlated positively with the detected UAA incorporation efficiency by experiments, but the correlation was weak (R = 0.4710), which was partly attributed to the semi-quantification nature of grayscale analysis (Fig. 6b). When the model was trained by the subset with exact yield, the positive correlation became clearer and the correlation coefficient improved fairly (R = 0.6382, Fig. 6c). However, considerable deviations (±0.1705 or larger) still existed between the predicted and detected UAA incorporation efficiencies using this pilot model. While the probability-based prediction of the UAA incorporation outcome is well established and systemically evaluated in the RPDUAA program, it is still challenging to predict the UAA incorporation efficiency for a general UAA incorporation process, which may require more high-quality samples and process-affecting variables beyond Table 1 such as the codon context in the mRNA, kinetic property of protein biosynthesis, tRNA abundance, synthetase activity, and competition from release factors in host cells.

## 2 Discussion

This research systematically summarized the reported UAA-incorporated proteins in the last wo decades into a database for machine learning, and established a reliable prediction model to formulate the rules of UAA substitutions. The RPDUAA program supported direct analyses of protein information in cif, fasta and xml files, and registered 172 UAAs in its first release (see Supplementary Guide, Pages 11–43). Users can add their own target proteins or UAAs to the libraries, append new substitution records to the database, test the performance of the prediction model with the whole database or one of its subsets, predict the probability of successful UAA substitutions, and optimize their UAA-incorporated proteins conveniently in the RPDUAA program. For example, based on the predicted probability and the full heatmap (Fig. 5d), uses can find the optimal substitution sites on a given protein for a given UAA (the fixed UAA strategy), or find the optimal UAAs that may substitute a given site on a given protein (the fixed site strategy). In previous studies, the selections of UAAs and substitution sites were mostly based on experience or mechanical screening with laborious experiments. The RPDUAA program could automate the selections based on artificial intelligence and virtual screening, which is a useful tool for the rational design of UAA-incorporated proteins. As proved by the timesplit validations (Fig. 4) or experimental validations (Fig. 5), the RPDUAA program exhibited a reliable predictive capability. By setting a probability threshold at the optimal cutoff of the chosen subset (∼0.49 for balanced subsets, ∼0.84 for the whole database or other subsets) or higher (such as 0.90 or 0.95), users can get the high-confidence candidates of UAA-incorporated proteins for future investigations.

When evaluating the performance of the prediction model, we noticed that the range of predicted probability was wide for both success and failure groups (Fig. 2, middle panels), leading to high rates of false negatives and false positives. The false positives in the failure group may be related to the indirect proofs judged from virus package or protein functions, in which the successful UAA-incorporated proteins might be mistaken into the failure group due to the loss of virus packaging competency or other protein functions [28]. The false negatives in the success group could be explained by the limitation of predictions based on rigid structures. The RPDUAA program may suggest some sites which are less suitable for UAA substitutions judging from the rigid protein structure (the static cif file), but the real protein may be more flexible and tolerant for UAA substitutions. Specially for viral proteins, some unstable amber codons may recover to normal codons during virus propagation and UAA incorporations may be misreported even for those deprecated sites, which is also a cause of false negatives. The RPDUAA program was less effective in predicting UAA-incorporated viral proteins than normal proteins, as proved by the performance on the viral protein subset (ROC area = 0.70) and the non-viral protein subset (ROC area = 0.81) in Page 51 of Supplementary Guide. Nonetheless, the prediction model in the RPDUAA program can well discriminate the majority of success records from that of failure ones by automatically setting an optimal cutoff, as indicated by the horizontal cutoff line and bars of the 25 % and 75 % quartiles on each probability graph.

The PRDUAA program predicts the UAA substitutions from several factors like evolutional tolerance, steric effects and physiochemical changes. In the aspect of steric effects, we extracted the information of residue exposure or solvent accessibility directly from the protein structure in the cif or pdb format. However, some exposed residues may become buried when the protein forms complexes with other structures or packages with other viral proteins, which will give misleading information for UAA substitution prediction. This could be solved by directly inputting the protein complex for prediction. If the protein complex is not available in PDB, it can be created from docking servers like ZDOCK [29], PatchDock or SymmDock [30], or from multimer protein structure predictions in AlphaFold2. After transforming to the cif format, the protein complex can be analyzed by the RPDUAA program for UAA substitution predictions. The “Dock/Fold + RPDUAA” pipeline supports a much larger library of complexed proteins beyond PDB and offers more accurate information of steric effects for UAA substitution predictions. The RPDUAA program also embedded some extra functions, such as using absolute differences when preprocessing physiochemical changes, and appending some in-lab records to predict from published records to unpublished ones (see Pages 57–59 of Supplementary Guide). Besides predicting the probability of successful UAA substitution, the RPDUAA program also contained a preliminary function for predicting the UAA incorporation efficiency. However, the UAA incorporation into proteins is a multivariate problem far beyond those listed in Table 1, and the accurate prediction of the UAA incorporation efficiency may require more critical variables, including the codon context in the mRNA, the kinetic properties, and the specificity of the synthetase. Since these variables are not always available in the literature and some of them are hard to quantify or compare between different researches, we compromised our model to the protein structure and amino acid property level in the RPDUAA program.

The site-specific UAA incorporation technique through genetic code incorporation has been widely applied in protein engineering and new drug design. ARX788, an antibody-drug conjugate that utilizes UAA-enabled conjugation, has demonstrated potent and selective activities in HER2-low and T-DM1-resistant breast and gastric cancers [31]. UAA-incorporated replication-incompetent viruses have been used as candidate vaccines [28]. The introduction of a proper UAA may give rise to new protein drugs [7], and the optimal substitution sites on the protein drug also need optimization. To date, about 180,000 solved protein structures have been deposited in PDB, each protein may contain hundreds or thousands residues, and approximately 200 UAAs have been reported in literature, multiplying to billions of candidate UAA substitutions. The virtual screening functions provided by the PRDUAA program may help cut the experimental cost and shorten the development period of UAA-incorporated protein drugs. Moreover, the UAA incorporation technique has been developed as a therapeutic strategy for nonsense mutation diseases [17], and the RPDUAA program here may supplement this strategy in preselecting the optimal UAA system for personalized therapies of nonsense mutations that occur at different sites of a protein.

Protein structure prediction tools such as AlphaFold2 have made impressive progresses [32], but in silico tools involving UAAs are still rare. To date, AlphaFold2 and RoseTTAFold have not used UAAs as basic residues. The CHARMM-GUI platform supports UAA substitutions for PDB manipulations, free energy calculations or molecular dynamics simulations [33], which are vastly in silico and not experimentally supervised by UAA incorporation outcomes or efficiencies. The RPDUAA program we proposed here is based on the generalized UAA substitutions supervised by the reported experimental results (Fig. 1a), which supports the prediction of any UAA substituting any site on any protein from existing experimental proofs, and its libraries of UAAs and proteins are also extensible to include a broad spectrum of UAAs and proteins as the users need. Besides, the database of UAA-incorporated proteins or known UAA substitution sites established in this research may serves as a potential resource for UAA-involved protein translations, structure-based computational biology, machine learning on proteins with special residue modifications, and future structure predictions of UAA-incorporated proteins.

## 3 Methods

### 3.1 Preparation of the database of known UAA sites

We adhered to several criteria of inclusions and exclusions when preparing UAA substitution records from literature: (1) Research articles published between 2001 and 2021 were considered, while records in reviews were traced back to their original research articles. (2) The article should provide definite information of the target protein, the substitution site, the substituted NAA, the substituting UAA, and the substitution outcome. (3) For the target protein, a PDB coordinate was normally required and strongly recommended. If the PDB coordinate was not available, the full protein sequence was used to predict the required protein structure with RoseTTAFold (https://robetta.bakerlab.org/) or AlphaFold2 instead. Protein structure predictions were used sparingly. (4) UAA incorporations through protein biosynthesis or genetic code expansion were considered as the main study object. UAA incorporations using auxotrophic strains or ligated UAA-dCA-tRNA method were considered as other related proofs, while those through solid phase peptide synthesis, post-translational modifications or protein semi-synthesis were not related. (5) Peptides or small proteins less than 50 residues were excluded. (6) UAA substitutions that lie in the amorphous linker regions of fusion proteins or any user-defined protein constructs were generally not considered due to the lack of definite structure information. (7) UAA incorporations in viral proteins were included, but specially noted. (8) The UAAs should be l-form just like the NAAs, while d-form UAAs were not considered.

The cif, fasta, xml and csv files of the involved target proteins were uniformly named with the PDB ID and placed into a “proteins” folder as the protein library. The chemical formulae of NAAs and UAAs were drawn in the Canvas software (version 3.5 in Schrodinger 2018 suite) as the NAA&UAA library, with the similarity matrix and physiochemical properties of NAAs and UAAs exported to csv files and placed into a “residues” folder to be easily called by the RPDUAA program. Finally, the key information of UAA substitutions were collected into the database (also a csv file in the “residues” folder) using the RPDUAA program. Besides, users can automate the preparations of cif, fasta, xml and csv files of a protein just by inputting its PDB ID with an underscore prefix (e.g., “_6mh2” to analyze the Herceptin protein) in the RPDUAA program.

### 3.2 Development of the RPDUAA program in Python

The RPDUAA program was developed in Python (version 3.9) and PyCharm (version 2021.1.1). To analyze protein sequences and structures, BioPython (version 1.78) was used. Secondary structures [34] were extracted by DSSP (version 3.0.0) [35], an external executable (https://swift.cmbi.umcn.nl/gv/dssp/HTML/distrib.html). Numpy (version 1.19.5) and Pandas (version 1.2.4) were used for the scientific computations and dataframe manipulations. Machine learning and logistic regression were performed using Scikit-learn (version 0.24.2). Reporting graphs were made by Matplotlib (version 3.4.2) and Seaborn (version 0.11.2). An executable form of the RPDUAA program is available at Github (https://github.com/ZHR2PKU/RPDUAA) with necessary files and folders, which is recommended to run on the Windows 10 platform (64 bit).

### 3.3 Performance and stability of the prediction model

For all records in the database of known UAA sites, we computed and collected the variables in Table 1 into a feature matrix. Besides the whole database, we also gathered some records into special subsets, such as the non-predicted PDB subset and amber suppression subset. The balanced subset enrolled all the failure records and equal numbers of success records that were randomly resampled from the database. Logistic regression was performed between the UAA substitution outcome (success or failure) and the feature matrix, using the whole database, special subsets or randomly selected training records. General performances were evaluated using a subset simultaneously as training data and testing data. PCA graphs of the feature matrix, scatter plots of the predicted probability of successful UAA substitutions, and ROC curves were reported along with some key indexes such as the optimal cutoff, prediction accuracy and ROC area.

For holdout validations, 80 % records were randomly selected as the training data from the whole database and the rest 20 % records as the testing data. The train-test-splits were repeated for 100 times using random seeds 0–99, and the key indexes (optimal cutoff, prediction accuracy and ROC area) were collected for each round to evaluate the stability of the prediction model on the whole database. Besides, the train-test-split can also be performed at a time point like 2018-1-1, namely the timesplit validation.

Specially for the balanced subset, equal numbers of success records to failure ones were randomly resampled from the whole database for 100 times using random seeds 0-99, and combined with all the failure records to prepare 100 balanced subsets. Each balanced subset was used simultaneously as training data and testing data in each round to get the key indexes (optimal cutoff, prediction accuracy and ROC area). Fluctuations of the key indexes in 100 rounds could reflect the stability of the prediction model on balanced subsets.

For predictions of the UAA incorporation efficiency, a generalized linear model was adopted, which can be achieved by the 4e command in the main menu of the RPDUAA program. The model was trained by those records with coarse or exact protein yields in the database. The performance of the model was evaluated by the positive correlation between the predicted UAA incorporation efficiency by the RPDUAA program and the detected UAA incorporation efficiency by experiments.

### 3.4 Predictions of UAA substitutions for a given protein

The PRDUAA program supports predicting the probability of successful UAA substitutions for all registered UAA substituting any site on a given protein. If the protein has a PDB coordinate, users can automatically prepare its four files (cif, fasta, xml, and csv) just by inputting its PDB ID with an underscore prefix (e.g., “_6mh2” for the Herceptin protein) in the RPDUAA program, which is quite convenient. If the protein does not have a PDB coordinate, users need to prepare the protein structure from AlphaFold2 or RoseTTAFold, or use some docking servers to export the protein complex, and finally transform the output pdb files to cif files. Write the protein sequence in a fasta file, visit NCBI (https://blast.ncbi.nlm.nih.gov/Blast.cgi), BLAST the fasta sequence of the protein and export the alignment results into a xml file. Rename the cif, fasta and xml files uniformly with four lowercase letters or numbers like the PDB ID, place them into the “proteins” subfolder, and use the RPDUAA program to analyze the protein into a fourth csv file. In this way, the protein was registered into the protein library and ready for further predictions.

After preparing the four files (cif, fasta, xml, and csv) of a given protein, users can predict the probability of UAA substitutions in the RPDUAA program. The prediction model can be trained by the whole database of known UAA sites (recommended by default), the balanced subset (cutoff around 0.5), or any other subsets. The RPDUAA program provides three practical strategies of prediction: (1) For a given protein and a given UAA, find the optimal substitution sites; (2) For a given protein and a given substitution site, find the optimal UAAs; (3) Full probability matrix for all UAAs scanning all sites of a given protein. After choosing a subset, selecting the full probability matrix strategy, and inputting the filename of the protein, a heatmap will emerge showing the predicted probability of successful UAA substitutions for all registered UAA substituting any site on the protein. Meanwhile, a detailed csv report containing the full probability matrix will be exported into the “predictions” subfolder. Users can sort the probability or set any threshold according to the optimal cutoff of the chosen subset (∼0.49 for balanced subsets, ∼0.84 for the whole database or other subsets).

### 3.5 Experimental validation using different UAA-incorporated proteins

The tested target proteins include: the human ATOH1 transcriptional factor (UniProt ID: Q92858); the 3C protease of human enterovirus 71 (3Cpro, PDB ID: 3osy); the green fluorescent protein (GFP, PDB ID: 1gfl); the blue fluorescent protein (BFP, PDB ID: 3 m24); the red fluorescent protein (RFP, PDB ID: 1zgo); mCherry (PDB ID: 2h5q). The tested UAAs include: 3-(6-acetylnaphthalen-2-ylamino)-2-aminopropanoic acid (Anap); p-acetyl-l-phenylalanine (pAcF); Nε-Boc-l-lysine (eBK); Nε-2-azidoethyloxycarbonyl-l-lysine (NAEK). The tested UAA incorporation systems include: AnapRS-tRNA^EcLeu^ for Anap incorporation; OMeYRS-tRNA^EcTyr^ for pAcF incorporation; MbPylRS-tRNA^MbPyl^ for eBK incorporation; MmPylRS-tRNA^MmPyl^ for NAEK incorporation. The expanded codons of UAAs may use TAG, TAA or TGA.

Four sets of UAA-incorporated proteins (42 records in total) were designed: ATOH1-(R96, S115, V185, K194, Y198, E222, P225, S233, N250, Q258)-TAG → NAEK; 3Cpro-(Q19, S41, Q42, V54, I86, T96, T101, N105, Q121, K130, Q146, T152, I160, Q168, A180)-TAG → NAEK; GFP-Y39-(TAG, TAA, TGA)→(Anap, eBK, pAcF, NAEK); Other fluorescent proteins including RFP-R36-TGA → NAEK, GFP-K101-TGA → NAEK, BFP-Q39-TAG → Anap, GFP-E172-TAA → pAcF and mCherry-R149-TAA → pAcF.

Defined sites on the plasmids of these proteins were mutated to the assigned stop codon. Then the plasmids of ATOH1, GFP, BFP, RFP and mCherry were co-transfected into HEK293T cells with the plasmids of the UAA incorporation system, respectively. The medium was supplemented with or without 1 mM UAAs to test the UAA dependency. The ATOH1 expressions was detected by western blot (ATOH1 antibody: 21215-1-AP, proteintech, 1:3000) or a downstream mCherry reporter 48 h post-transfection. The expressions of GFP, BFP, RFP and mCherry were detected by fluorescence microscopy. For 3Cpro, plasmids of the whole enterovirus 71 genome were used with 3Cpro bearing an amber codon at defined sites. HEK293T cells stably harboring the NAEK incorporation system were transfected with the linearized enterovirus 71 plasmids and cultured with or without 1 mM NAEK until 90 % cytopathic effects. The NAEK-dependent cytopathic effects or virus package could indicate successful NAEK incorporations at defined sites of 3Cpro. The NAEK incorporation into the ATOH1 protein was confirmed by mass spectrometry (Thermo Lumos). The UAA incorporation efficiency were analyzed by grayscale of gels or fluorescence and normalized by wild-type.

### 3.6 Statistical analysis

The sample size of the database (1221 records indeed) was predetermined to larger than 10 times the column number of the feature matrix (Table 1). Some criteria of inclusions and exclusions were used to improve the database quality as aforementioned. The records were classified into the success group (1064 records) or the failure group (157 records) according to the experimental proofs. After machine learning on the database, the confusion matrix and the ROC curve were generated, along with the accuracy, sensitivity and specificity of the prediction model. For the predicted probability of successful UAA incorporations, the 25 %, 50 % and 75 % quartiles were annotated on the scatter plots, and the nonparametric Mann-Whitney test was performed between success and failure groups with two-sided P values reported. P < 0.05 was considered as statistically significant, while P < 0.0001 was considered as extremely significant. All statistical tests were two-sided and performed with the Scipy stats module (version 1.7.0) in Python.

## 4 Code availability

The RPDUAA program and its code are available at Github (https://github.com/ZHR2PKU/RPDUAA).

## CRediT authorship contribution statement

**Haoran Zhang:** Conceptualization, Methodology, Software, Formal analysis, Data curation, Visualization, Writing – original draft. **Zhetao Zheng:** Investigation, Validation, Data curation. **Liangzhen Dong:** Investigation, Validation, Data curation. **Ningning Shi:** Writing – review & editing. **Yuelin Yang:** Writing – review & editing. **Hongmin Chen:** Writing – review & editing. **Yuxuan Shen:** Writing – review & editing. **Qing Xia:** Conceptualization, Project administration, Supervision, Writing – review & editing, Funding acquisition, Resources.

## Declaration of Competing Interest

The authors declare that they have no known competing financial interests or personal relationships that could have appeared to influence the work reported in this paper.

## Appendix A Supplementary data

Supplementary Data

Supplementary Guide
