# Supplementary material for: Rational incorporation of any unnatural amino acid into proteins by machine learning on existing experimental proofs
Source: Comput Struct Biotechnol J. 2022 Sep 5;20:4930–41. doi: 10.1016/j.csbj.2022.08.063 (PMC9472073; doi:10.1016/j.csbj.2022.08.063)
Supplement: Supplementary Guide [file mmc2.pdf]

Guide for the RPDUAA program

```
*******************************************************************************
*** Rational Protein Design with Unnatural Amino Acids (RPDUAA, version 1.0) ***
*******************************************************************************
Function: Prediction of High-Confidence Sites for UAA Substitutions on a Protein
 Author: Haoran Zhang    Tutor: Prof. Qing Xia    Affiliation: Peking University
It is strongly recommended to use a monospaced font, such as Courier or Consolas
*******************************************************************************
// Main Menu:
       [1] Analyze Protein Structures (cif + fasta + xml --> csv)
       [2] Show the List of Available Unnatural Amino Acids (UAAs)
       [3] Manage the Database of Experimentally Verified UAA Sites
       [4] Predict High-Confidence Sites for UAA Substitutions
       [5] About the RPDUAA Program and How to Cite/Use RPDUAA
```

Brief description:

RPDUAA is a virtual screening program for the rational protein design (RPD) with unnatural amino acids (UAAs) based on reported experimental substitutions. As the Main Menu of RPDUAA shows, you can analyze protein structures, get the list of available UAAs, manage the database of experimentally verified UAA sites (a local database that collected known UAA substitutions/incorporations in proteins from literature), and predict high-confidence sites for UAA substitutions in a protein by machine learning on the UAA substitution database and protein structures.

Developer information:

> Full Name: Rational Protein Design with Unnatural Amino Acids
> Short Name and Version: RPDUAA (version 1.0, on 2021-10-1)
> Author and Email: Haoran Zhang (henryzhang@hsc.pku.edu.cn)
> Tutor and Email: Professor Qing Xia (xqing@hsc.pku.edu.cn)
> Affiliation: School of Pharmaceutical Sciences, Peking University
> Address: Xueyuan Road 38, Haidian District, Beijing 100191, China

> Environment: Source code written in Python 3.9.5, Windows Platform
> GitHub Link of the RPDUAA program: https://github.com/ZHR2PKU/RPDUAA

Content of the guidebook:

Chapter 1: Quick Start for Using RPDUAA
Chapter 2: List of Available UAAs and Their Formulae
Chapter 3: Manage the Database of Known UAA Sites
Chapter 4: Performance of the Prediction Model
Chapter 5: Strategies of UAA Site Prediction with RPDUAA
Chapter 6: Scope of Application and Limitation of RPDUAA
Chapter 7: Updates and New Version Maintenance of RPDUAA

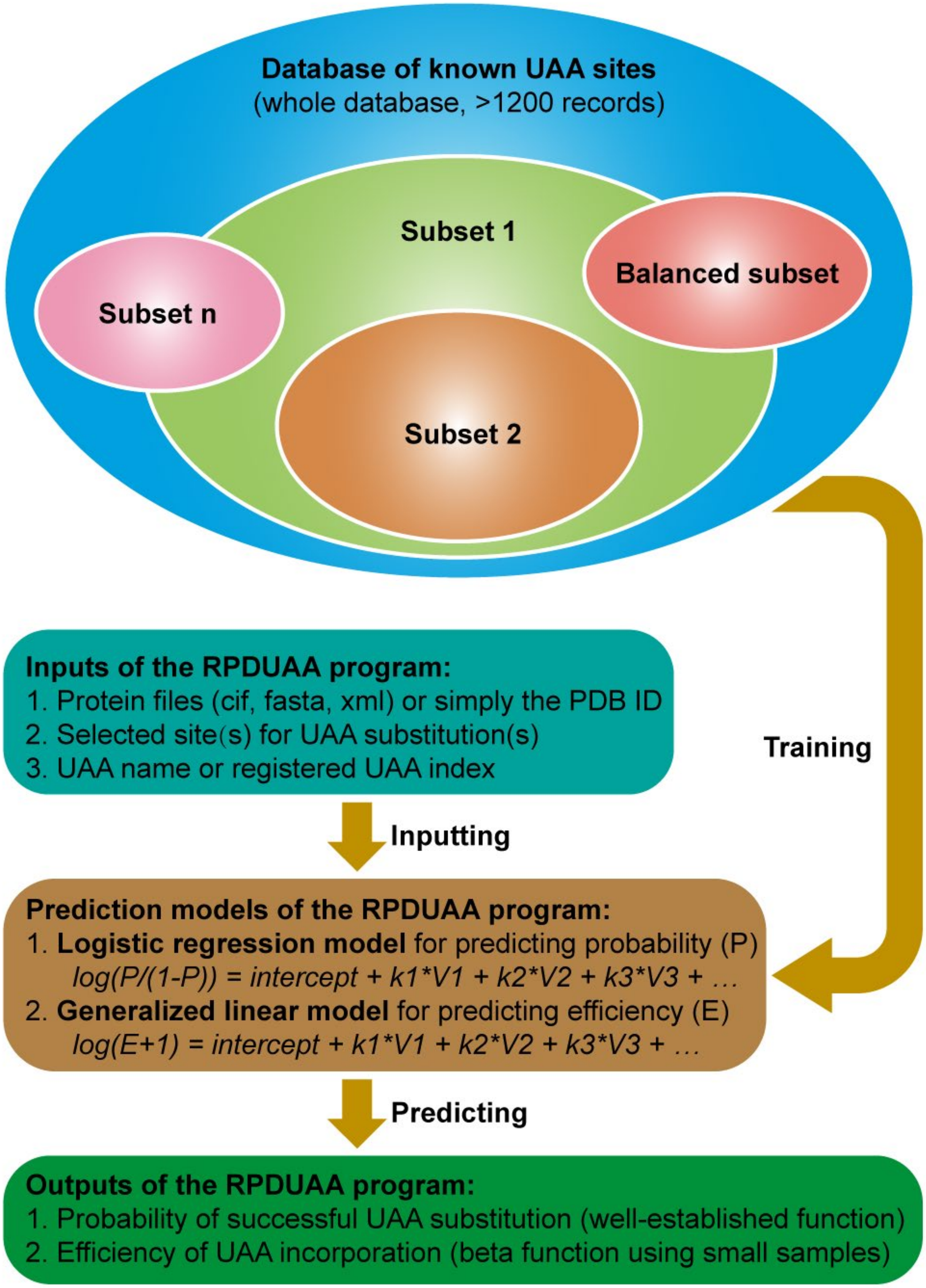

Principle of the RPDUAA program: Machine learning on UAA incorporations

Chapter 1: Quick Start for Using RPDUAA

This chapter will show the basic function of RPDUAA, using Herceptin (Protein Data Bank ID: 6mh2) as the model protein and NAEK (Nε-2-azidoethyloxycarbonyl-L-lysine) as the model UAA. Imagine you need to site-specifically incorporate NAEK into one site of the Herceptin protein through genetic code expansion, but you have no idea of which site on the protein is optimal for UAA substitution. The RPDUAA program will help you preselect high-confidence sites for successful UAA incorporation.

1.1 Install the RPDUAA program

The RPDUAA program is quite easy to install. Just unzip the RPDUAA package into a convenient folder (such as D:/RPDUAA) on a Windows-Platform computer (64-bit). The RPDUAA working folder normally contains 3 subfolders and 3 files as shown below:

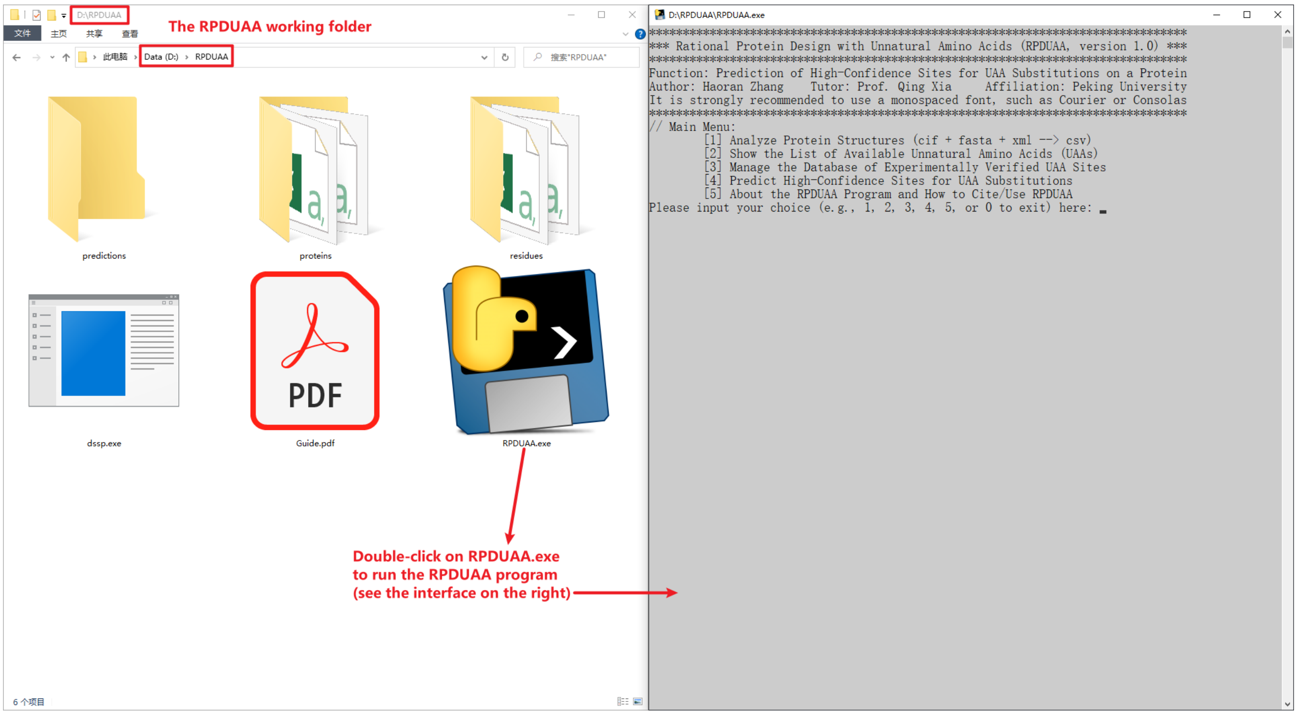

The "predictions" subfolder is used to store the prediction results by RPDUAA.
The "proteins" subfolder is used to store protein sequences and structures.
The "residues" subfolder is used to store residue information and UAA database.
The "dssp.exe" is a program provided by https://swift.cmbi.umcn.nl/gv/dssp/
The "Guide.pdf" is a detailed guidebook for the RPDUAA program.
The "RPDUAA.exe" is the main executable program. Double-click on "RPDUAA.exe" will initiate a terminal-like interface as shown above (right part). Users could analyze proteins or perform UAA substitution predictions by inputting corresponding orders and pressing the "Enter" button on the keyboard.

1.2 Prepare protein sequences and structures

If users are to analyze a new protein that does not exist in the "proteins" folder, they need to download the protein sequences and structures and prepare 3 files in

cif, fasta and xml formats, respectively.
The cif file contains the protein structure information (PDBx/mmCIF format).
The fasta file contains the protein sequence that matches the structure above.
The xml file contains the multiple-sequence-alignment information from BLAST.
The RPDUAA program can **automatically download** the above 3 files, just by adding a single **underscore prefix “_”** before the PDB ID (e.g., **_6mh2**, skip to **Section 1.3**).

1.2.1 Download the cif and fasta files manually

Visit the Protein Data Bank (PDB) website (https://www.rcsb.org/). Search “6mh2” or any other protein. Download its FASTA sequence and PDBx/mmCIF format structure. You will get two files in cif and fasta formats, respectively. Rename them with the 4-letter PDB ID (lowercase recommended).

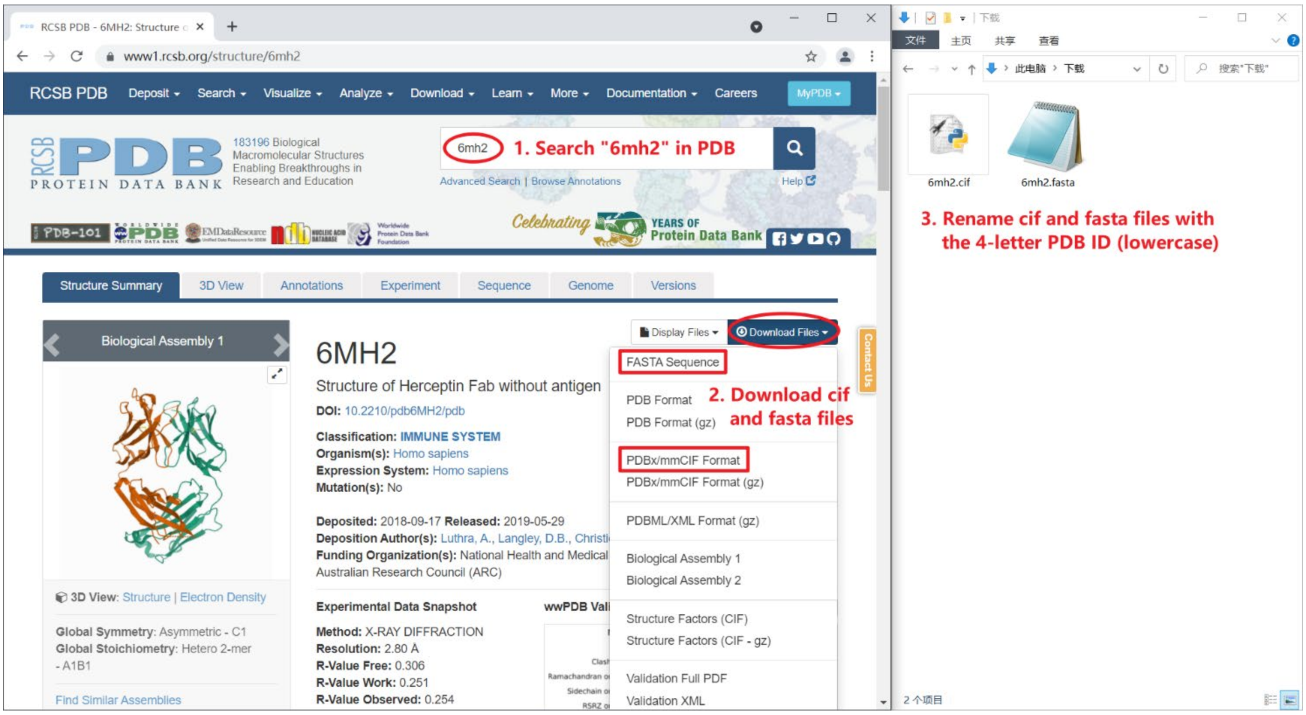

Alternatively, if your protein does not have a coordinate in PDB, you can use in silico predicted structures instead. Many platforms support predicting the protein structures from its sequences, such as RoseTTAFold and AlphaFold2. For this purpose, you should prepare a fasta file like those downloaded from PDB, and copy the fasta sequence to a new job in RoseTTAFold (https://robetta.bakerlab.org/) or AlphaFold2. After the job is done, you will get the predicted structure of the protein in pdb format. By the way, both cif and pdb format are supported by RPDUAA, although cif is more recommended. You can transform pdb to cif using the PyMOL software.

1.2.2 Download the xml files manually

Visit the NCBI BLAST website (https://blast.ncbi.nlm.nih.gov/Blast.cgi) and choose the Protein BLAST (blastp).
Upload the aforementioned FASTA sequence file (6mh2.fasta) and click on the “BLAST” button at the end of page (see below). This will start a job of searching similar sequences in the Non-Redundant Database, which may take a minute or so.

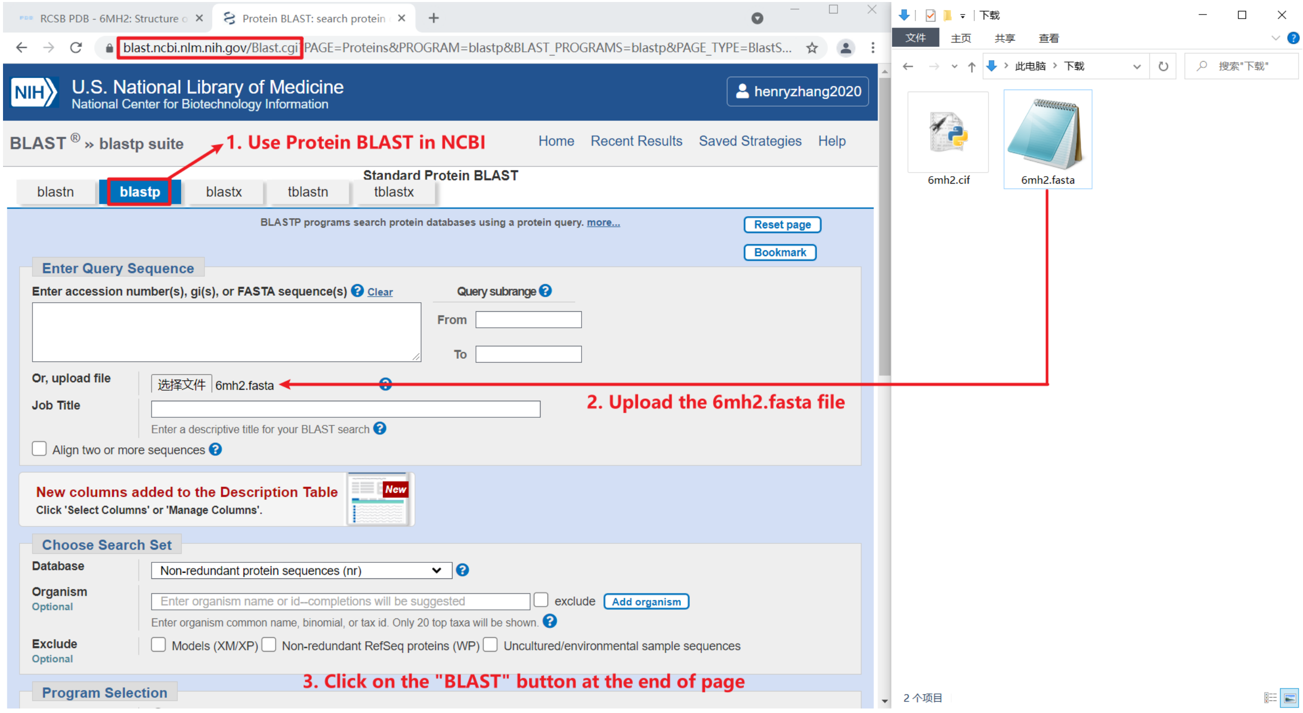

After finishing the job, the BLASÉ results of the queried protein will be reported on the webpage. Click on the “Download All” drop-down list (see below) and choose the “XML” format. You will get a xml file that contains the multiple-sequence-alignment information of the queried protein (6mh2). Rename the xml file with the 4-letter PDB ID (lowercase, or 6mh2.xml).

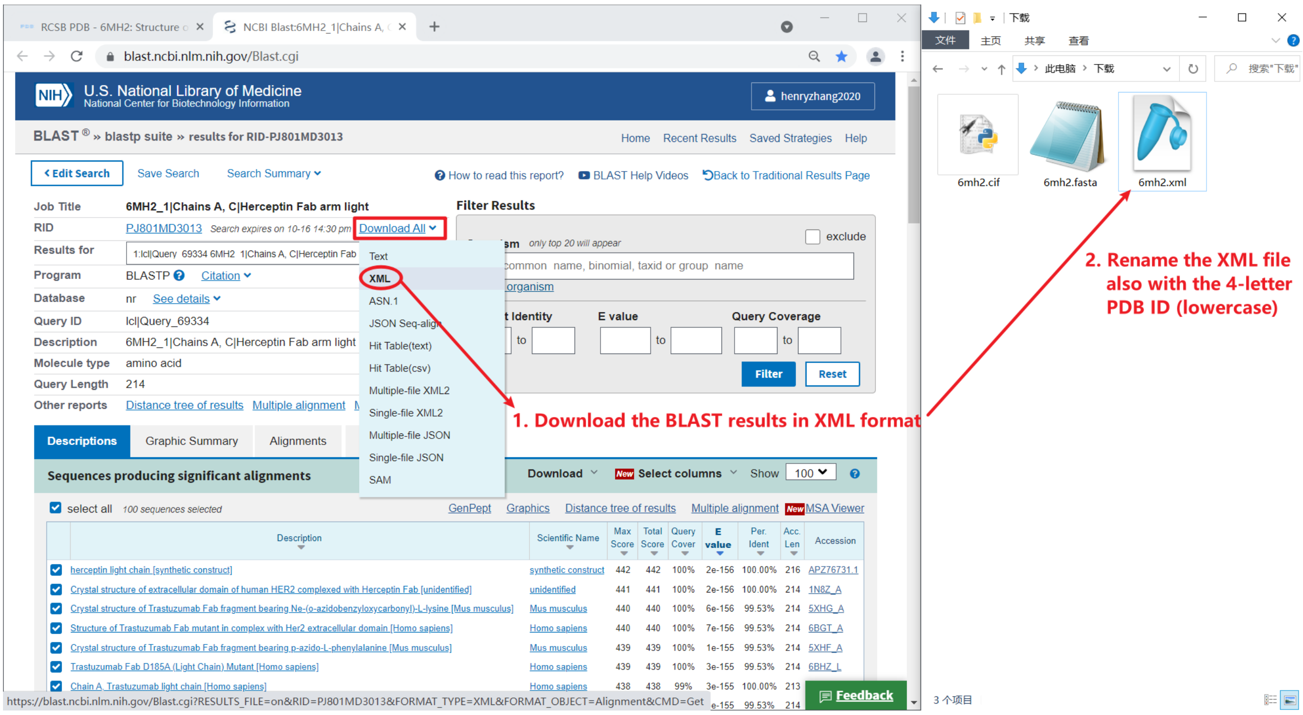

1.2.3 Copy the 3 files into the “proteins” subfolder manually

Now you have prepared 3 files (cif, fasta, and xml) that are uniformly named with the 4-letter PDB ID (lowercase). The next step is copy them into the “proteins”

subfolder (see below), so that the RPDUAA program can call and use them.

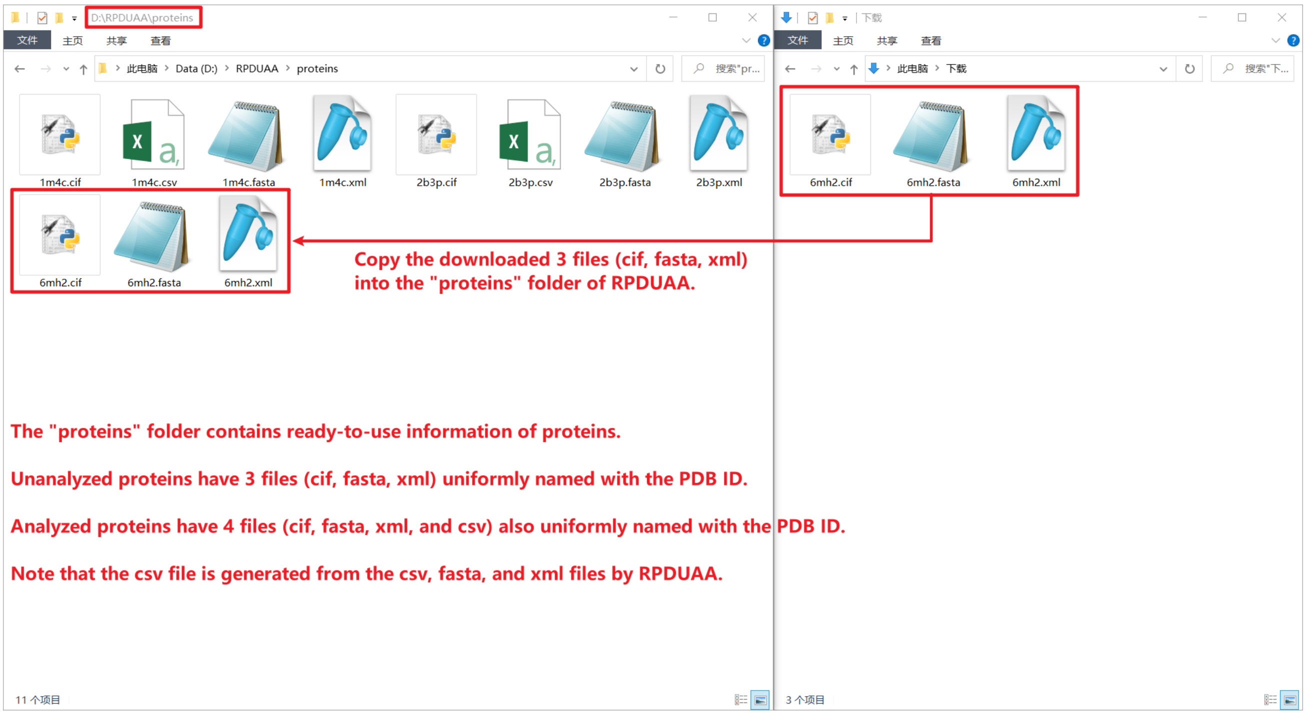

The “proteins” subfolder of RPDUAA is designed to store ready-to-use information of proteins. Each protein here has 3-4 files uniformly named with its PDB ID. For unanalyzed new proteins, 3 files (cif, fasta, and xml) are prepared. For analyzed proteins, 4 files (cif, fasta, xml, and csv) exist, since a 4th csv file will be generated by RPDUAA from the aforementioned 3 files.

1.3 Analyze the protein by RPDUAA

Launch the “RPDUAA.exe” as described in Section 1.1. Choose Task [1] in the Main Menu by inputting “1” and pressing the “Enter” button on the keyboard.

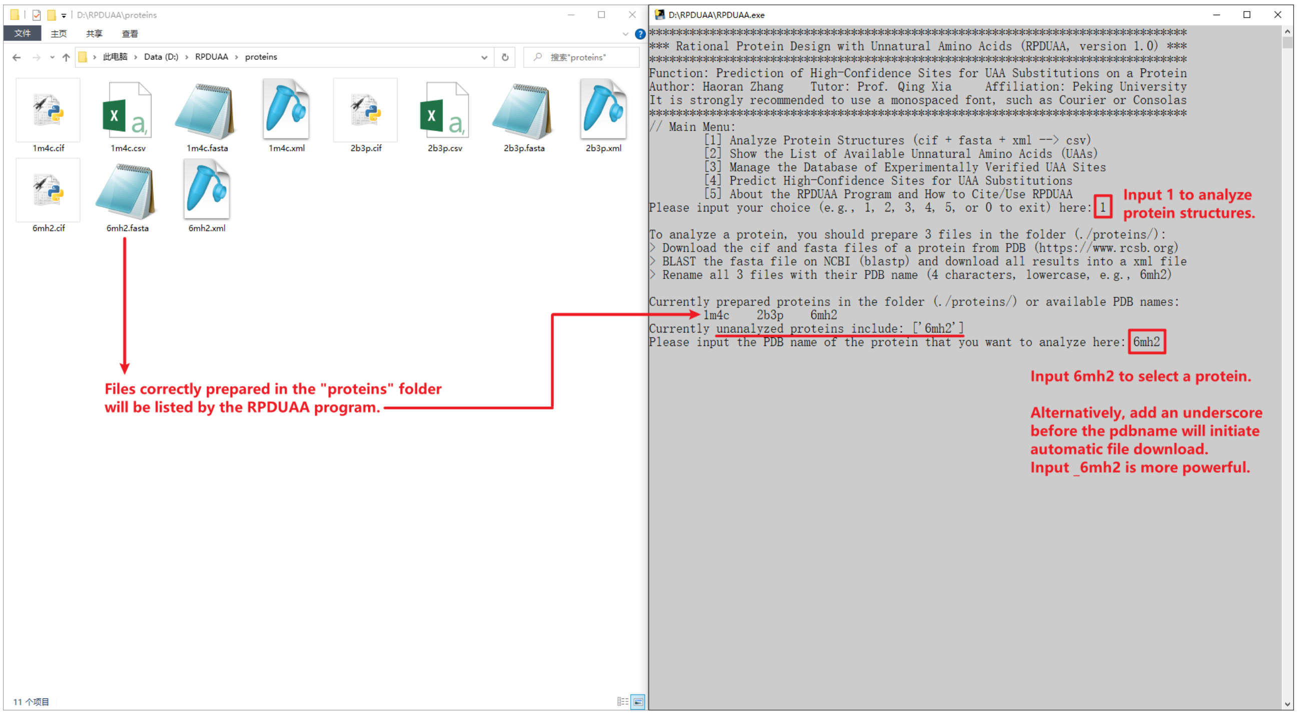

The RPDUAA program will give some hints on how to prepare 3 files and show a list of prepared proteins for you to choose. The unanalyzed protein “6mh2” is specially pointed out in the line below the list. You can type in **“6mh2”** and press “Enter” to start analyzing the Herceptin protein “6mh2”. **Alternatively, type in “_6mh2”.**

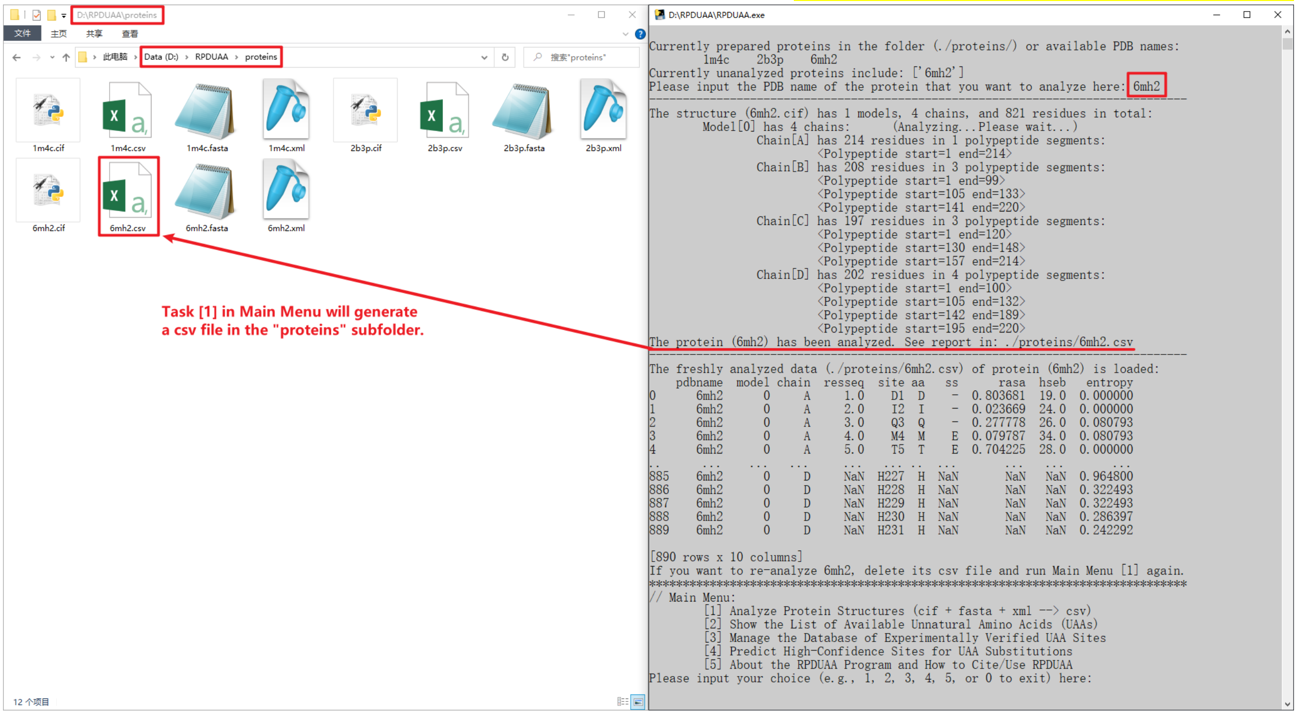

The analyzing step may take seconds to minutes depending on the structure size and computer performance. After finishing analyzing, a brief report will be given in the RPDUAA program, and a csv file (6mh2.csv) also renamed with the 4-letter PDB ID will be generated in the “proteins” subfolder. The csv file integrates the information from other 3 files (cif, fasta, and xml) in a ready-to-use manner and is necessary for the next prediction or database maintenance steps.

1.4 Predict UAA substitutions by RPDUAA

In the Main Menu of RPDUAA, choose Task [4] to predict high-confidence sites for UAA substitutions by inputting “4” and pressing “Enter”.
Then choose the recommended Subset 1 (the whole database) by inputting “1” and pressing “Enter”. This decides what observations we base our prediction on.
A figure will emerge that shows the performance (PCA, scatterplot, and ROC curve) of machine learning and the prediction model. Close that figure to continue.

The RPDUAA program provides three strategies of prediction or optimization:
(1) For a given protein and a given UAA, find the optimal substitution sites.
(2) For a given protein and given substitution site(s), find the optimal UAAs.
(3) Full probability matrix for all UAAs scanning all sites of a given protein.

Since our goal in this example is to find the optimal substitution sites of NAEK (the UAA) on the Herceptin protein (6mh2), We choose <u>Strategy 1</u> by inputting “1” and pressing “Enter”.

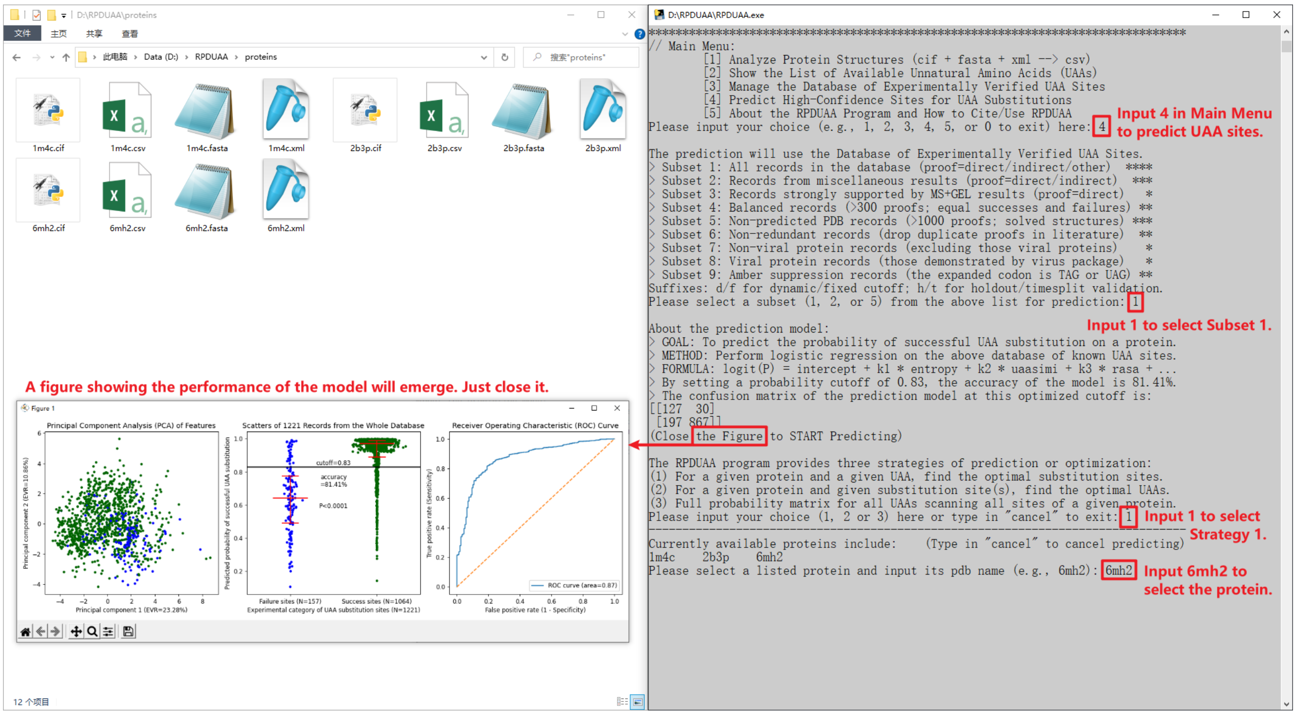

After choosing Strategy 1, the RPDUAA program asks you to select a protein for further prediction. Just input “6mh2” and press “Enter”. RPDUAA will call the csv report (6mh2.csv) in the “proteins” subfolder for upcoming predictions.

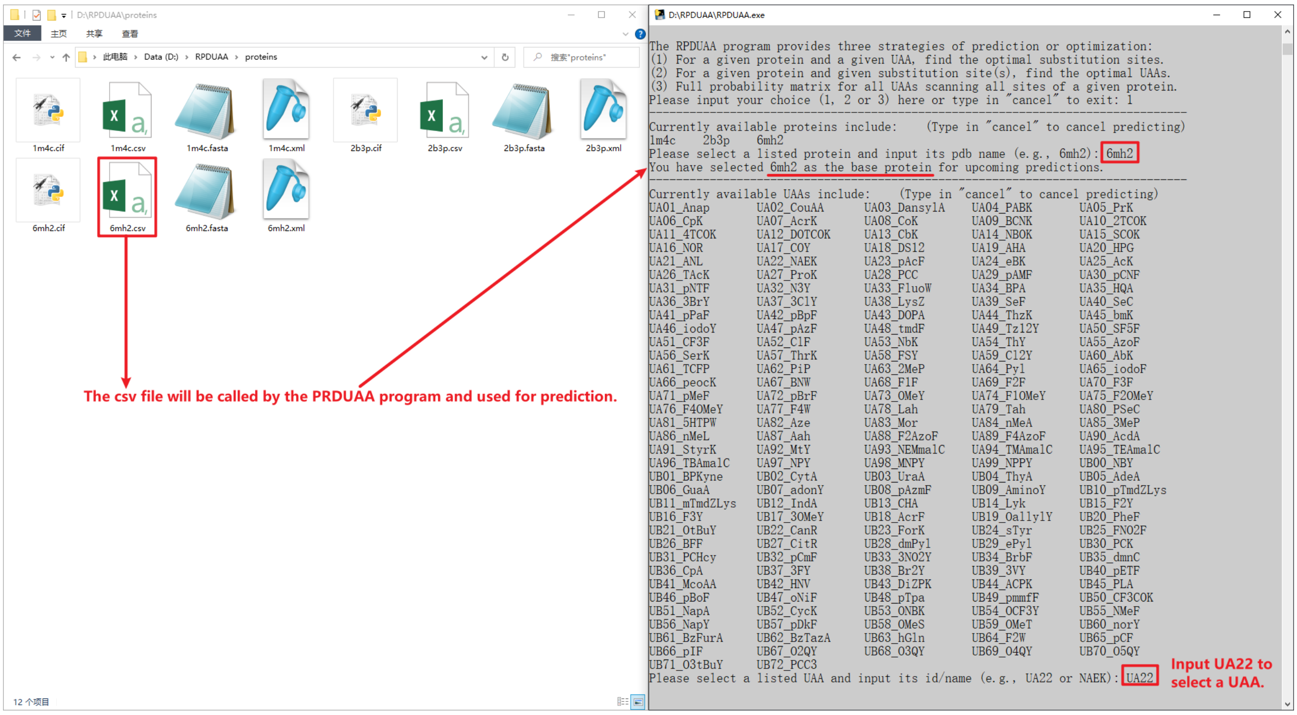

After selecting the protein “6mh2”, the RPDUAA program gives a list of available UAAs and asks you to select one. Since NAEK corresponds to “UA22_NAEK” in the list, we can choose NAEK by inputting “UA22” or “NAEK” and pressing “Enter”.

Next the RPDUAA program asks you to set the codon for UAA. Input “1” to set a TAG or amber codon. Then comes the proof level. Input “1” to set a looser definition. Finally, the RPDUAA program will return a brief report of the prediction, and

generate a csv file in the “predictions” subfolder containing the full prediction details. The csv file (“pred000n.csv”) is sequentially named, like “pred0000.csv”, “pred0001.csv” and so on. The last column of the csv file denotes the probability of successful UAA substitution in a descending order. Users can thus choose high-confidence UAA substitution sites according to the predicted probability.

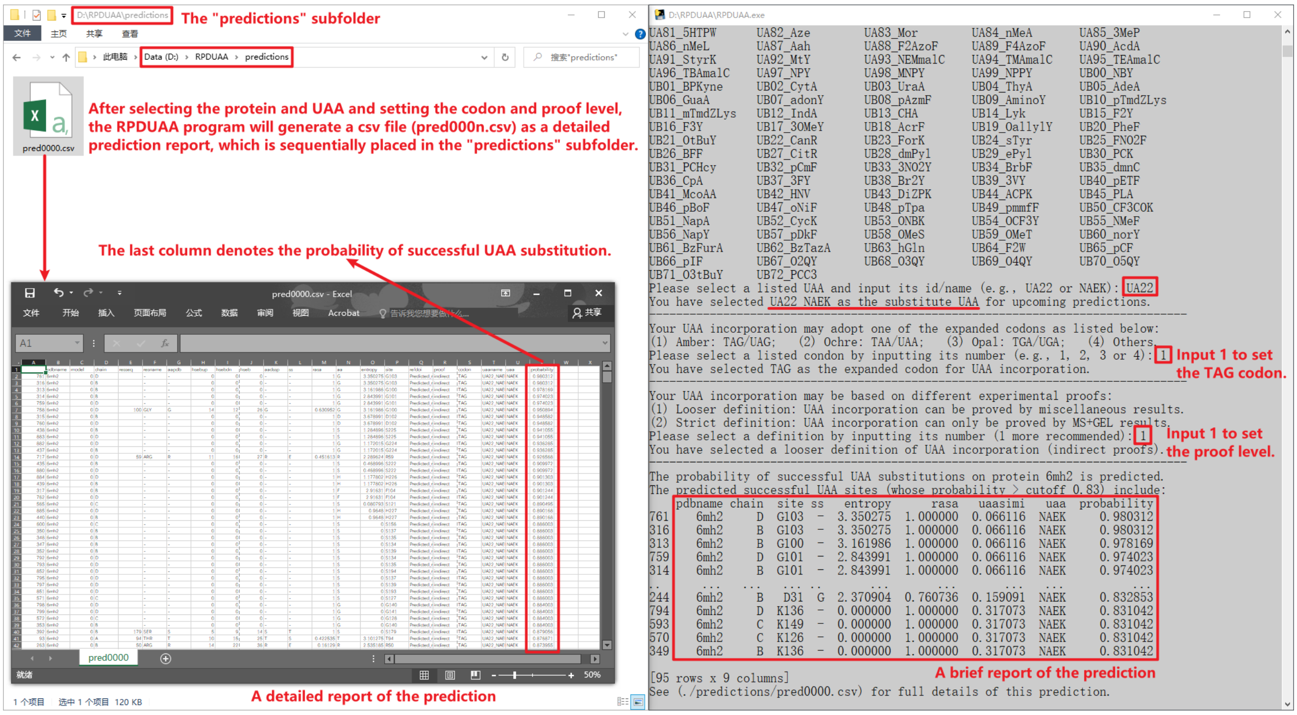

The prediction is realized by machine learning on >1000 reported UAA substitution records in the database. The receiver operating characteristic (ROC) curve showed an area of 0.87, which is fairly well for UAA substitution prediction. By setting a probability cutoff of 0.83, the prediction model demonstrated an accuracy of 81.41% and could well discriminate experimental success sites over failure sites. Users can refer to the predicted probability in the csv report (pred000n.csv) and customize the cutoff if needed. For example, treat the predicted probability larger than the cutoff as highly confident and select those sites for further studies.

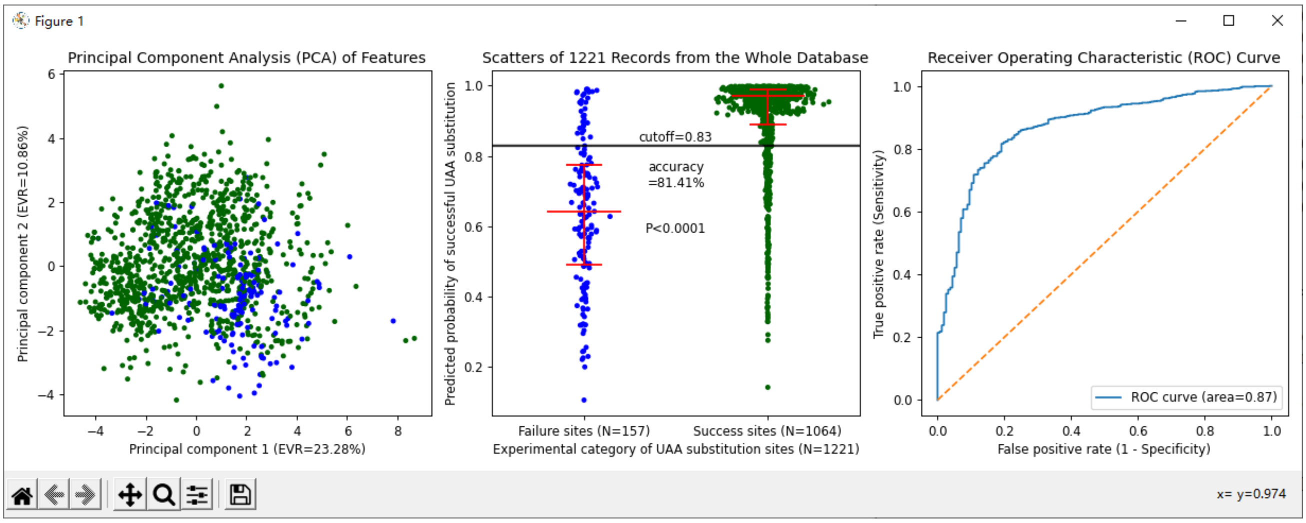

Now you have predicted NAEK substitutions on 6mh2 using Strategy 1. Perhaps, you may further want to predict substitutions with all kinds of UAAs on 6mh2, which can be done using Strategy 3 in RPDUAA. Just initiate another prediction task.

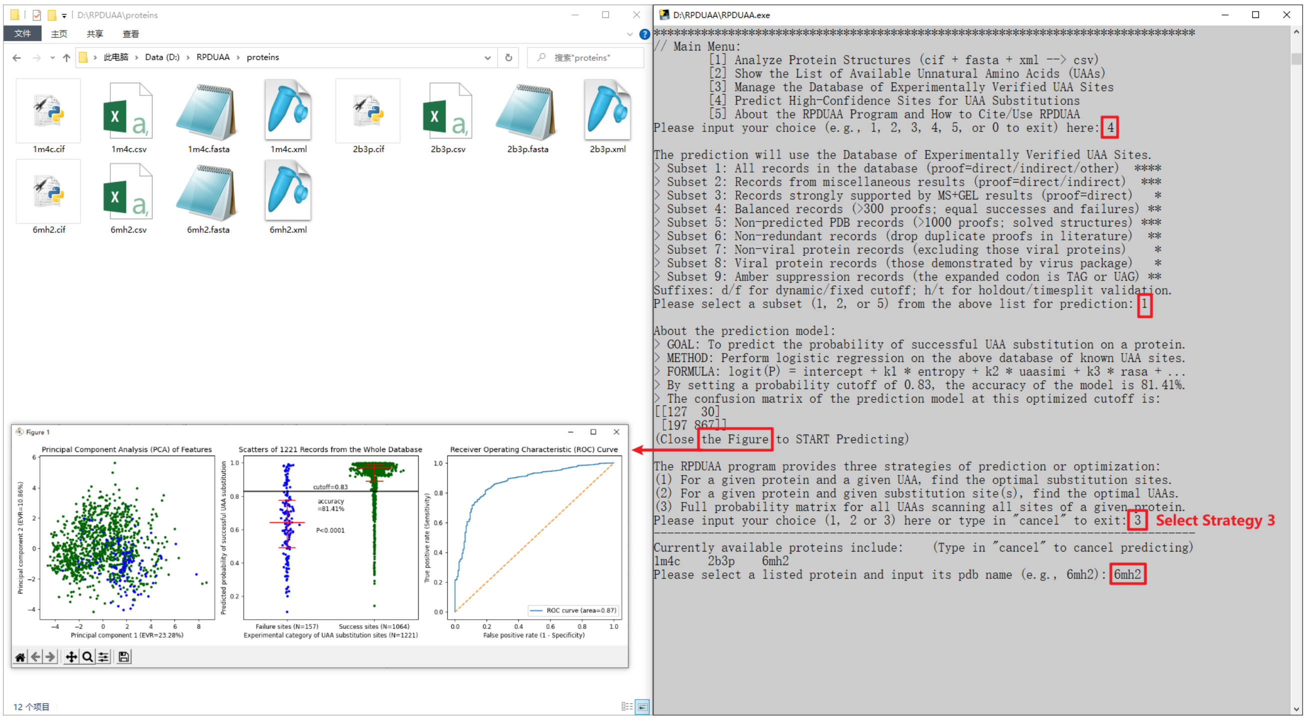

After setting the codon (input 1) and proof level (input 1), a full heatmap showing the probability for every site substituted by every registered UAA will emerge. And a detailed csv report will be generated in the "predictions" subfolder.

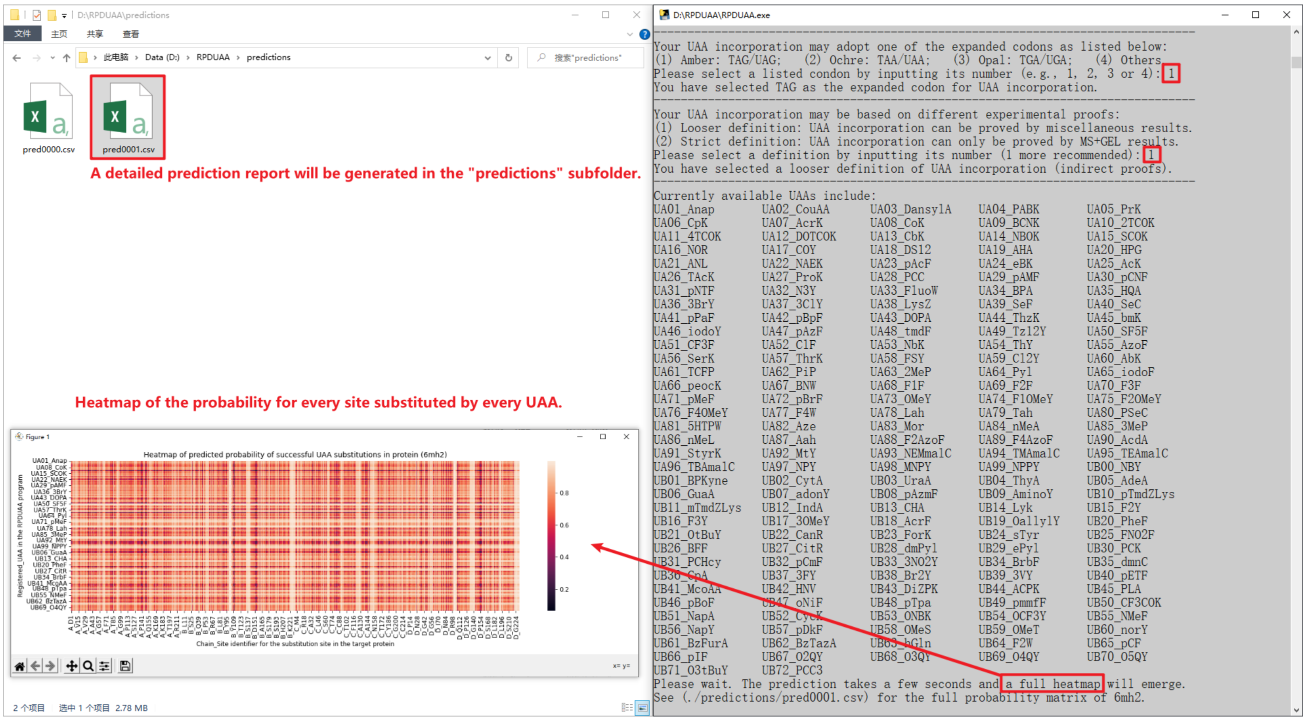

In summary, the RPDUAA program is a reliable and useful tool for predicting and preselecting UAA substitution sites on proteins.

Chapter 2: List of Available UAAs and Their Formulae

This chapter will show the list of available UAAs along with their formula. By choosing Task [2] in the Main Menu of RPDUAA, you can get a list of indexed UAA names (see below). These index UAA names come from two csv files in the "residues" subfolder of RPDUAA, namely "mole_properties.csv" and "similarity_matrix.csv". The RPDUAA program uses information in the two csv files to calculate the difference between UAA residues and canonical residues for a UAA substitution record.

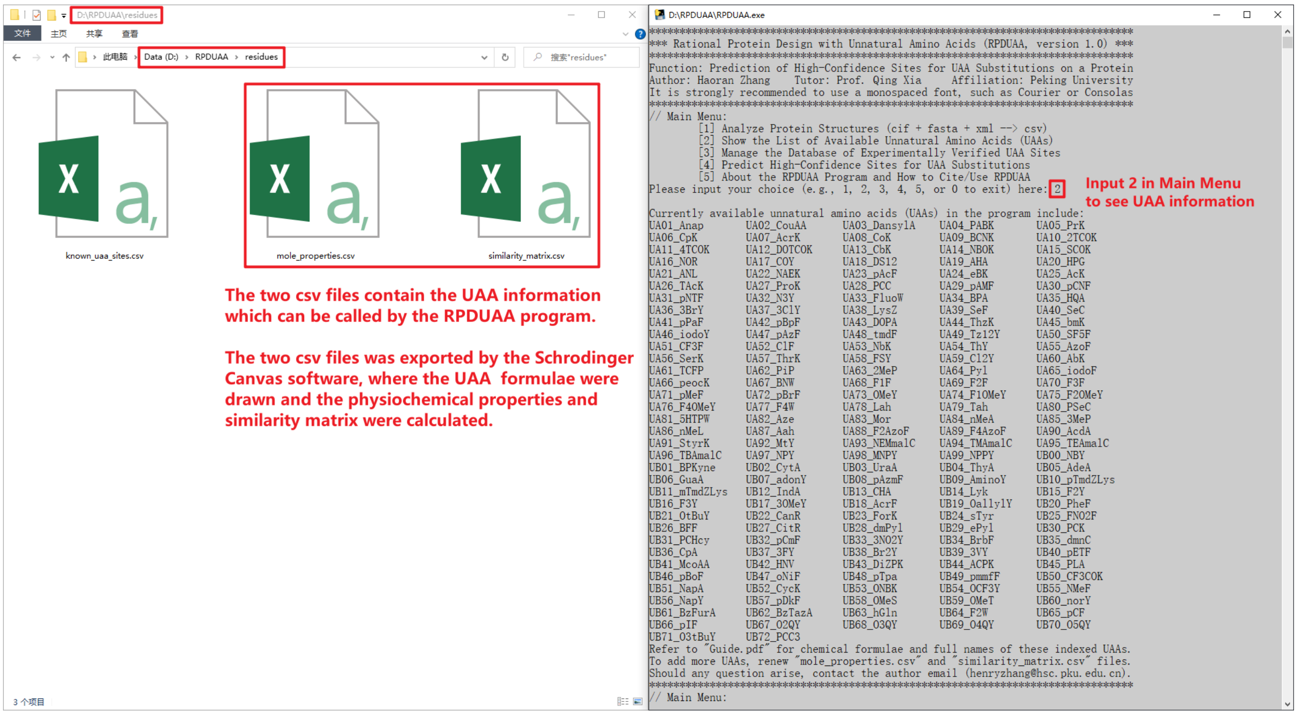

However, the RPDUAA program (version 1.0) does not support inputting UAA formulae directly. The UAA formulae and database are built in the Schrodinger Canvas 3.5 software instead. After drawing all UAA formulae in Canvas and renaming UAAs with indexed prefix, the physiochemical properties and similarity matrix of UAAs were generated and exported into the aforementioned two csv files.

The RPDUAA program (version 1.0) supports more than 170 UAAs reported in literature and users can search the SMILES string of a UAA in this PDF. Should the users need to add more UAAs, they may laboriously use the Schrodinger Canvas software to build the UAA formulae and database, export two csv files ("mole_properties.csv" and "similarity_matrix.csv") likewise, and finally renew the two csv files into the "residues" subfolder. Alternatively, users may just contact the author email (henryzhang@hsc.pku.edu.cn) for an updated version of the two csv files.

The nomenclature of UAAs takes the form of "UXnn_AbbR", where "U" denotes unnatural, "X" denotes a series, "nn" denotes two numbers, "AbbR" denotes the abbreviation of the UAA (no more than 10 characters, and the last character usually corresponds to a parent canonical amino acid), and the underscore "_" separates the index part and abbreviation part. Examples: "UA22_NAEK", "UA47_pAzF", and "UB64_F2W".

The following table shows the index name, formula, full name or alias of available UAAs in the RPDUAA program (version 1.0). You may search some keywords of typical chemical groups (for example, azido, benzoyl or coumarin) or the UAA abbreviations (for example, pAzF, NAEK, or OMeY) to find a UAA of interest in this table.

Index Name	UAA Formula	Full Name or Alias in Literature
UA01_Anap	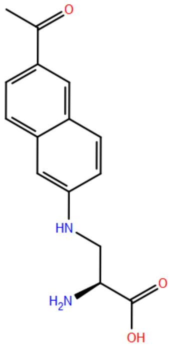	3-(6-acetylnaphthalen-2-ylamino)-2-aminopropanoic acid (Anap, ANAP)<br><br>Reference DOI:<br>10.14348/molcells.2019.0078<br>10.1021/acs.analchem.9b00445<br>10.1038/s41467-018-05339-6<br>10.7554/eLife.26355
UA02_CouAA	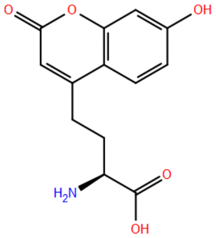	L-(7-hydroxycoumarin-4-yl) ethylglycine (7H4MC, Hco, CouA, CouAA, HCEG, 7-HCou)<br><br>Reference DOI:<br>10.14348/molcells.2019.0078<br>10.1074/jbc.RA120.016617<br>10.1016/j.bpj.2019.12.036<br>10.1016/j.bmcl.2011.08.057
UA03_DansylA	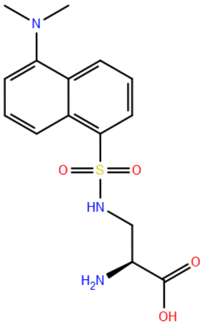	2-amino-3-(5-(dimethylamino)naphthalene-1-sulfonamide) propanoic acid (Dansylalanine, DanAla, DanA)<br><br>Reference DOI:<br>10.1002/stem.679<br>10.1002/cbic.201000436<br>10.1021/ja800894n
UA04_PABK	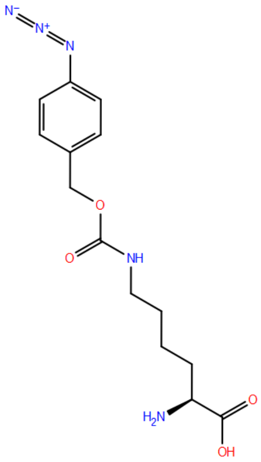	Nε-p-azidobenzyloxycarbonyl lysine (PABK)<br><br>Reference DOI:<br>10.1039/c6sc02615j

Index Name	UAA Formula	Full Name or Alias in Literature
UA05_PrK	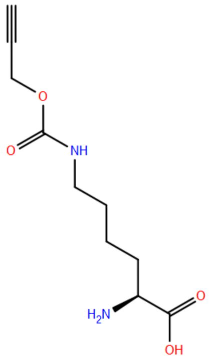	Nε-propargyl-L-lysine (PrK, ProK)<br><br>N6-[(2-propynyloxy)carbonyl]-l-lysine<br><br>Reference DOI:<br>10.3389/fbioe.2021.594429<br>10.1016/j.bmcl.2019.126876<br>10.1016/j.celrep.2020.107811<br>10.1038/s41589-020-0507-z<br>10.1039/d0ob00972e<br>10.1038/nature24659<br>10.1016/j.bmc.2017.05.003<br>10.1186/s13036-016-0031-6<br>10.1021/ja104609m
UA06_CpK	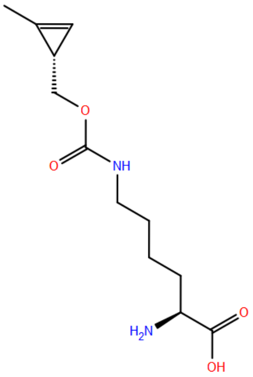	N-ε-[(2-methyl-2-cyclopropene-1-yl)-methoxy]carbonyl-L-lysine<br><br>cyclopropene-L-lysine (CpK)<br><br>Reference DOI:<br>10.14348/molcells.2019.0078<br>10.1021/ja5069728<br>10.1016/j.celrep.2020.107811
UA07_AcrK	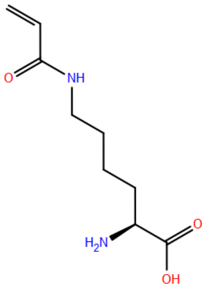	Nε-acryl-L-lysine<br>Nε-acryllysine (AcrK)<br><br>Reference DOI:<br>10.14348/molcells.2019.0078
UA08_CoK	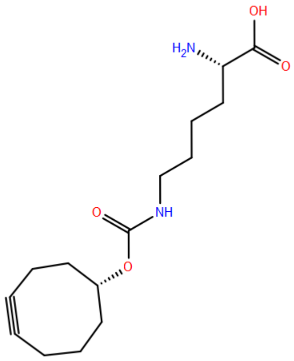	Nε--(cyclooct-2-yn-1-yloxy)carbonyl)L-lysine (CoK)<br><br>Reference DOI:<br>10.14348/molcells.2019.0078

Index Name	UAA Formula	Full Name or Alias in Literature
UA09_BCNK	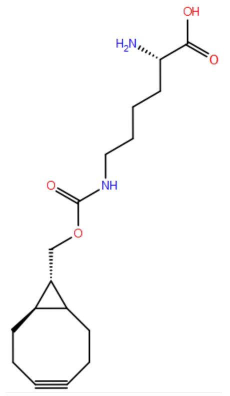	bicyclo[6.1.0]non-4-yn-9-ylmethanol lysine (BCNK)<br><br>Nε-(bicyclo[6.1.0]non-4-yn-9-yl-methoxy) carbonyl-L-lysine<br><br>bicyclononyne-lysine (BCNK)<br><br>Reference DOI:<br>10.3390/ijms20102577<br>10.1038/srep39841<br>10.1016/j.bmc.2016.09.035<br>10.1021/ja512838z
UA10_2TCOK	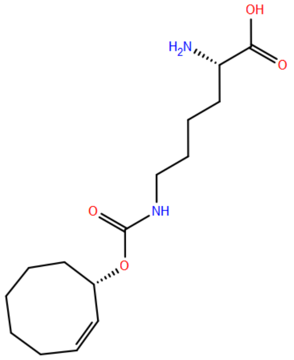	trans-cyclooct-2-ene-L-lysine (TCO*K, TCO*A, 2'-TCOK)<br><br>Reference DOI:<br>10.14348/molcells.2019.0078<br>10.1016/j.celrep.2020.107811<br>10.1016/j.bbrep.2018.10.011
UA11_4TCOK	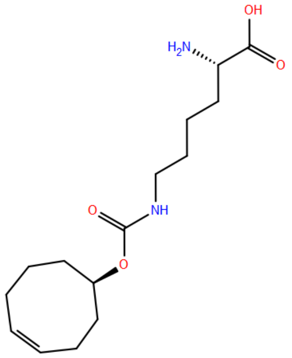	trans-cyclooct-4-ene lysine (TCOK, 4’-TCOK)<br><br>Reference DOI:<br>10.14348/molcells.2019.0078<br>10.1021/ja512838z
UA12_DOTCOK	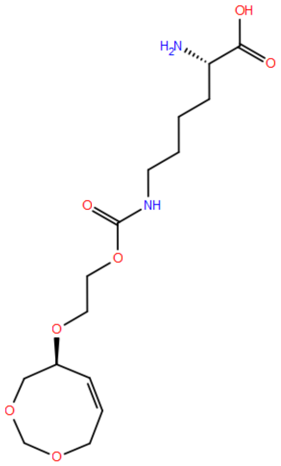	dioxo-TCO lysine (DOTCOK)<br><br>dioxo-trans-cyclooctene-lysine<br><br>Reference DOI:<br>10.14348/molcells.2019.0078

Index Name	UAA Formula	Full Name or Alias in Literature
UA13_CbK	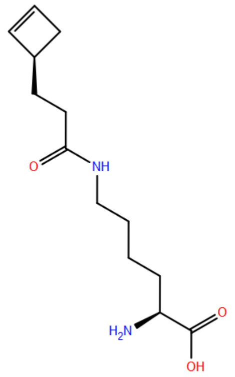	3-(2-cyclobutene-1-yl)propanoic acid -L-lysine (CbK)<br><br>Reference DOI:<br>10.14348/molcells.2019.0078
UA14_NBOK	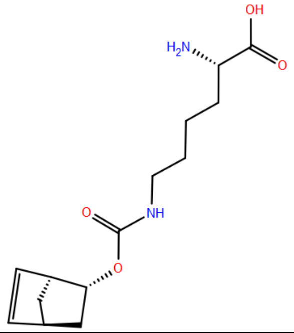	Nε-5-norbornene-2-yloxycarbonyl-L-lysine (NBOK)<br><br>Reference DOI:<br>10.14348/molcells.2019.0078
UA15_SCOK	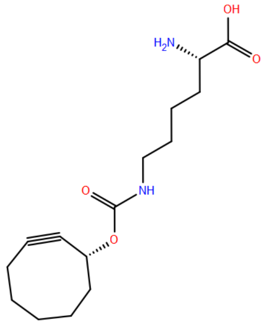	cyclooctyne lysine (SCOK)<br><br>Reference DOI:<br>10.14348/molcells.2019.0078
UA16_NOR	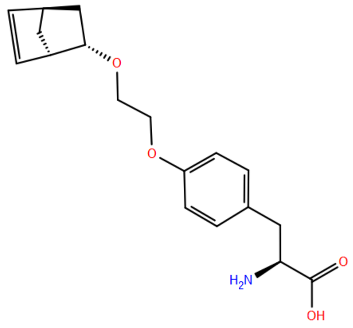	5-norbornen-2-ol tyrosine (NOR)<br><br>Reference DOI:<br>10.14348/molcells.2019.0078
UA17_COY	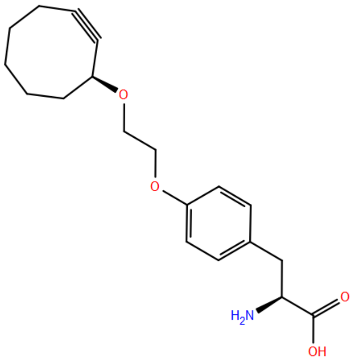	cyclooct-2-ynol tyrosine (COY)<br><br>Reference DOI:<br>10.14348/molcells.2019.0078

Index Name	UAA Formula	Full Name or Alias in Literature
UA18_DS12	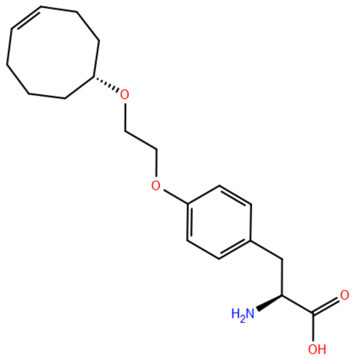	(E)-2-(cyclooct-4-en-1-yloxyl)ethanol tyrosine (DS1/2)<br><br>Reference DOI:<br>10.14348/molcells.2019.0078
UA19_AHA	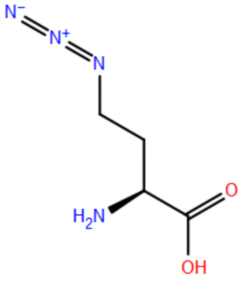	L-azidohomoalanine (AHA, Aha)<br><br>Reference DOI:<br>10.14348/molcells.2019.0078<br>10.1021/jacs.9b06473<br>10.1021/jp5008279<br>10.1002/cbic.200700379<br>10.1021/bc700390r
UA20_HPG	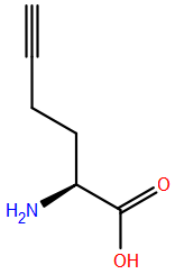	L-homopropargylglycine (HPG)<br><br>Reference DOI:<br>10.14348/molcells.2019.0078<br>10.1074/jbc.M117.791723<br>10.1002/cbic.200700379<br>10.1021/bc700390r
UA21_ANL	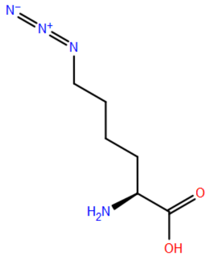	L-azidonorleucine (ANL)<br><br>Reference DOI:<br>10.14348/molcells.2019.0078
UA22_NAEK	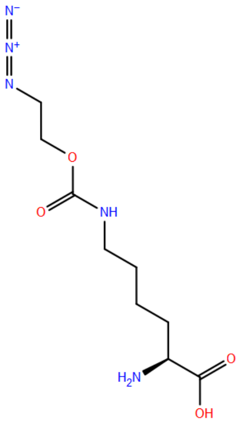	Nε-2-azidoethyloxycarbonyl-L-lysine<br>N6-(2-azidoethoxy)-carbonyl-L-lysine<br>(NAEK, AzK, NEAK, AzeoK, Alk)<br><br>Reference DOI:<br>10.1126/science.aah5869<br>10.1038/s41551-021-00774-1<br>10.1080/15476286.2021.1907055<br>10.1016/j.celrep.2020.107811<br>10.1038/s41589-020-0507-z<br>10.1186/s13036-016-0031-6<br>10.1016/j.bmc.2016.09.035

Index Name	UAA Formula	Full Name or Alias in Literature
UA23_pAcF	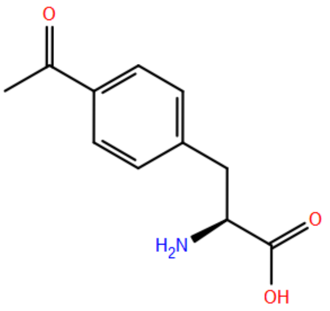	p-acetyl-L-phenylalanine<br>(pAcF, AcF, AcPhe, Apa, Acp, pApa)<br><br>Reference DOI:<br>10.1016/j.synbio.2021.07.003<br>10.1021/acssynbio.9b00076<br>10.1073/pnas.1507741112<br>10.1021/sb500195w<br>10.1093/protein/gzv048<br>10.1074/jbc.M707355200<br>10.1038/nmeth1016
UA24_eBK	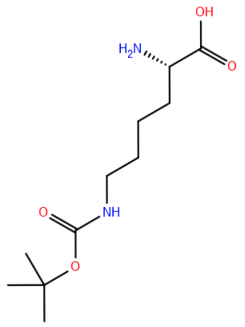	Nε-tert-Butoxycarbonyl-L-lysine<br>Nε-Boc-L-lysine (BocK, eBK, tBOCK)<br><br>Reference DOI:<br>10.1007/s00726-020-02927-z<br>10.1016/j.bmcl.2019.126876<br>10.1021/ja5069728<br>10.1002/anie.201308137
UA25_AcK	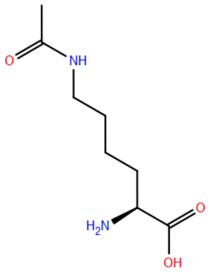	Nε-acetyl-L-lysine (ActK)<br>acetyl lysine (AcK)<br><br>Reference DOI:<br>10.1021/ja104609m<br>10.1038/nchembio.657<br>10.1007/s00726-020-02927-z
UA26_TAcK	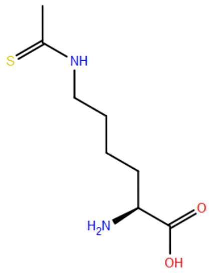	thioacetyl-L-lysine<br><br>thioacetyl lysine (TAcK)<br><br>Reference DOI:<br>10.1007/s00726-020-02927-z
UA27_ProK	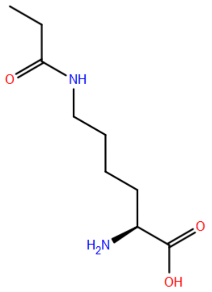	propionyl-L-lysine<br><br>propionyl lysine (PrK, ProK)<br><br>Reference DOI:<br>10.1007/s00726-020-02927-z

Index Name	UAA Formula	Full Name or Alias in Literature
UA28_PCC	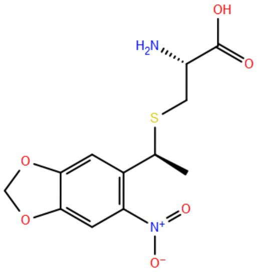	photocaged cysteine (PCC)<br><br>Reference DOI:<br>10.1007/s00726-020-02927-z
UA29_pAMF	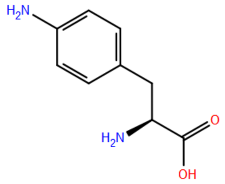	p-amino-phenylalanine (pAF)<br>para-aminophenylalanine (pAMF)<br><br>Reference DOI:<br>10.1038/nbt742<br>10.1016/j.synbio.2021.07.003<br>10.1021/acs.biochem.8b01141
UA30_pCNF	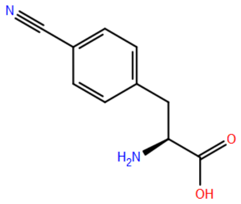	p-cyano-L-phenylalanine (pCNF)<br>4-cyano-L-phenylalanine (4-CN-Phe)<br>para-cyanophenylalanine (pCNPhe)<br><br>Reference DOI:<br>10.1021/acs.biochem.6b00898<br>10.1021/jacs.9b06580<br>10.1021/bi900426d
UA31_pNTF	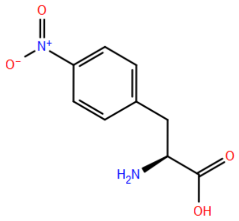	p-nitro-L-phenylalanine (pNTF)<br>4-nitro-L-phenylalanine<br>para-nitrophenylalanine<br>(pNO2Phe, pNO2-Phe, pNO2pa, pNO2F)<br><br>Reference DOI:<br>10.1111/febs.15560<br>10.1016/j.bbrc.2018.03.205<br>10.1021/ja058262u<br>10.1073/pnas.0804157105<br>10.1371/journal.pone.0009354<br>10.1107/S2053230X1801169X
UA32_N3Y	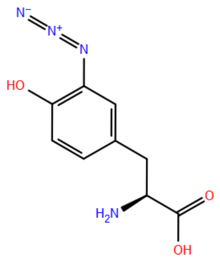	m-azido-l-tyrosine (N3Y)<br><br>3-azido-L-tyrosine<br><br>Reference DOI:<br>10.1002/anie.202016880<br>10.3390/s18082519

Index Name	UAA Formula	Full Name or Alias in Literature
UA33_FluoW	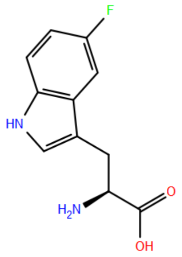	5-Fluoro-Tryptophan (5FW)<br>fluorinated tryptophan (FluoW)<br><br>Reference DOI:<br>10.1021/jp060043n<br>10.1016/j.jbc.2021.100899
UA34_BPA	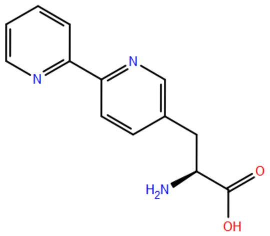	(2,20-bipyridin-5-yl) alanine<br>(2,2'-bipyridin-5-yl) alanine<br>(BipyA, BpyA, BPA)<br><br>Reference DOI:<br>10.3390/molecules26051274<br>10.1039/c7sc03477f<br>10.1016/j.jmb.2009.10.030
UA35_HQA	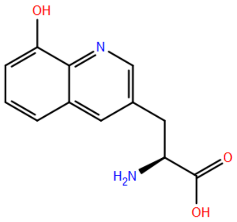	(8-hydroxy-quinolin-3-yl) alanine<br><br>2-amino-3-(8-hydroxyquinolin-3-yl) propanoic acid (HQA)<br><br>Reference DOI:<br>10.3390/molecules26051274<br>10.1016/j.jmb.2009.10.030<br>10.1021/acssynbio.9b00076<br>10.1007/s10858-014-9884-5
UA36_3BrY	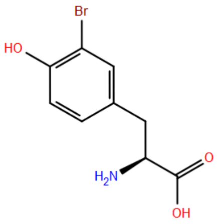	3-bromo-L-tyrosines (3BrY)<br><br>Reference DOI:<br>10.1002/cbic.202000429
UA37_3ClY	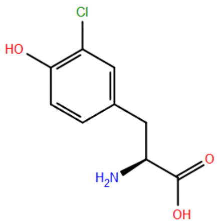	3-chloro-L-tyrosines (3ClY)<br><br>3-chlorotyrosine (ClTyr or cY)<br><br>Reference DOI:<br>10.1002/cbic.202000429<br>10.1021/acs.jpcb.9b01571<br>10.1021/ja5109936<br>10.1016/j.bmcl.2011.09.108<br>10.1038/nbt0202-177

Index Name	UAA Formula	Full Name or Alias in Literature
UA38_LysZ	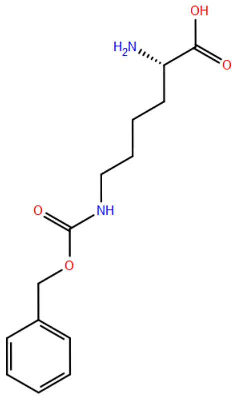	Nε-Benzyloxycarbonyl-L-lysine<br><br>CBZ lysine (LysZ, Lys(Z), ZLys)<br><br>Reference DOI:<br>10.1080/15476286.2021.1907055<br>10.1002/cbic.201300069<br>10.1038/srep36946
UA39_SeF	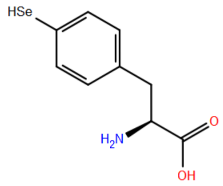	2-amino-3-(4-hydroselenophenyl) propanoic acid (SeF, SeHF, pSeHF)<br><br>selenotyrosine (SeY)<br><br>Reference DOI:<br>10.1039/d1sc02653d<br>10.1002/cbic.202000460
UA40_SeC	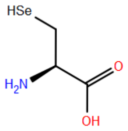	selenocysteine (SeC)<br><br>Reference DOI:<br>10.1021/acs.bioconjchem.8b00254
UA41_pPaF	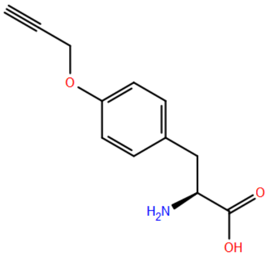	p-propargyloxy-L-phenylalanine (pPaF, pPa, pPR, Pyo, pPrF, pPpa)<br><br>Reference DOI:<br>10.1016/j.synbio.2021.07.003<br>10.1002/bit.26305<br>10.1016/j.jmb.2009.10.030<br>10.1039/b904228h<br>10.1038/nmeth1016<br>10.1002/cbic.200600347
UA42_pBpF	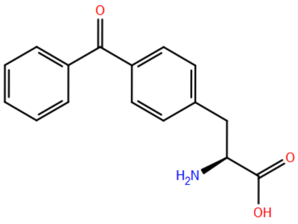	p-benzoyl-L-phenylalanine (pBpA, Bpa, BzF, Bzo, Bzp, pBpF)<br><br>Reference DOI:<br>10.1021/acschembio.8b00021<br>10.1021/acs.biochem.6b00259<br>10.1523/JNEUROSCI.3725-13.2014<br>10.1002/stem.679<br>10.1016/j.jmb.2009.10.030<br>10.1074/jbc.M707355200

Index Name	UAA Formula	Full Name or Alias in Literature
UA43_DOPA	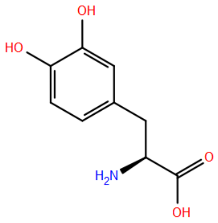	3,4-dihydroxy-L-phenylalanine<br>L-3,4-dihydroxyphenylalanine<br>(DOPA, L-DOPA)<br><br>Reference DOI:<br>10.1042/BCJ20210091<br>10.1021/bc2000066
UA44_ThzK	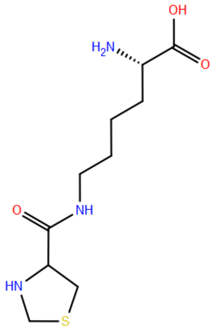	Nε-L-thiaprolyl-L-lysine<br>(ThzK, L-ThzK)<br><br>Reference DOI:<br>10.1016/j.bmcl.2019.126876
UA45_bmK	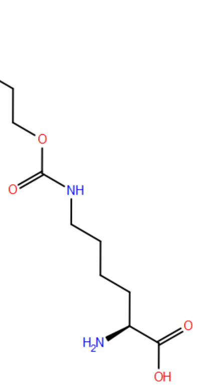	butyrylation-mimic lysine (bmK)<br><br>Reference DOI:<br>10.1038/s41589-019-0392-5
UA46_iodoY	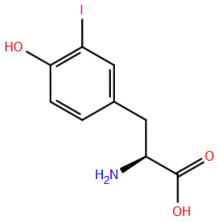	3-iodo-L-tyrosine (3-iodotyrosine)<br><br>Reference DOI:<br>10.3389/fbioe.2020.00145<br>10.1016/j.bmcl.2011.09.108<br>10.1093/nar/gkf589<br>10.1073/pnas.142220099

Index Name	UAA Formula	Full Name or Alias in Literature
UA47_pAzF	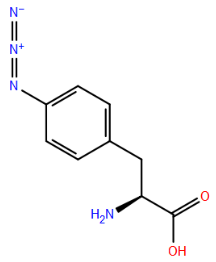	p-azido-L-phenylalanine (pAzF, AzF, 4-AzF, paF, AzPhe, pAzdF, pAzpa, pAz, Azi, Azp)<br><br>Reference DOI:<br>10.1021/acs.jcim.0c00725<br>10.1021/acssensors.9b00400<br>10.1021/acs.biochem.9b00428<br>10.1038/nature24659<br>10.1073/pnas.1318808111<br>10.1021/ac202096t<br>10.1038/nchembio.657<br>10.1021/bc9002844<br>10.1039/b904228h<br>10.1110/ps.034587.108<br>10.1038/nmeth1016
UA48_tmdF	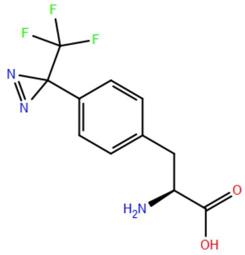	4-(trifluoromethyldiazirinyl)-phenylalanine (tmdF)<br><br>Reference DOI:<br>10.1039/c9cc09891g
UA49_Tz12Y	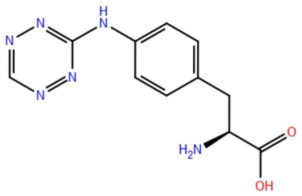	1,2,4,5-tetrazine (Tz) compound 12, a tyrosine analogue (Tz12Y)<br><br>Reference DOI:<br>10.1021/acs.bioconjchem.0c00052
UA50_SF5F	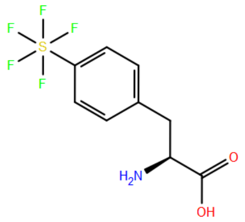	para-pentafluorosulfanyl phenylalanine (SF5Phe)<br><br>Reference DOI:<br>10.1021/jacs.0c07976
UA51_CF3F	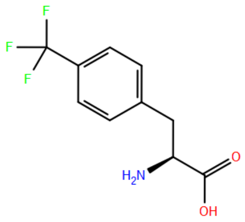	p-trifluoromethyl-L-phenylalanine (tfmF, tfm-Phe, ptfmF, mtfF)<br><br>Reference DOI:<br>10.1021/jacs.0c07976<br>10.1016/j.bbrc.2011.09.082<br>10.1021/bi901947r<br>10.1021/ja064661t<br>10.1021/jacs.1c06847

Index Name	UAA Formula	Full Name or Alias in Literature
UA52_ClF	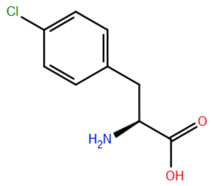	p-chloro-phenylalanine (ClPhe)<br><br>Reference DOI:<br>10.1021/jacs.0c07976
UA53_NbK	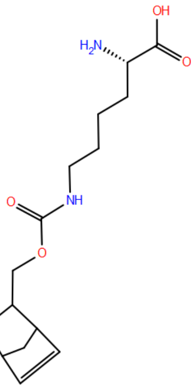	norbornene lysine (NbK)<br><br>Reference DOI:<br>10.1007/978-1-0716-0434-2_8
UA54_ThY	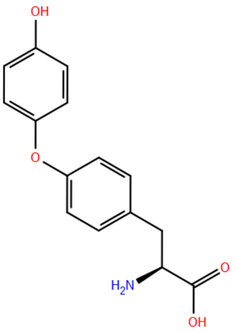	thyronine (Thy)<br><br>Reference DOI:<br>10.1016/j.bmc.2020.115665
UA55_AzoF	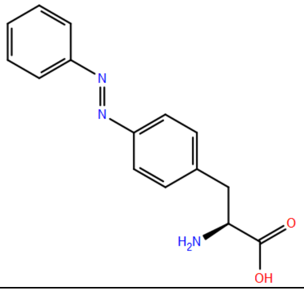	4′-azobenzene-phenylalanine (AzoPhe)<br>phenylalanine-4′-azobenzene (AzoF)<br><br>Reference DOI:<br>10.1021/acs.biochem.0c00332<br>10.1002/cbic.201800226<br>10.1016/j.bmcl.2014.12.035
UA56_SerK	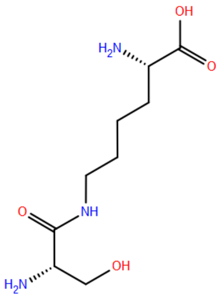	serine-lysine dipeptide (α-oxo aldehyde 1)<br><br>Reference DOI:<br>10.1039/d0ob00972e

Index Name	UAA Formula	Full Name or Alias in Literature
UA57_ThrK	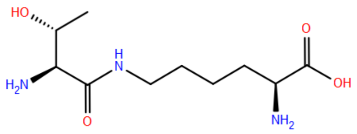	threonine-lysine dipeptide (α-oxo aldehyde 3)<br><br>Reference DOI:<br>10.1039/d0ob00972e
UA58_FSY	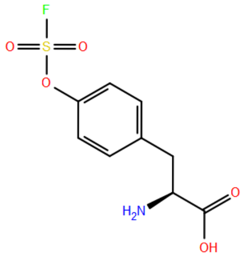	fluorosulfate-L-tyrosine (FSY)<br><br>Reference DOI:<br>10.1021/jacs.9b02611
UA59_Cl2Y	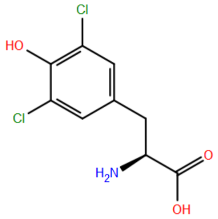	3,5-dichloro-L-tyrosines (Cl2Y)<br><br>Reference DOI:<br>10.1021/acs.jpcb.9b01571
UA60_AbK	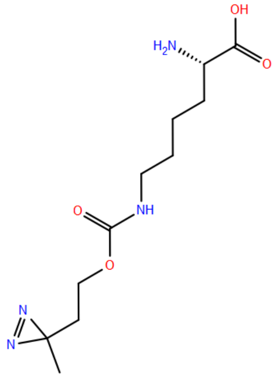	N6-((2-(3-Methyl-3H-diazirin-3-yl) ethoxy]carbonyl)-L-lysine<br><br>diazirine-lysine (AbK)<br><br>Reference DOI:<br>10.3390/ijms20102577<br>10.1021/ja104609m
UA61_TCFP	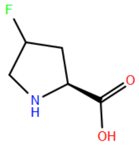	trans/cis-4-fluoroproline (F-Pro)<br><br>Reference DOI:<br>10.1021/acschemneuro.9b00315<br>10.1021/acs.biochem.8b00379
UA62_PiP	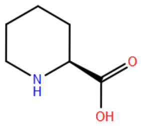	pipecolic acid (Pip)<br><br>Reference DOI:<br>10.1021/acschemneuro.9b00315<br>10.1021/acs.biochem.8b00379
UA63_2MeP	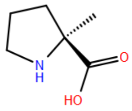	2-methylproline (2-Me-Pro)<br><br>Reference DOI:<br>10.1021/acschemneuro.9b00315<br>10.1021/acs.biochem.8b00379

Index Name	UAA Formula	Full Name or Alias in Literature
UA64_Pyl	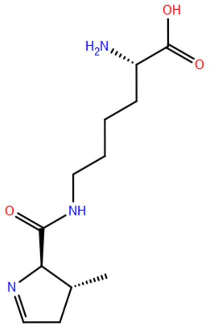	pyrrolysine (Pyl)<br><br>Reference DOI:<br>10.1002/chem.201500971
UA65_iodoF	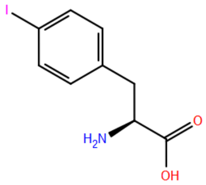	p-iodo-L-phenylalanine (iodoF, IF, pIPhe, pIodF, pIF, pIpa, iodoPhe)<br><br>Reference DOI:<br>10.1021/acssynbio.5b00209<br>10.1073/pnas.1507741112<br>10.1039/c0cc04970k<br>10.1038/nchembio.657<br>10.1016/j.jmb.2009.10.030<br>10.1371/journal.pone.0009354<br>10.1038/nbt1013
UA66_peocK	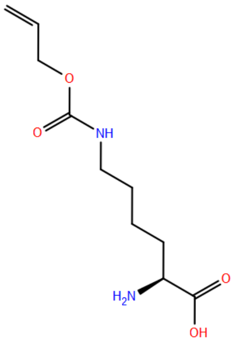	N6-[(2-propenyloxy)carbonyl]-L-lysine (UAA 10, Aloc-lysine)<br><br>Reference DOI:<br>10.1021/acssynbio.9b00076<br>10.1002/cbic.201300069
UA67_BNW	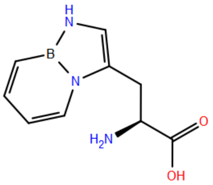	BN-tryptophan 8, a boron and nitrogen containing unnatural analogue of tryptophan<br><br>Reference DOI:<br>10.1039/c8sc05167d
UA68_F1F	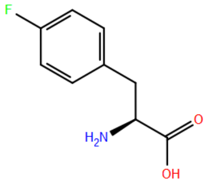	4-fluoro-L-phenylanaline (4-F-Phe)<br>p-fluoro-L-phenylanaline (F1-Phe)<br>fluorinated Phe derivatives (F1F)<br><br>Reference DOI:<br>10.1021/jacs.9b06580<br>10.1523/JNEUROSCI.2540-08.2008

Index Name	UAA Formula	Full Name or Alias in Literature
UA69_F2F	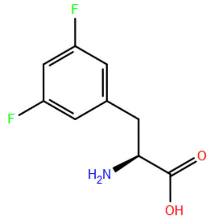	3,5-difluoro-L-phenylanaline<br>(3,5-F2-Phe)<br>fluorinated Phe derivatives (F2F)<br><br>Reference DOI:<br>10.1021/jacs.9b06580<br>10.1523/JNEUROSCI.2540-08.2008
UA70_F3F	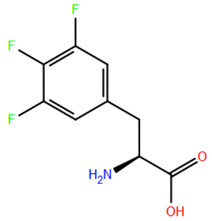	3,4,5-trifluoro-L-phenylanaline<br>(3,4,5-F3-Phe)<br>fluorinated Phe derivatives (F3F)<br><br>Reference DOI:<br>10.1021/jacs.9b06580<br>10.1523/JNEUROSCI.2540-08.2008
UA71_pMeF	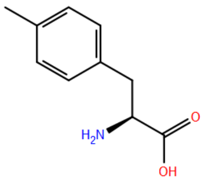	p-methyl-L-phenylanaline<br>4-methyl-L-phenylanaline<br>(4-Me-Phe, p-Me-Phe, pMeF)<br><br>Reference DOI:<br>10.1021/jacs.9b06580<br>10.1523/JNEUROSCI.2540-08.2008
UA72_pBrF	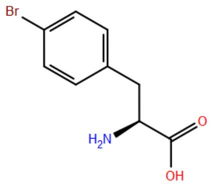	p-bromo-L-phenylanaline<br>4-bromo-L-phenylanaline<br>(4-Br-Phe, p-Br-Phe, BrF, pBrF)<br><br>Reference DOI:<br>10.1021/jacs.9b06580<br>10.1073/pnas.1507741112
UA73_OMeY	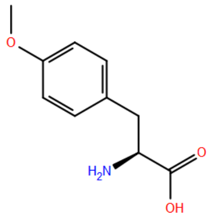	O-methyl-L-tyrosine (OMeY, Ome)<br>p-O-methyltyrosine (OMe-Tyr, pOMeY)<br>p-methoxy-L-phenylalanine (OMePhe,<br>4-O-Me-Phe, pMpa)<br><br>Reference DOI:<br>10.1021/jacs.9b06580<br>10.1002/anie.201308137<br>10.1073/pnas.1309584110<br>10.1021/cb200057a<br>10.1039/b904228h<br>10.1021/ja801602q<br>10.1038/nmeth1016<br>10.1073/pnas.0401517101<br>10.1126/science.1060077

Index Name	UAA Formula	Full Name or Alias in Literature
UA74_F1OMeY	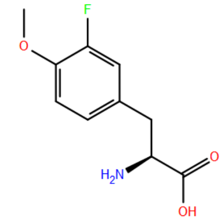	3-fluoro-O-methyl-L-tyrosine (F1-OMe-Tyr)<br><br>Reference DOI:<br>10.1021/jacs.9b06580
UA75_F2OMeY	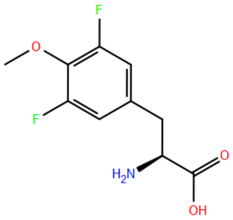	3,5-difluoro-O-methyl-L-tyrosine (F2-OMe-Tyr)<br><br>Reference DOI:<br>10.1021/jacs.9b06580
UA76_F4OMeY	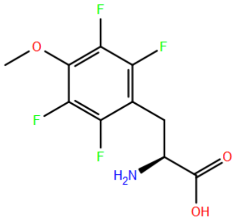	2,3,5,6-tetrafluoro-O-methyl-L-tyrosine (F4-OMe-Tyr)<br><br>Reference DOI:<br>10.1021/jacs.9b06580
UA77_F4W	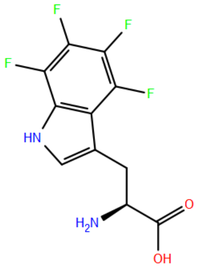	4,5,6,7-tetrafluoro-Tryptophan (4,5,6,7-F4 Trp) (F4-Trp) (F4W)<br><br>Reference DOI:<br>10.1021/jacs.9b06580<br>10.1074/jbc.M114.631960<br>10.1021/acschemneuro.5b00298
UA78_Lah	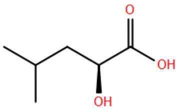	α-hydroxy acid of Leu (Lah)<br><br>Reference DOI:<br>10.1021/jacs.9b06580
UA79_Tah	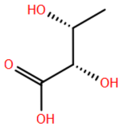	α-hydroxy acid of Thr (Tah)<br><br>Reference DOI:<br>10.1021/jacs.9b06580
UA80_PSeC	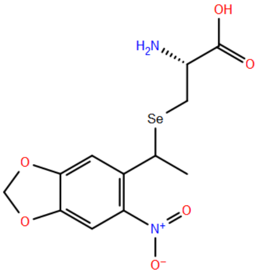	photocaged selenocysteine (PSc)<br><br>Reference DOI:<br>10.1021/acs.bioconjchem.8b00254

Index Name	UAA Formula	Full Name or Alias in Literature
UA81_5HTPW	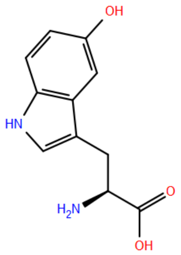	5-hydroxytryptophan (5HTP)<br><br>Reference DOI:<br>10.1002/cbic.201800111
UA82_Aze	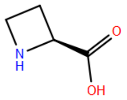	azetidine-2-carboxylic acid (Aze)<br><br>Reference DOI:<br>10.1021/acs.biochem.8b00379
UA83_Mor	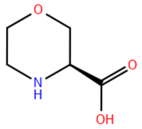	morpholine-3-carboxylic acid (Mor)<br><br>Reference DOI:<br>10.1021/acs.biochem.8b00379
UA84_nMeA	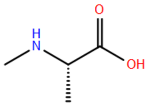	N-methylalanine (N-Me-Ala)<br><br>Reference DOI:<br>10.1021/acs.biochem.8b00379
UA85_3MeP	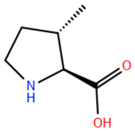	3-methylproline (3-Me-Pro)<br><br>Reference DOI:<br>10.1021/acs.biochem.8b00379
UA86_nMeL	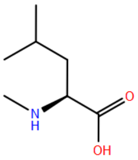	N-methylleucine (N-Me-Leu)<br><br>Reference DOI:<br>10.1021/acs.biochem.8b00379
UA87_Aah	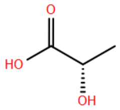	α-hydroxyalanine<br>(Aah, or lactic acid)<br><br>Reference DOI:<br>10.1021/acs.biochem.8b00379
UA88_F2AzoF	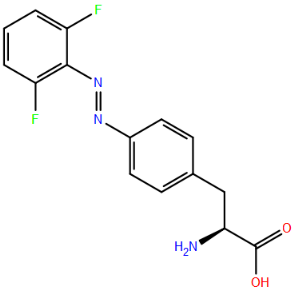	difluoro-4′-azobenzene-phenylalanine<br><br>azobenzene derivatives (F2AzoF)<br><br>Reference DOI:<br>10.1002/cbic.201800226

Index Name	UAA Formula	Full Name or Alias in Literature
UA89_F4AzoF	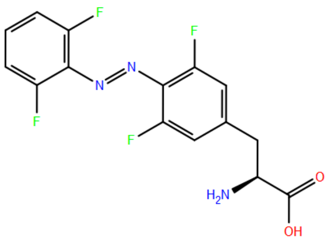	tetrafluoro-4′-azobenzene-phenylalanine<br><br>azobenzene derivatives (F4AzoF)<br><br>Reference DOI:<br>10.1002/cbic.201800226
UA90_AcdA	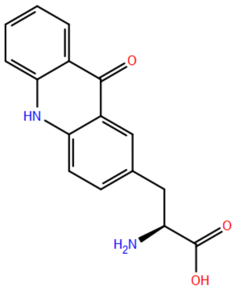	acridonylalanine (Acd)<br><br>acridon-2-ylalanine (1, Acd, δ)<br><br>Reference DOI:<br>10.1021/acschembio.8b00696<br>10.1021/ja403247j
UA91_StyrK	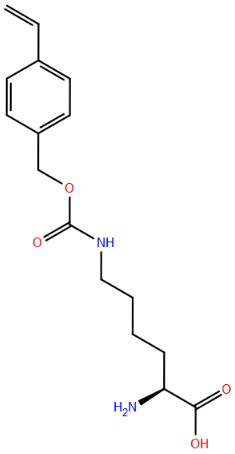	styrene lysine (KStyr)<br><br>Reference DOI:<br>10.1021/acs.bioconjchem.7b00562
UA92_MtY	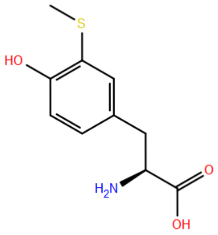	2-amino-3-(4-hydroxy-3-(methylthio)phenyl)propanoic acid<br><br>3-(methylthio)-tyrosine (MtTyr)<br><br>Reference DOI:<br>10.1021/jacs.7b13628
UA93_NEMmalC	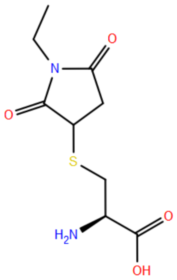	N-ethyl maleimide cysteine (NEM-Cys)<br><br>Reference DOI:<br>10.1016/j.jmb.2017.04.019

Index Name	UAA Formula	Full Name or Alias in Literature
UA94_TMAmalC	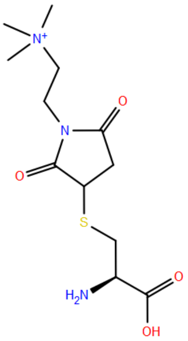	trimethylammonium maleimide Cys (TMA-MAL-Cys)<br><br>Reference DOI:<br>10.1016/j.jmb.2017.04.019
UA95_TEAmalC	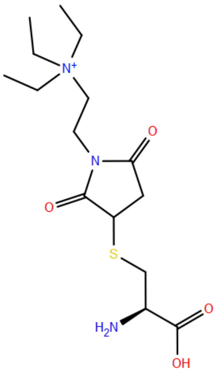	triethylammonium maleimide Cys (TEA-MAL-Cys)<br><br>Reference DOI:<br>10.1016/j.jmb.2017.04.019
UA96_TBAmalC	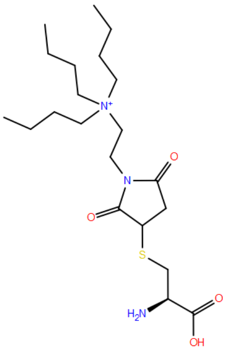	tributylammonium maleimide Cys (TBA-MAL-Cys)<br><br>Reference DOI:<br>10.1016/j.jmb.2017.04.019
UA97_NPY	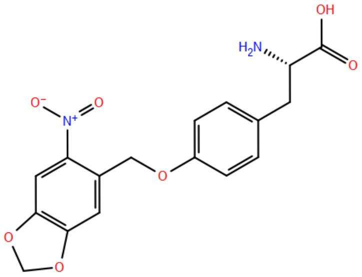	nitropiperonyl tyrosine (NPY)<br><br>Reference DOI:<br>10.1002/cbic.201700147
UA98_MNPY	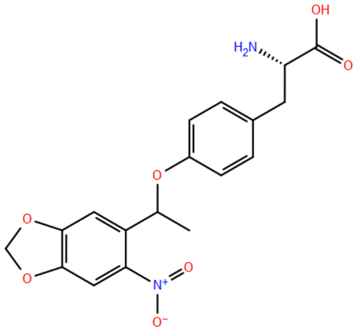	methylnitropiperonyl tyrosine (MNPY)<br><br>Reference DOI:<br>10.1002/cbic.201700147

Index Name	UAA Formula	Full Name or Alias in Literature
UA99_NPPY	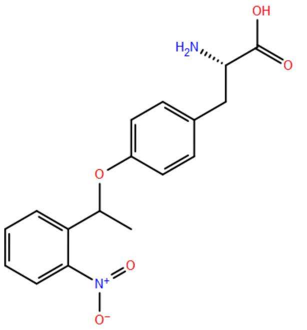	nitrophenylpropyl tyrosine (NPPY)<br><br>Reference DOI:<br>10.1002/cbic.201700147
UB00_NBY	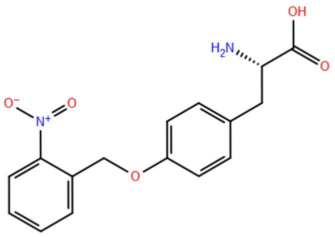	nitrobenzyl tyrosine (NBY)<br>o-nitrobenzyl-tyrosine (oNBTyr)<br><br>Reference DOI:<br>10.1002/cbic.201700147<br>10.1021/ja801602q
UB01_BPKyne	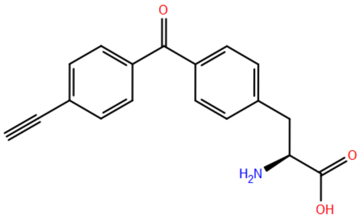	4’-ethynyl-p-benzoyl-l-phenylalanine (BPKyne)<br><br>Reference DOI:<br>10.1002/cbic.201600578
UB02_CytA	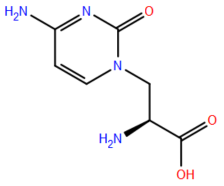	cytosine alanine (CytA)<br>alanyl nucleobase amino acids (A)<br><br>Reference DOI:<br>10.1016/j.bmc.2016.07.008
UB03_UraA	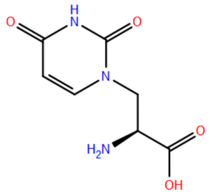	uracil alanine (UraA)<br>alanyl nucleobase amino acids (B)<br><br>Reference DOI:<br>10.1016/j.bmc.2016.07.008
UB04_ThyA	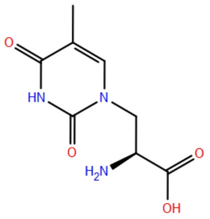	thymine alanine (ThyA)<br>alanyl nucleobase amino acids (C)<br><br>Reference DOI:<br>10.1016/j.bmc.2016.07.008
UB05_AdeA	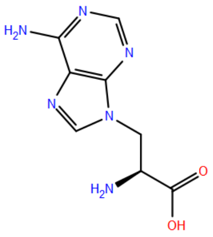	adenine alanine (AdeA)<br>alanyl nucleobase amino acids (D)<br><br>Reference DOI:<br>10.1016/j.bmc.2016.07.008

Index Name	UAA Formula	Full Name or Alias in Literature
UB06_GuaA	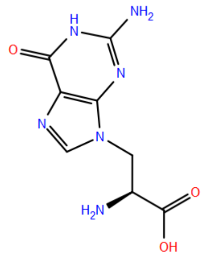	guanine alanine (GuaA)<br>alanyl nucleobase amino acids (E)<br><br>Reference DOI:<br>10.1016/j.bmc.2016.07.008
UB07_adonY	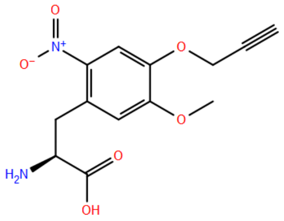	O-propargyl-2-nitro-5-methoxy tyrosine<br><br>tyrosine with an alkyne modified dimethoxy-ortho-nitrobenzyl caging group (adonY)<br><br>Reference DOI:<br>10.1016/j.tetlet.2016.09.033
UB08_pAzmF	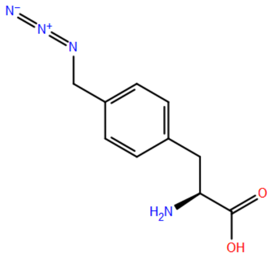	p-azidomethylphenylalanine (AzMeF) (8)<br><br>Reference DOI:<br>10.1002/cbic.201500695<br>10.1073/pnas.1507741112
UB09_AminoY	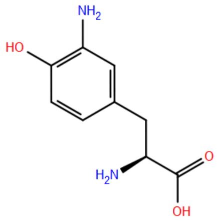	3-amino-L-tyrosine (AminoY)<br><br>Reference DOI:<br>10.1021/acssynbio.6b00192
UB10_pTmdZLys	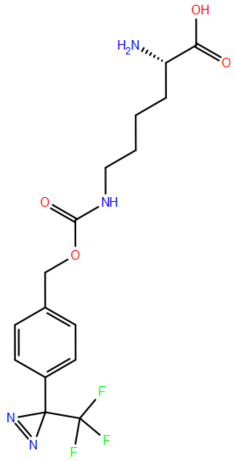	Nε-(p-trifluoromethyldiazirinyl-benzyloxycarbonyl)-L-lysine (pTmdZLys)<br><br>Reference DOI:<br>10.1038/srep36946

Index Name	UAA Formula	Full Name or Alias in Literature
UB11_mTmdZLys	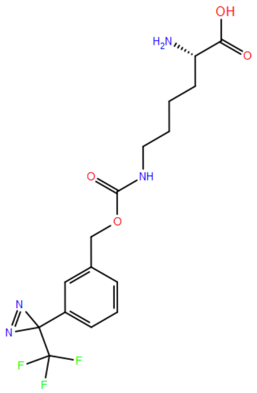	Nε-(m-trifluoromethyldiazirinyl-benzyloxycarbonyl)-L-lysine (mTmdZLys)<br><br>Reference DOI:<br>10.1038/srep36946
UB12_IndA	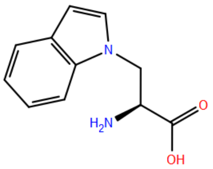	2-amino-3-indol-1-yl-propionic acid (Ind)<br><br>Reference DOI:<br>10.1021/acschemneuro.5b00298<br>10.1074/jbc.M114.631960
UB13_CHA	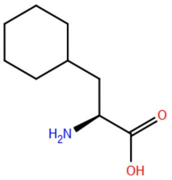	cyclohexylalanine (CHA)<br><br>Reference DOI:<br>10.1074/jbc.M114.631960
UB14_Lyk	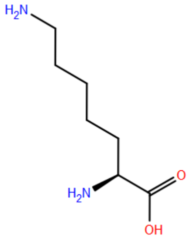	lysine analogue, 2,7-diaminoheptanoic acid (Lyk)<br><br>Reference DOI:<br>10.1021/bi501138b
UB15_F2Y	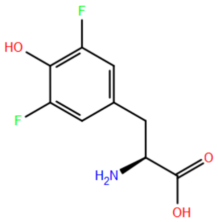	3,5-difluorotyrosine (F2Tyr, F2Y)<br><br>Reference DOI:<br>10.1021/ja5109936<br>10.1038/ncomms9202
UB16_F3Y	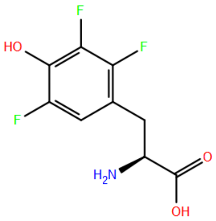	2,3,5-trifluorotyrosine (F3Tyr, F3Y)<br><br>Reference DOI:<br>10.1021/ja5109936<br>10.1002/cbic.201400051

Index Name	UAA Formula	Full Name or Alias in Literature
UB17_3OMeY	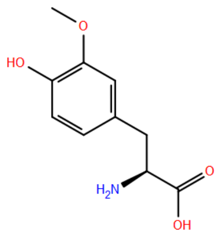	3-methoxytyrosine (OMeY)<br><br>Reference DOI:<br>10.1039/C5SC01126D
UB18_AcrF	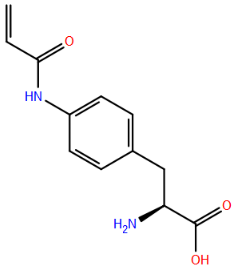	p-acrylamido-phenylalanine (AcrF) (3)<br><br>Reference DOI:<br>10.1073/pnas.1507741112
UB19_OallylY	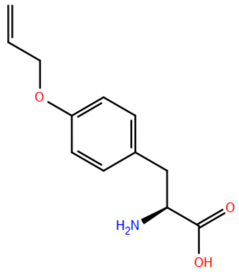	O-allyl-L-tyrosine<br>O-allyl-tyrosine (5) (16) (OAY)<br><br>Reference DOI:<br>10.1073/pnas.1507741112<br>10.1016/j.bmcl.2011.09.108<br>10.1038/nbt742
UB20_PheF	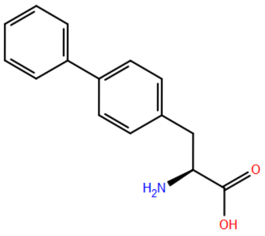	p-phenyl-L-phenylalanine (9) (17)<br><br>Reference DOI:<br>10.1073/pnas.1507741112<br>10.1016/j.bmcl.2011.09.108
UB21_OtBuY	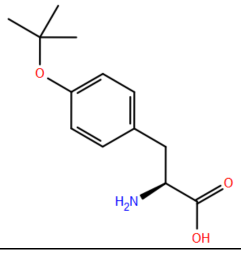	O-tert-Butoxy-tyrosine (OtBuY) (10)<br><br>Reference DOI:<br>10.1073/pnas.1507741112
UB22_CanR	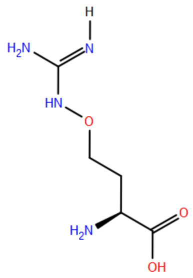	L-Canavanine (Can)<br><br>Reference DOI:<br>10.1016/j.bmcl.2015.06.045

Index Name	UAA Formula	Full Name or Alias in Literature
UB23_ForK	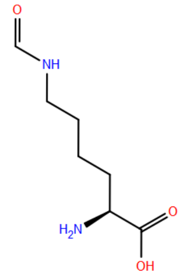	Nε-formyllysine (ForK)<br><br>Reference DOI:<br>10.1002/cbic.201500170
UB24_sTyr	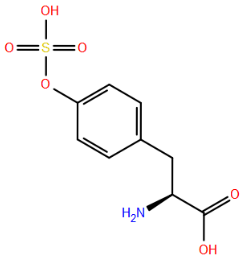	sulfotyrosine (SO3Tyr, sTyr)<br><br>Reference DOI:<br>10.1021/sb500195w<br>10.1073/pnas.1110042108
UB25_FNO2F	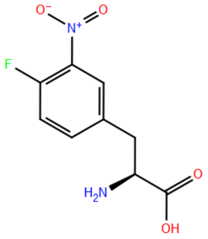	4-fluoro-3-nitrophenylalanine (FNO2Phe)<br><br>Reference DOI:<br>10.1021/jacs.5b03652
UB26_BFF	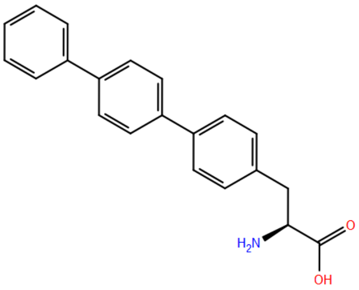	4-biphenyl-L-phenylalanine (terphenyl alanine)<br><br>Reference DOI:<br>10.1016/j.bmcl.2015.09.050
UB27_CitR	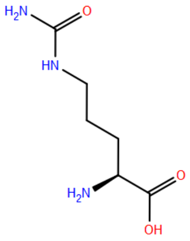	citrulline (Cit)<br><br>Reference DOI:<br>10.1021/bi501477y
UB28_dmPyl	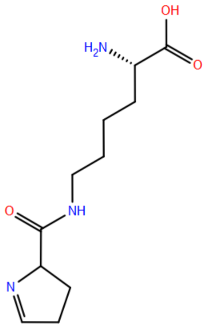	desmethylpyrrolysine (dmPyl)<br><br>Reference DOI:<br>10.1002/chem.201500971

Index Name	UAA Formula	Full Name or Alias in Literature
UB29_ePyl	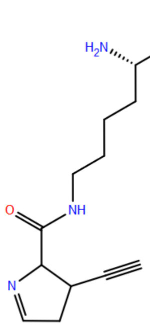	ethynylpyrrolysine (ePyl)<br><br>Reference DOI:<br>10.1002/chem.201500971
UB30_PCK	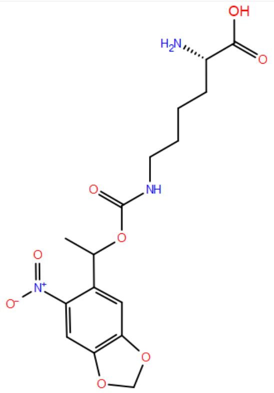	photocaged lysine (1) (4)<br><br>Reference DOI:<br>10.1002/cbic.201400073<br>10.1021/ja104609m
UB31_PCHcy	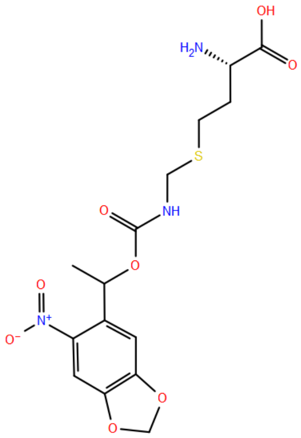	photocaged homocysteine (Hcy) (2)<br><br>Reference DOI:<br>10.1002/cbic.201400073
UB32_pCmF	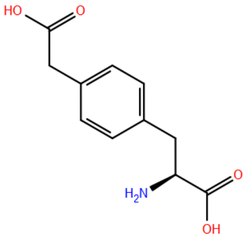	p-carboxymethyl-L-phenylalanine (pCmF)<br><br>Reference DOI:<br>10.1021/cb400859z<br>10.1038/nchembio.657<br>10.1016/j.jmb.2009.10.030
UB33_3NO2Y	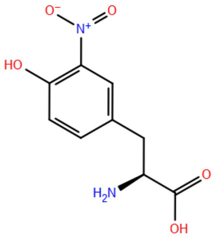	3-nitrotyrosine (3NO2Tyr)<br><br>Reference DOI:<br>10.1074/jbc.M114.556506<br>10.1073/pnas.1110042108

Index Name	UAA Formula	Full Name or Alias in Literature
UB34_BrbF	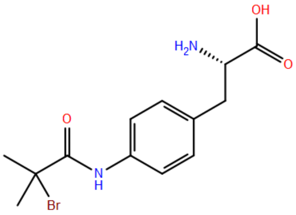	4-(2′-bromoisobutyrlamido)-phenylalanine (2, Brb, β)<br><br>Reference DOI:<br>10.1021/ja403247j
UB35_dmnC	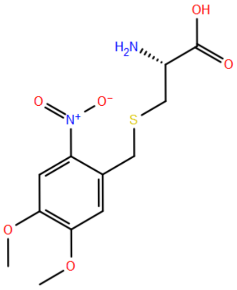	4,5-dimethoxy-2-nitrobenzyl-cysteine (Cmn, mnC, dmnC)<br><br>Reference DOI:<br>10.1016/j.neuron.2013.08.016
UB36_CpA	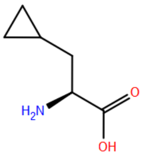	beta-cyclopropylalanine (Cpa) (2)<br><br>Reference DOI:<br>10.1039/c2mb25193k
UB37_3FY	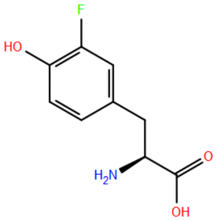	3-fluoro-L-tyrosines<br>(3FY) (m-F-Tyr) (4)<br><br>Reference DOI:<br>10.1016/j.bmcl.2011.09.108<br>10.1016/j.pep.2004.07.019
UB38_Br2Y	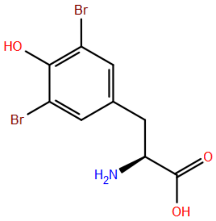	3,5-dibromotyrosine (5)<br><br>Reference DOI:<br>10.1016/j.bmcl.2011.09.108
UB39_3VY	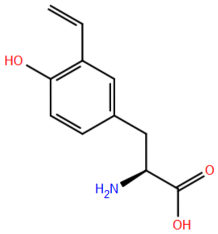	3-vinyl-L-tyrosines (6)<br><br>Reference DOI:<br>10.1016/j.bmcl.2011.09.108
UB40_pETF	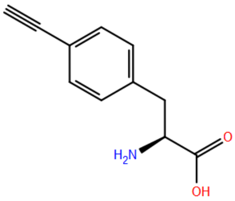	para-ethynylphenylalanine (13)<br>4-ethynylphenylalanine (pENPhe)<br><br>Reference DOI:<br>10.1016/j.bmcl.2011.09.108<br>10.1021/bi900426d

Index Name	UAA Formula	Full Name or Alias in Literature
UB41_McoAA	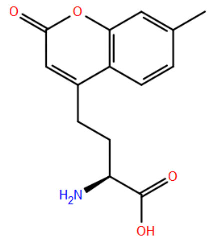	(7-methyl-4-coumarin-yl) ethylglycine (Mco)<br><br>Reference DOI:<br>10.1021/ja106416g
UB42_HNV	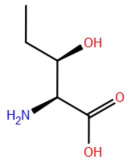	β-Hydroxynorvaline (HNV)<br><br>Reference DOI:<br>10.1021/bi101360a
UB43_DiZPK	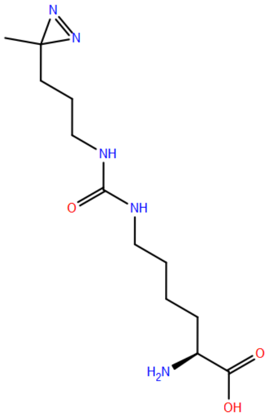	((3-(3-methyl-3H-diazirin-3-yl)propamino)carbonyl)-Nε-L-lysine (DiZPK)<br><br>Reference DOI:<br>10.1021/ja209008w
UB44_ACPK	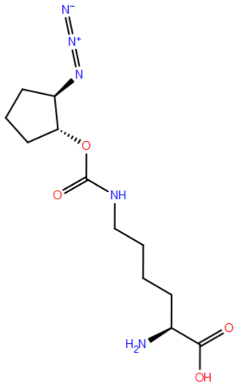	Nε-((((1R,2R)-2-azidocyclopentyl)oxy)carbonyl)-L-lysine (ACPK)<br><br>Reference DOI:<br>10.1021/ja209008w
UB45_PLA	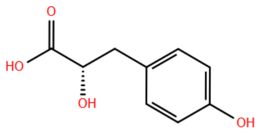	p-hydroxy-L-phenyllactic acid (PLA)<br><br>Reference DOI:<br>10.1016/j.jmb.2009.10.030
UB46_pBoF	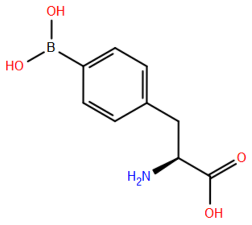	p-boronophenylalanine (pBoF, pBO2pa)<br><br>Reference DOI:<br>10.1016/j.jmb.2009.10.030<br>10.1371/journal.pone.0009354

Index Name	UAA Formula	Full Name or Alias in Literature
UB47_oNiF	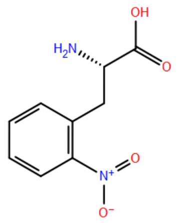	o-nitrophenylalanine (oNiF)<br><br>Reference DOI:<br>10.1016/j.jmb.2009.10.030
UB48_pTpa	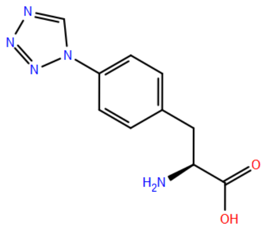	p-(2-tetrazole)phenylalanine (p-Tpa)<br><br>Reference DOI:<br>10.1021/ja104350y
UB49_pmmfF	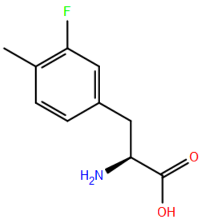	3-fluoro-4-methylphenylalanine (pmmfF)<br><br>Reference DOI:<br>10.1021/bi901947r
UB50_CF3COK	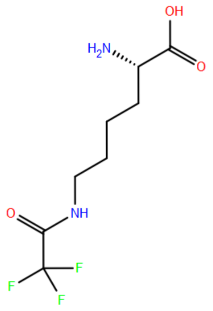	Nε-trifluoromethylcarbonyl-L-lysine (3)<br><br>Reference DOI:<br>10.1021/ja104609m
UB51_NapA	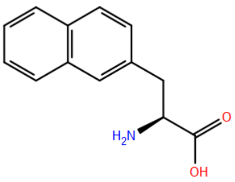	L-2-naphthylalanine (Nap)<br>3-(2-naphthyl)alanine (NapA)<br><br>Reference DOI:<br>10.1016/j.jmb.2009.10.030<br>10.1039/b904032c
UB52_CycK	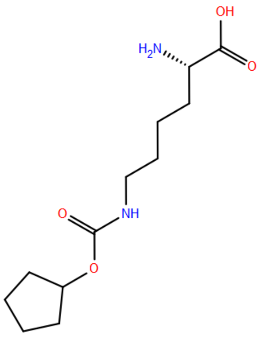	Nε-cyclopentyloxycarbonyl-L-lysine (Cyc)<br><br>Reference DOI:<br>10.1002/anie.200900683

Index Name	UAA Formula	Full Name or Alias in Literature
UB53_ONBK	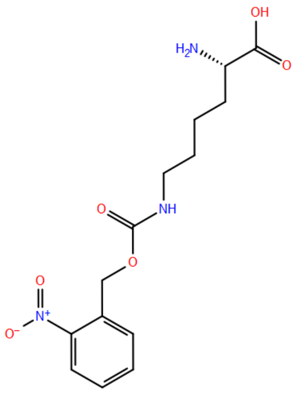	o-nitrobenzyl-oxycarbonyl-Nε-L-lysine (ONBK)<br><br>Reference DOI:<br>10.1002/anie.200900683
UB54_OCF3Y	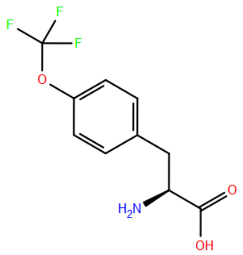	O-trifluoromethyl-tyrosine (OCF3Y)<br><br>O-trifluoromethyl-phenylalanine (OCF3Phe)<br><br>2-amino-3-(4-(trifluoromethoxy)-phenyl)propanoic acid<br><br>Reference DOI:<br>10.1021/ja801602q
UB55_NMeF	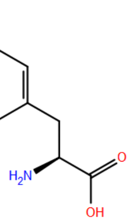	aminomethyl phenylalanine<br><br>Reference DOI:<br>10.1021/bi061772w
UB56_NapY	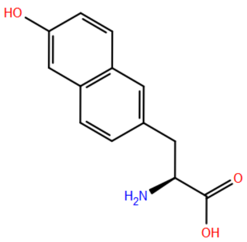	2-naphthyl tyrosine<br><br>Reference DOI:<br>10.1021/bi061772w
UB57_pDkF	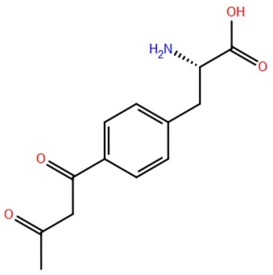	p-diketone-L-phenylalanine (pDkF)<br><br>Reference DOI:<br>10.1016/j.bmcl.2006.07.094

Index Name	UAA Formula	Full Name or Alias in Literature
UB58_OMeS	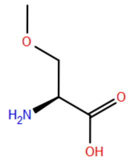	O-methylserine<br><br>Reference DOI:<br>10.1074/jbc.M508635200
UB59_OMeT	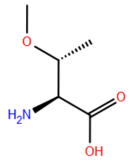	O-methylthreonine<br><br>Reference DOI:<br>10.1074/jbc.M508635200
UB60_norY	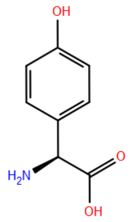	nortyrosine (nor-Tyr)<br><br>Reference DOI:<br>10.1016/j.pep.2004.07.019
UB61_BzFurA	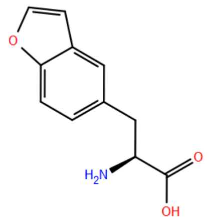	benzofuranylalanine (1) (Bzf)<br><br>Reference DOI:<br>10.1074/jbc.M401278200
UB62_BzTazA	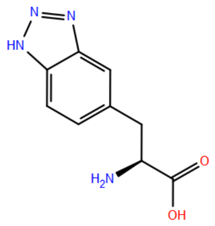	benzotriazolylalanine (2)<br><br>Reference DOI:<br>10.1074/jbc.M401278200
UB63_hGln	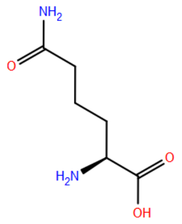	L-homoglutamine (hGln)<br><br>Reference DOI:<br>10.1073/pnas.0401517101
UB64_F2W	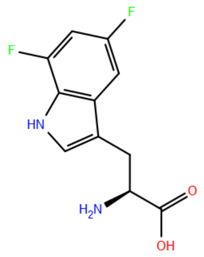	5,7-difluorotryptophan (F2Trp)<br><br>Reference DOI:<br>10.1016/s1074-5521(03)00124-8

Index Name	UAA Formula	Full Name or Alias in Literature
UB65_pCF	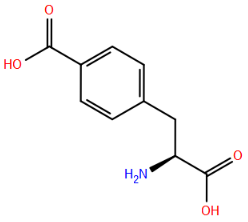	p-carboxyl-phenylalanine (pCF)<br><br>Reference DOI:<br>10.1038/nbt742
UB66_pIF	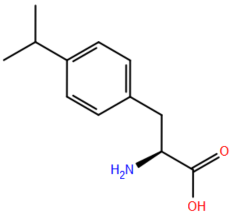	p-isopropyl-phenylalanine (pIF, iodoF)<br><br>Reference DOI:<br>10.1038/nbt742
UB67_O2QY	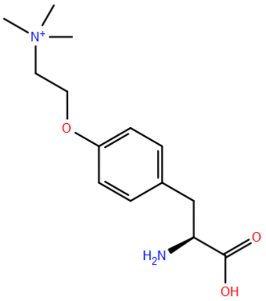	(Tyr-OnQ, n=2) tyrosine with 2 methylene-tethered quaternary ammonium<br><br>Reference DOI:<br>10.1016/s1074-5521(00)00055-7
UB68_O3QY	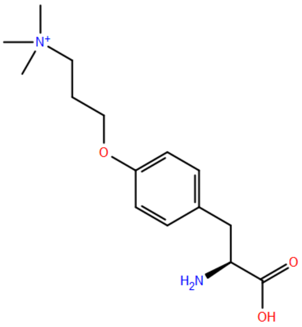	(Tyr-OnQ, n=3) tyrosine with 3 methylene-tethered quaternary ammonium<br><br>Reference DOI:<br>10.1016/s1074-5521(00)00055-7
UB69_O4QY	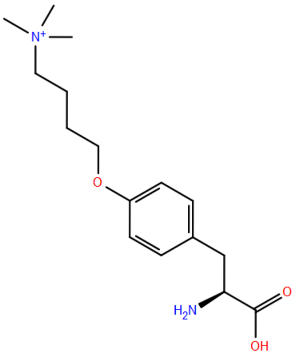	(Tyr-OnQ, n=4) tyrosine with 4 methylene-tethered quaternary ammonium<br><br>Reference DOI:<br>10.1016/s1074-5521(00)00055-7

Index Name	UAA Formula	Full Name or Alias in Literature
UB70_O5QY	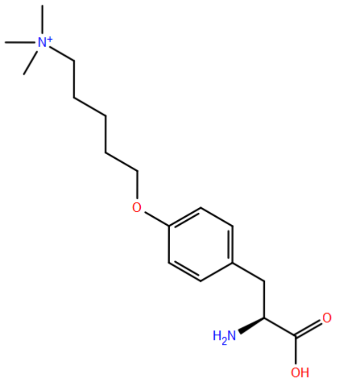	(Tyr-OnQ, n=5) tyrosine with 5 methylene-tethered quaternary ammonium<br><br>Reference DOI:<br>10.1016/s1074-5521(00)00055-7
UB71_O3tBuY	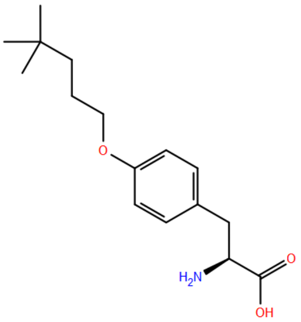	(Tyr-O3tBu) tyrosine with 3 methylene-tethered tert-butyl group<br><br>Reference DOI:<br>10.1016/s1074-5521(00)00055-7
UB72_PCC3	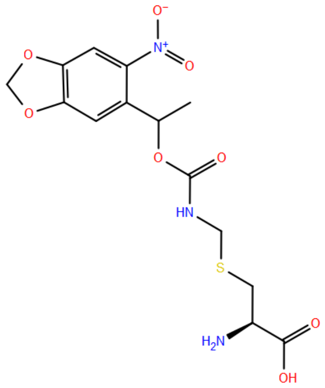	photocaged cysteine (3)<br><br>Reference DOI:<br>10.1002/cbic.201400073

Besides the UAAs, the formulae of 20 canonical/natural amino acids (AAs) were also drawn in the Schrodinger Canvas software to generate the similarity matrix between UAAs and AAs. The nomenclature of AAs takes the form of “AAnn_X_Abb”, where “AA” denotes amino acid, “nn” denotes two numbers, “X” denotes the 1-letter abbreviation of the amino acid, “Abb” denotes the 3-letter abbreviation of the amino acid, and the underscore “_” separates the index part, 1- and 3-letter abbreviation parts. Names of 20 canonical amino acids were listed in the table below:

AA01_A_Ala	AA02_C_Cys	AA03_D_Asp	AA04_E_Glu	AA05_F_Phe
AA06_G_Gly	AA07_H_His	AA08_I_Ile	AA09_K_Lys	AA10_L_Leu
AA11_M_Met	AA12_N_Asn	AA13_P_Pro	AA14_Q_Gln	AA15_R_Arg
AA16_S_Ser	AA17_T_Thr	AA18_V_Val	AA19_W_Trp	AA20_Y_Tyr

Since more and more UAAs will be added, we put 20 AAs at the top of the database. After preparing the formulae of 20 AAs and all UAAs, the physiochemical properties and similarity matrix were generated likewise and export to csv files.

Chapter 3: Manage the Database of Known UAA Sites

In 2001, Schultz PG et al. developed the method of site-specific UAA incorporation into proteins by genetic code expansion or nonsense suppression with engineered tRNA and aminoacyl-tRNA synthetase (aaRS) pairs. Since then, a number of researches on this topic were conducted and more than 170 kinds of UAAs has been incorporated into different proteins. We collected the UAA substitution information from those researches published in the last 20 years (from 2001 to 2021), and made a database of experimentally verified UAA sites (the database of known UAA sites) for machine learning and prediction.

The database is saved in the csv format as "known_uaa_sites.csv" in the "residues" subfolder and partially maintained by the RPDUAA program. For RPDUAA version (1.0), a total of 1221 UAA substitution records were included in the database. Users can browse, sort, filter, modify, or even delete records by open "known_uaa_sites.csv" with Microsoft Excel. **Be aware to make a copy of "known_uaa_sites.csv" for recovery.**

To append more records of UAA substitutions to the database, uses are recommended to use RPDUAA, since RPDUAA could conveniently call the previously analyzed protein structure, sequence and alignment information (i.e., the ready-to-use csv file in the "proteins" subfolder, see the protein analysis steps in Chapter 1).

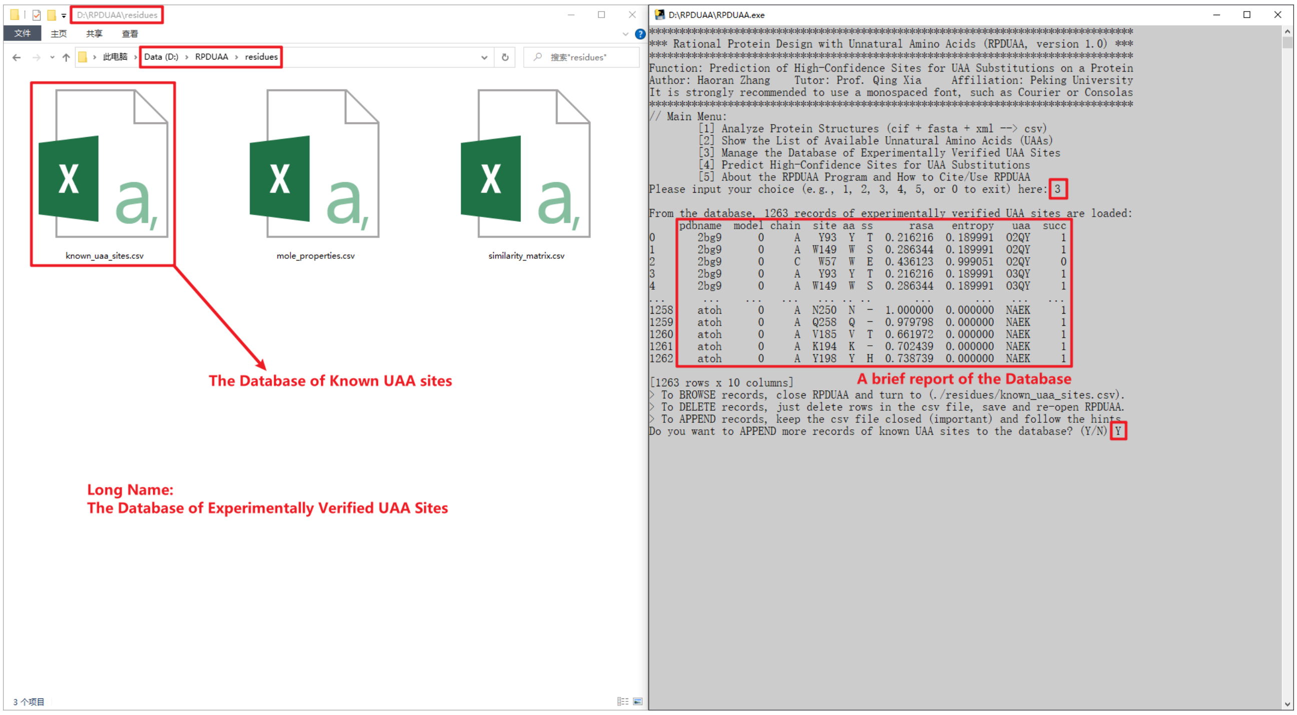

Now, let's do an exercise of appending new UAA substitution records to the database. We are going to substitute **Y151-TAG** site of superfolder green fluorescent protein (sfGFP, PDB ID: **2b3p**) with **NAEK** (indexed UAA name: UA22_NAEK). This substitution site was experimentally verified in the paper (DOI: **10.1038/s41589-020-0507-z**).

In the Main Menu of RPDUAA, choose Task [3] by inputting "3" and pressing "Enter". The RPDUAA program will return a brief report of the database and give some hints about how to browse, delete or append records. In the next question, input "Y" to start appending new records, or input "N" to cancel appending.

After choosing “Y”, the RPDUAA program will print a full list of available UAAs for you to choose one. Just input “UA22” to choose NAEK and press “Enter”. If you regret and do not append any record, you can just input “cancel” at this stage or any following stages, which will cancel appending and return to the Main Menu.

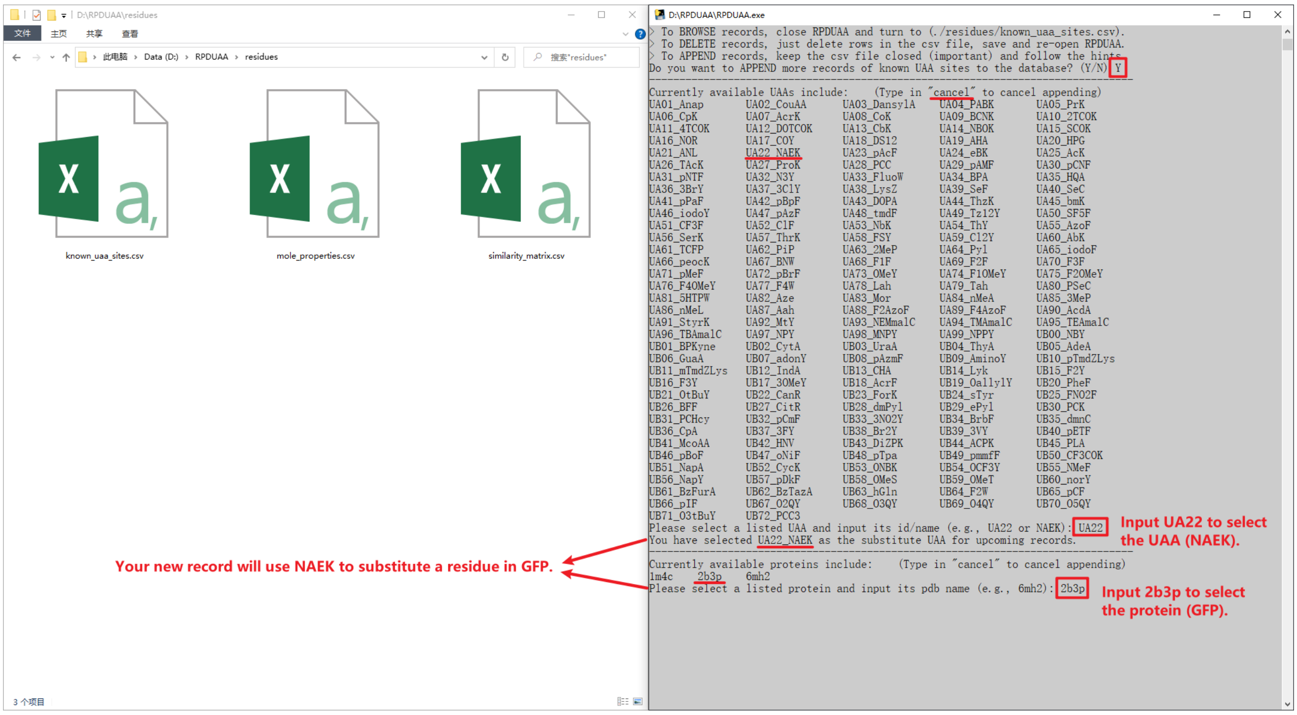

After choosing the UAA (UA22_NAEK), the RPDUAA program will list out the previously analyzed proteins and ask you to choose a protein. Input “2b3p” and press “Enter”. Then the RPDUAA program will list out the chains of protein 2b3p. Input “A” to select chain A which contains the substitution site and press “Enter”.

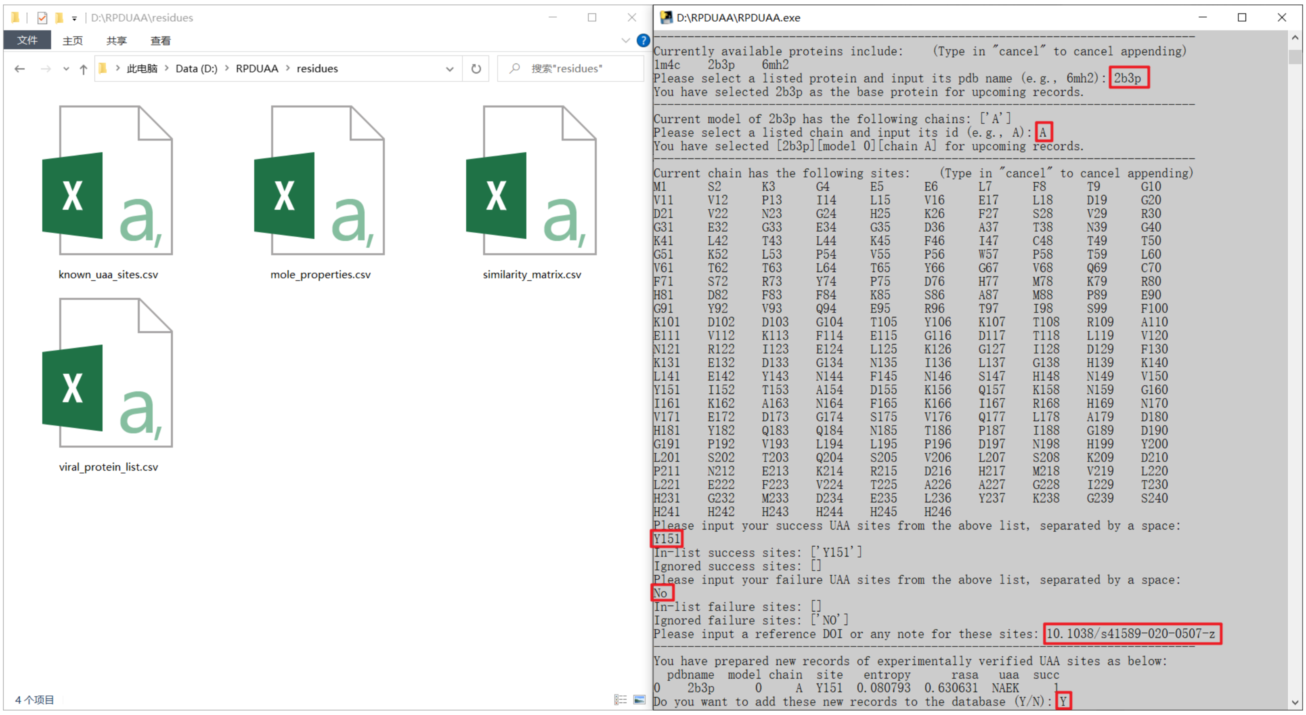

Then the RPDUAA program will list out all sites/positions/residues of chain A of protein 2b3p, and our goal is the Y151 site, which is a successful UAA substitution

site according to the published paper (DOI: 10.1038/s41589-020-0507-z). The RPDUAA program will ask you to input the success and failure UAA sites separately, both of which support inputting multiple sites (just separate your input multiple sites by a space, such as “F8 G10 Q94 Y182”). So, input “Y151” at the success line and input “No” at the failure line. The RPDUAA program will screen the in-list sites and ignore the out-of-list sites to normalize your input. For those in-list sites, the RPDUAA program will offer you a chance to input UAA incorporation efficiency. You can input 0.2835 or likewise, or simply input nothing if the article did not offer this efficiency. At the expanded codon line, input TAG to select amber codon.

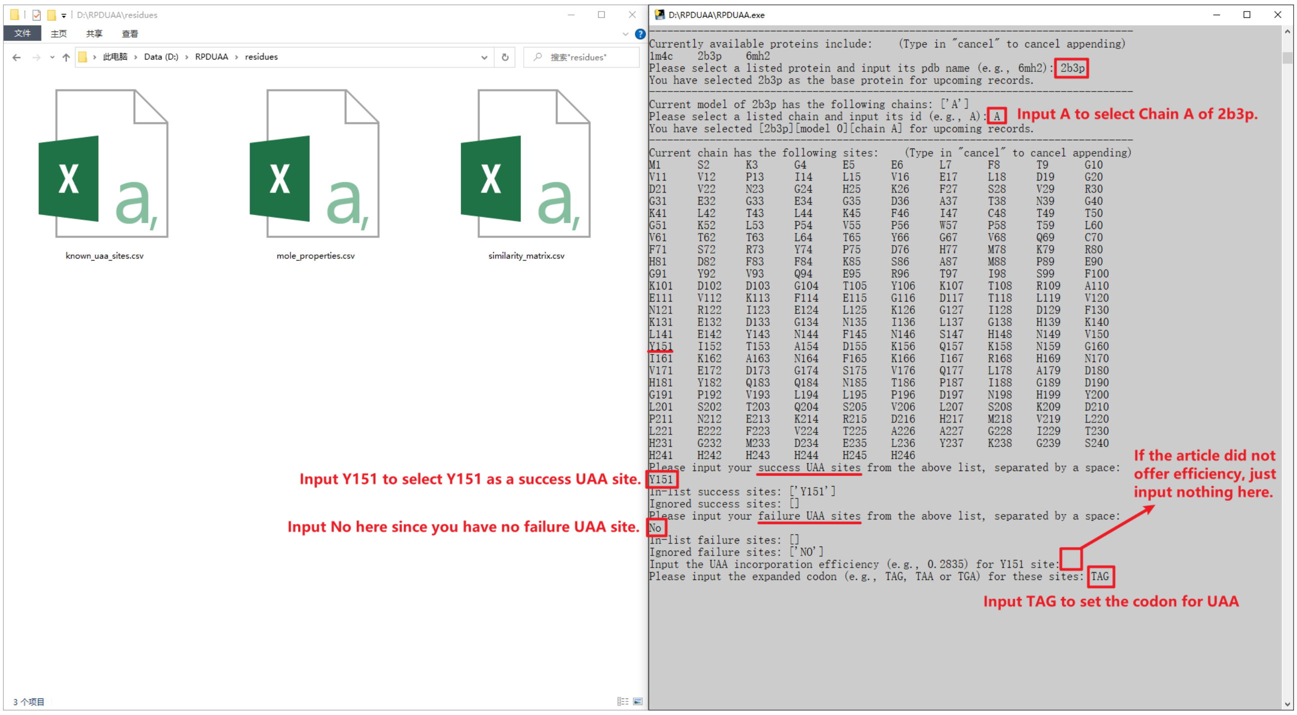

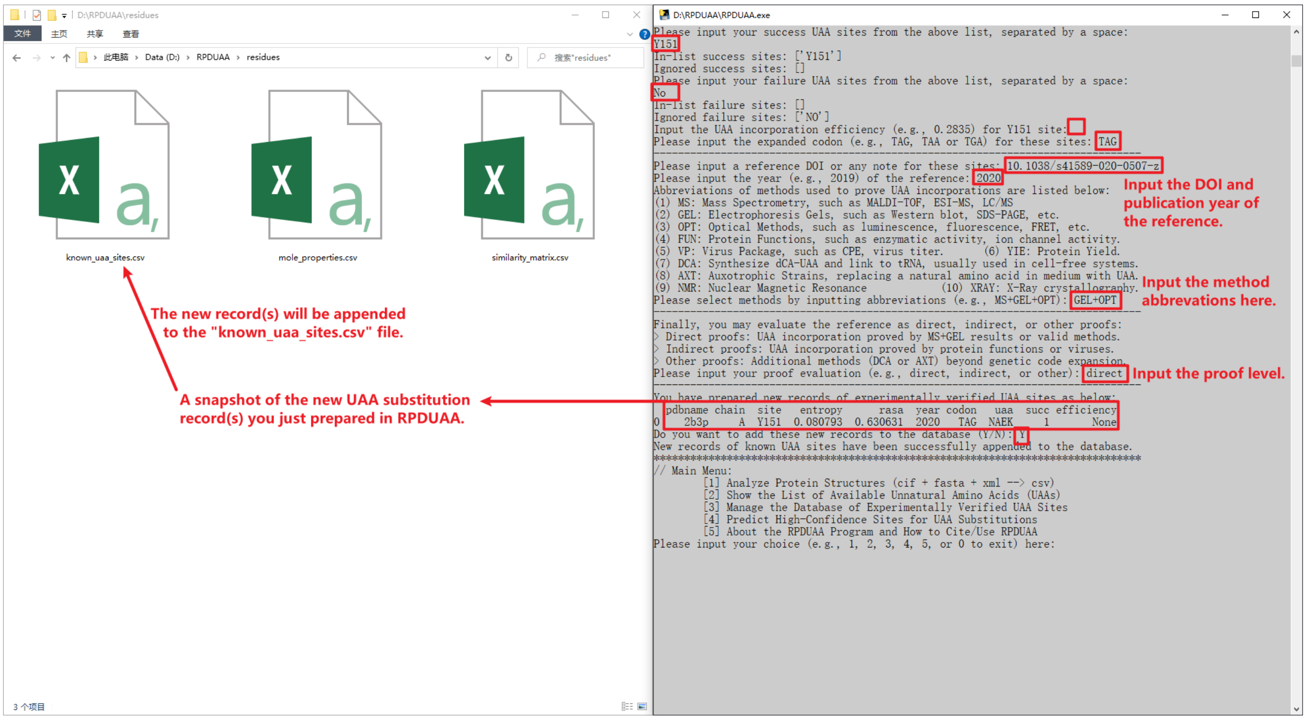

Then you need to input the DOI (10.1038/s41589-020-0507-z), publication year (2020), method abbreviation strings (GEL+OPT), and proof level (direct). After input all of them, the RPDUAA program will print a snapshot of the new record that you just prepared. You have the final chance to check the information of this new record before appending it to the database. Input "Y" to confirm appending, or "N" to cancel appending. The new records will be appended to the "known_uaa_sites.csv" file if you choose "Y".

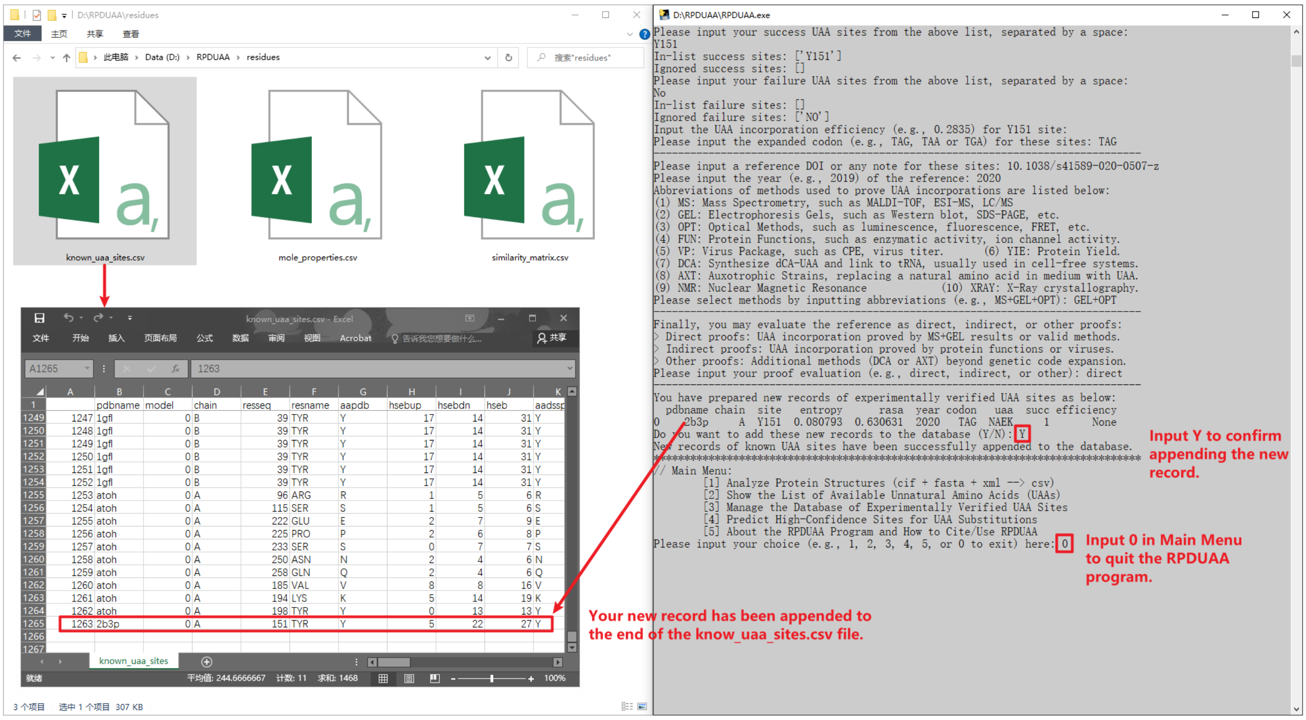

Task [3] in Main Menu of the RPDUAA program is over, and you can quit the RPDUAA program by inputting "0" in Main Menu and pressing "Enter". In this chapter, we showed how to use the RPDUAA program to append new records of UAA substitution sites to the Database of Experimentally Verified UAA Sites / Known UAA Sites.

As a pioneer, our team have prepared some records of UAA substitution sites from literature. These records are used as the training database of the RPDUAA program. Users can append their own new site to the database, and the author will continue to update the database in new releases of the RPDUAA program. A statistical report of the Database of Known UAA Sites in RPDUAA 1.0 is shown below:

- Full Name: The Database of Experimentally Verified UAA Substitution Sites
- Short Name and Date: <u>The Database of Known UAA Sites</u> (first release in 2021)
- Total Size: 1221 published records (excluding 42 unpublished in-lab records)
- Composition: 1064 success records and 157 failure records of UAA substitution
- Efficiency: 351 records with efficiency (124 records based on exact yield)
- Source: Collected from 196 research articles published between 2001 and 2021
- UAAs: 172 UAAs registered in total (the database only uses 156 UAAs of them)
- Proteins: 159 proteins in total (144 from PDB and 15 predicted structures)

Chapter 4: Performance of the Prediction Model

In the chapter, we will show the performance of the prediction model in RPDUAA, which could be tested with different subsets of the database of known UAA sites. Open the RPDUAA program and choose Task [4] in the Main Menu by inputting "4" and pressing "Enter". Nine subsets will emerge, which are:

> **Subset 1**: All records in the database (proof=direct/indirect/other) ****
> **Subset 2**: Records from miscellaneous results (proof=direct/indirect) ***
> **Subset 3**: Records strongly supported by MS+GEL results (proof=direct) *
> **Subset 4**: Balanced records (>300 proofs; equal successes and failures) **
> **Subset 5**: Non-predicted PDB records (>1000 proofs; solved structures) ***
> **Subset 6**: Non-redundant records (drop duplicate proofs in literature) **
> **Subset 7**: Non-viral protein records (excluding those viral proteins) *
> **Subset 8**: Viral protein records (those demonstrated by virus package) *
> **Subset 9**: Amber suppression records (the expanded codon is TAG or UAG) **

The number of asterisks after each subset indicates the degree of recommendation. Full details of these subsets are shown below:

> **Subset 1** will use all 1221 records or the whole database for further prediction.

> **Subset 2** will use direct proofs or indirect proofs from miscellaneous experiments of genetic code expansion, but will exclude proofs from other techniques such as UAA replacement in medium of auxotrophic strains (AXT) to general UAA incorporation, ligation of dCA-UAA to a tRNA for aminoacylation or cell-free protein synthesis.

> **Subset 3** will only use direct proofs of genetic code expansion which are typically demonstrated by mass spectrometry (MS), electrophoresis gels (GEL) or some well-established optical methods (OPT). Indirect proofs such as protein function (FUN) or viral package (VP) and other proofs will be excluded.

> **Subset 4** will use equal success and failure records as balanced records. Since the database contains much more success records than failure ones, we kept all failure records and randomly resampled equal success records to make this subset.

> **Subset 5** will use all the records involving solved protein structures directly downloaded from the Protein Data Bank (PDB) for further UAA substitution prediction, but will exclude records of in-silico-predicted protein structures by RoseTTAFold or AlphaFold2 (the pdbname of predicted proteins should not start with a number).

> **Subset 6** will remove duplicate records in the database. In previous researches, a UAA substitution record may be demonstrated by different researchers, such as substituting the Y39 site of GFP with NAEK. These records are thus redundant in the database. This subset will keep only the first one and discard the rest for these redundant records, so that the prediction will be based on the non-redundant records in the database.

> **Subset 7** will exclude those viral protein records from the database and use the rest for prediction, since viral protein records are based on special proofs.

> **Subset 8** will only use those viral protein records demonstrated by viral package.

> **Subset 9** will only use those records of amber suppression (codon=TAG/UAG).

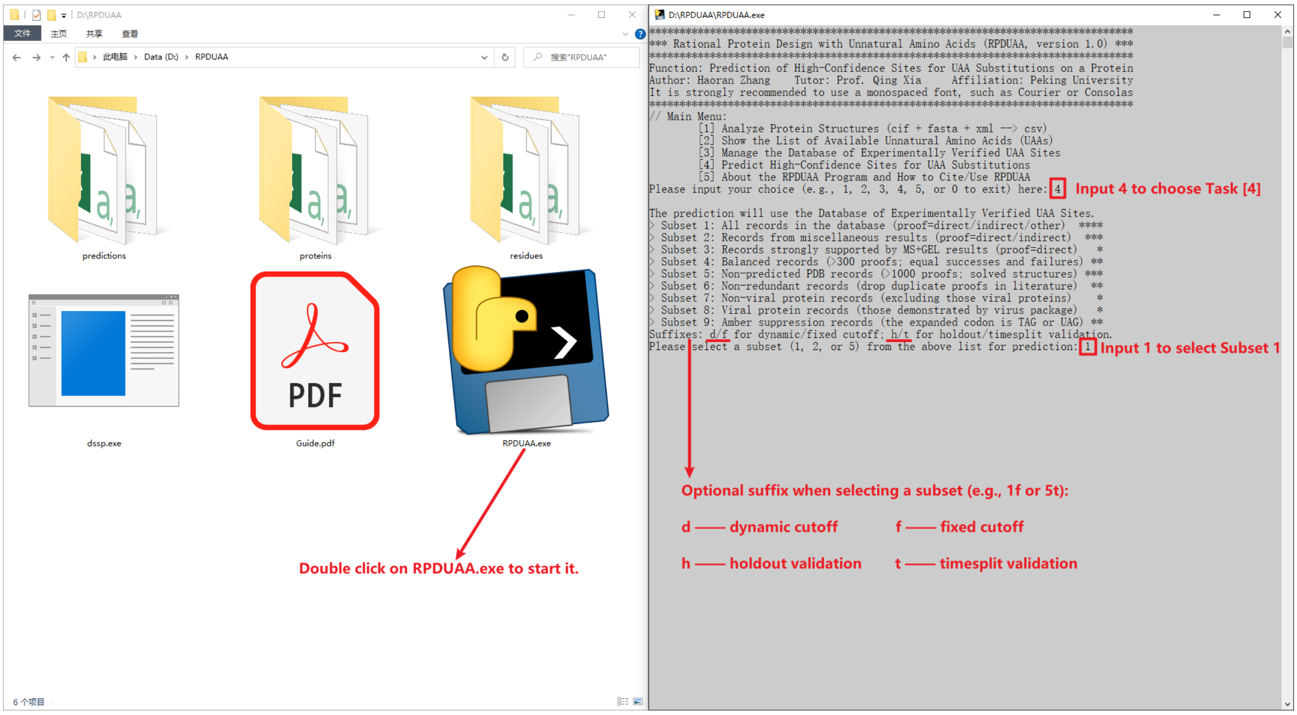

In the subset selection step, users can input “1”, “2”, “3” or “4”, with optional suffix “d” for dynamic cutoff (equal to the ratio of success records in a subset), suffix “f” for fixed cutoff (customized by you), suffix “h” for holdout validation (randomly selected 80% records for training, the rest 20% for testing and plotting), and suffix “t” for timesplit validation (example, training with “year<2018” records, testing and plotting with “year>=2018” records). If you select a subset without inputting any suffix, RPDUAA will use the whole subset for training, testing, and plotting, and the cutoff will be optimized by maximizing the sum of sensitivity and specificity. After inputting “1” and pressing “Enter”, a brief description of the prediction model and a 3-panel figure (PCA + scatters + ROC curve) will emerge:

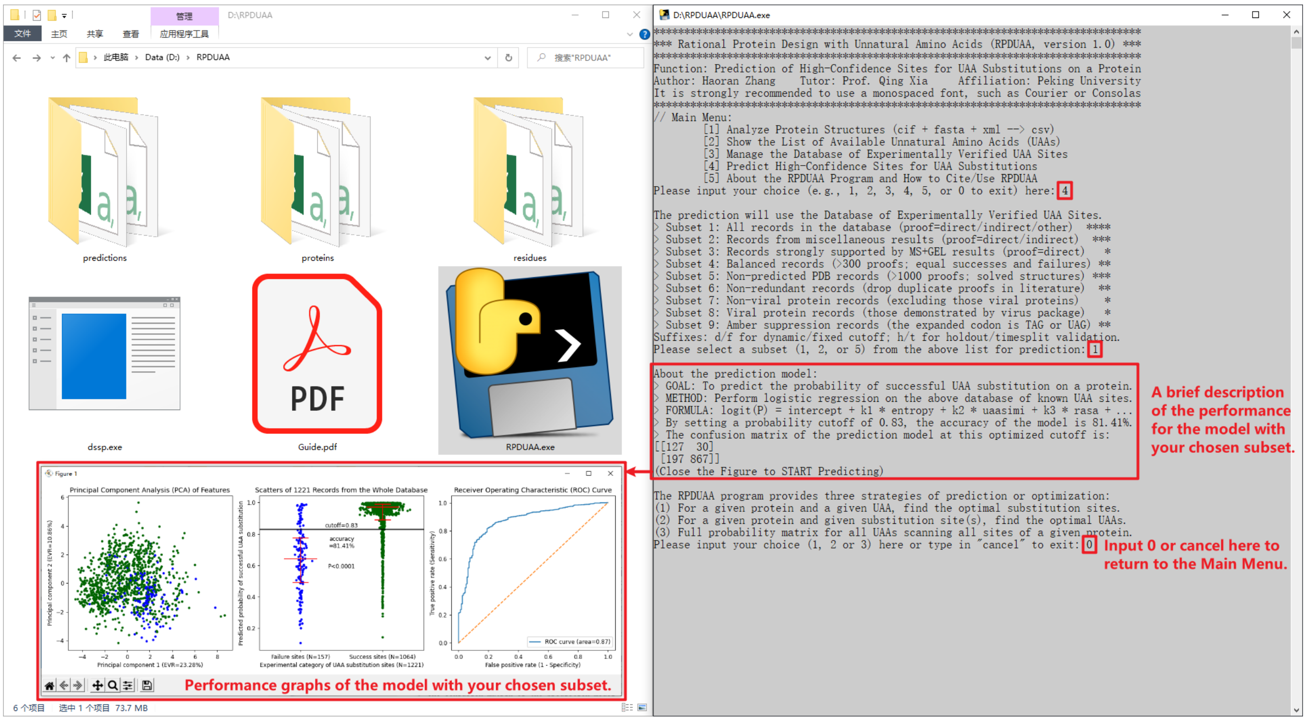

About the prediction model:
> GOAL: To predict the probability of successful UAA substitution on a protein.
> METHOD: Perform logistic regression on the above database of known UAA sites.
> FORMULA: logit(P) = intercept + k1 * entropy + k2 * uaasimi + k3 * rasa + ...
Performance of the prediction model based on **Subset 1**:
> By setting a probability cutoff of 0.83, the accuracy of the model is 81.41%.
> The confusion matrix of the prediction model at this optimized cutoff is:
[[127 30]
 [197 867]]
> The area under the ROC curve is 0.87, demonstrating good performance.

Besides using Subset 1 or the whole database, you may choose other subsets in Task [4] to check their performance in this prediction model. Some of the reporting graphs (for Subset 9, 2 and 6; ROC area larger than 0.85) are shown below:

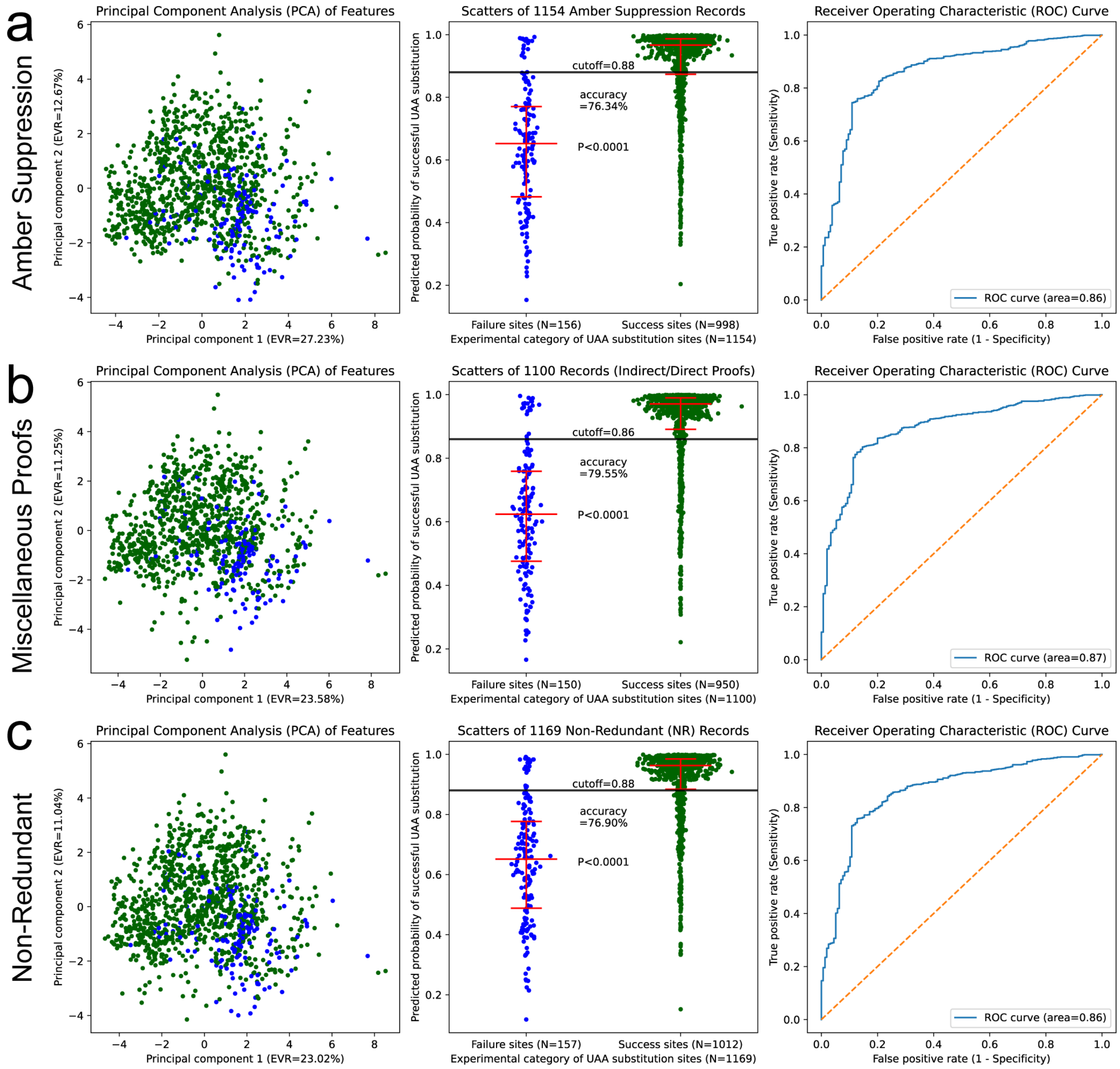

After collecting the key parameters of the prediction model on different subsets into a table (see below), we can make comparisons. Note that the accuracy is not stable for biased subsets and depends much on the optimized cutoff, while the ROC area is a stable index and serves as the golden standard of the model performance.

Database	Records	Cutoff	Accuracy	ROC area	Degree of recommendation
Subset 1	1221	0.83	81.41%	0.87	**** (the whole database)
Subset 2	1100	0.86	79.55%	0.87	*** (miscellaneous proofs)
Subset 3	625	0.98	68.37%	0.86	* (direct, highly-biased)
Subset 4	314	0.53	83.76%	0.89	** (balanced records)
Subset 5	1147	0.88	77.16%	0.87	*** (non-predicted PDB)
Subset 6	1169	0.88	76.90%	0.86	** (non-redundant)
Subset 7	974	0.93	82.34%	0.81	* (non-viral protein)
Subset 8	247	0.62	65.59%	0.70	* (viral protein)
Subset 9	1154	0.88	76.34%	0.86	** (amber suppression)

If you use Subset 4 (see below), you may find the optimized cutoff is around 0.50, different from other subsets. This is because Subset 4 is designed to enroll equal success and failure records. Other subsets are biased partly due to the fact that the literature tends to report success records more than failure ones. Choosing those with a probability above the optimal cutoff (~0.50 for Subset 4; ~0.85 for Subset 1 and other subsets) will give you more successful UAA substitution sites.

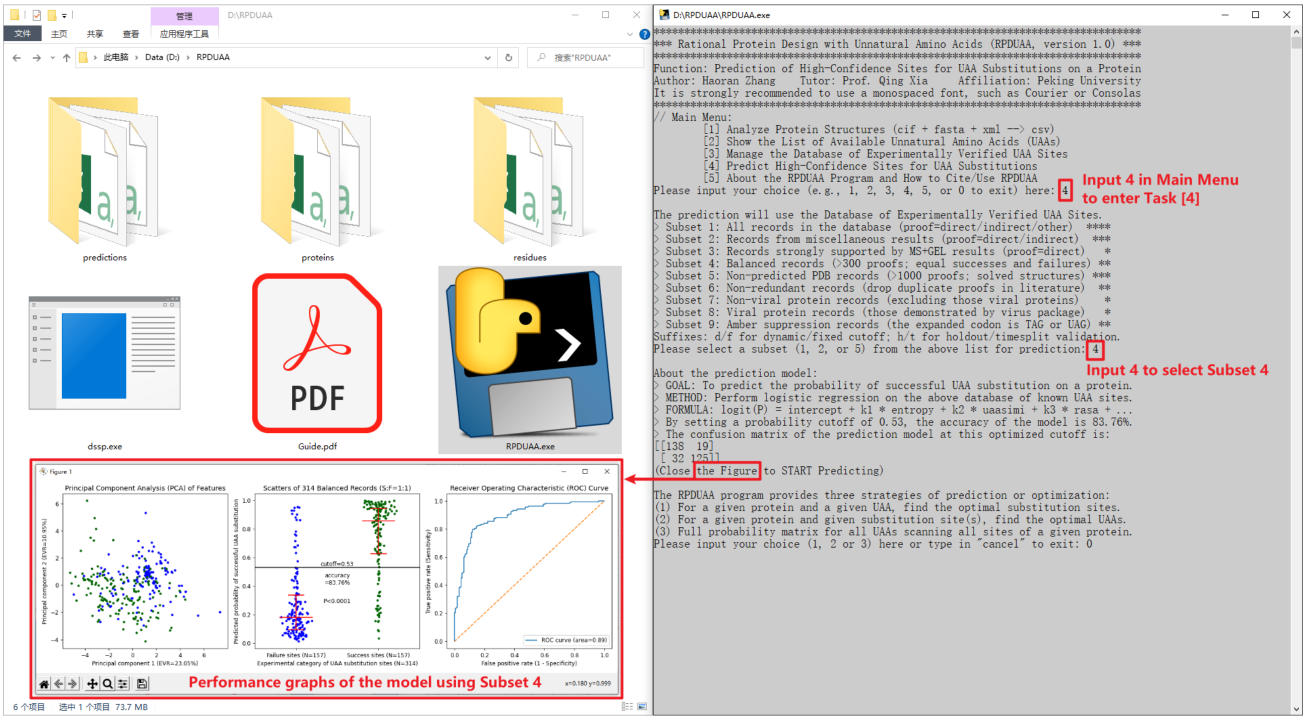

Suffix d/f to control the cutoff of the prediction model

When selecting a subset, you can add suffix d or suffix f to control the cutoff (e.g., 1d, 1f, 4f). If you input no suffix, the optimal cutoff will be realized by maximizing the sum of sensitivity and specificity by default, which corresponds to the most upper-left point on the ROC curve. If you input “1d”, the cutoff will

be set to S/(S+F), where S and F refer to the number of success and failure records, respectively. If you input “1f”, you will be further asked to customize a cutoff.

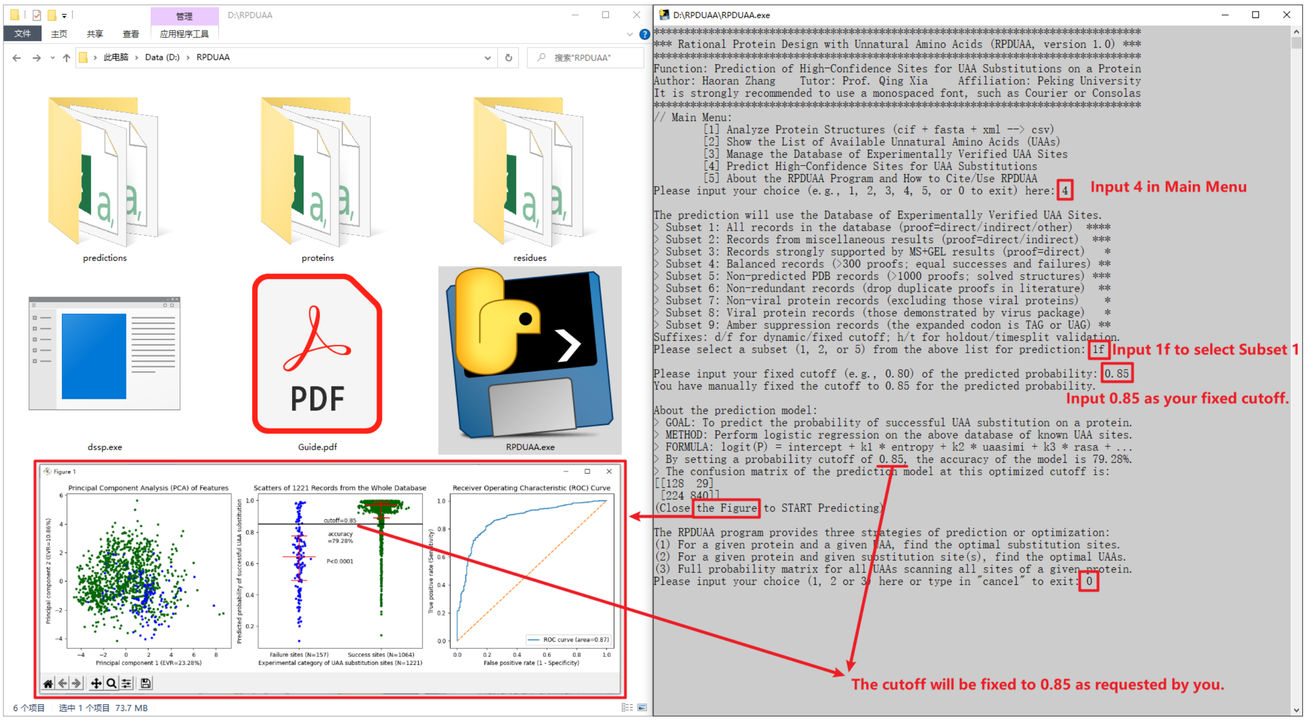

Suffix t for timesplit validation of the prediction model

The records in the database are collected from literature published between 2001 and 2021. You may split these records at the year 2019 to generate training data and testing data (i.e., train-test-split at 2019-1-1) to validate the performance of the prediction model. This can be done by adding a suffix “t” when selecting the subset in RPDUAA, as shown below:

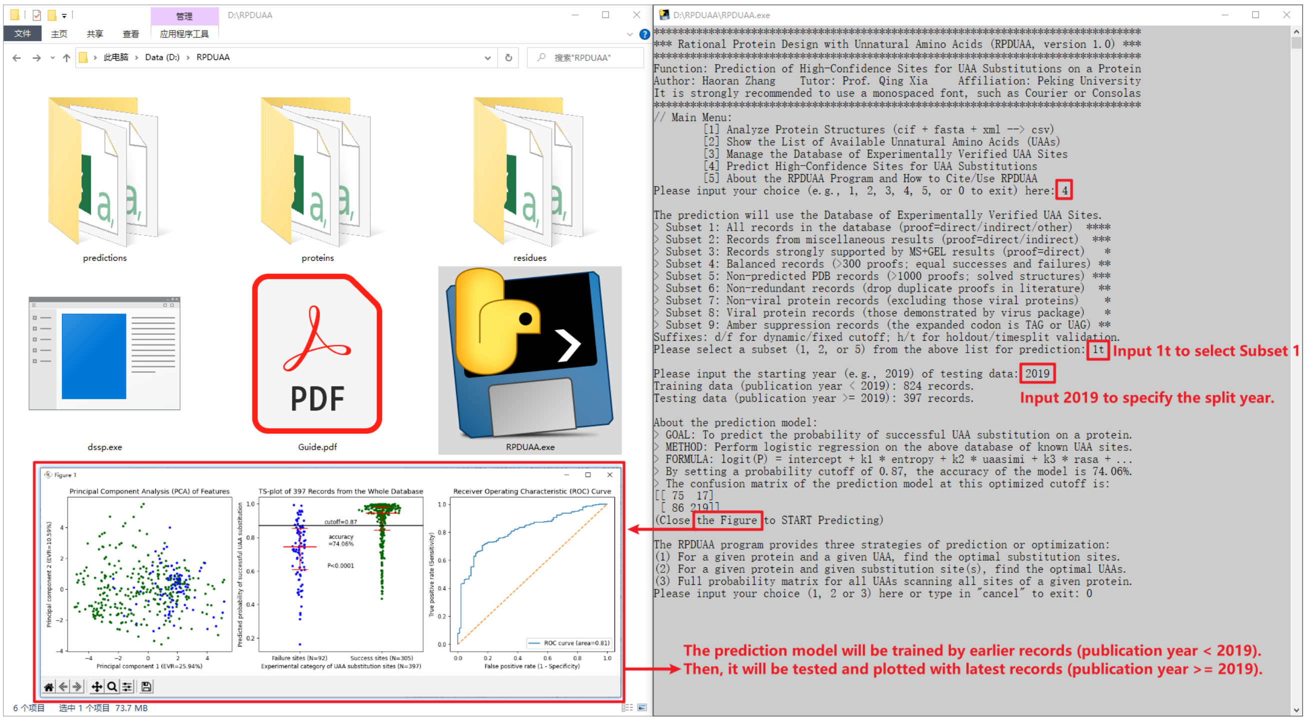

Suffix h for holdout validation of the prediction model

The RPDUAA program offers the function of holdout validation, which can be realized by inputting “1h”, “2h”, “5h” or likewise when selecting the subset. During the holdout validation, randomly selected 80% records from a subset are used to train the prediction model and optimize the parameters, and the rest 20% records are used to test the prediction model and plot the figures.

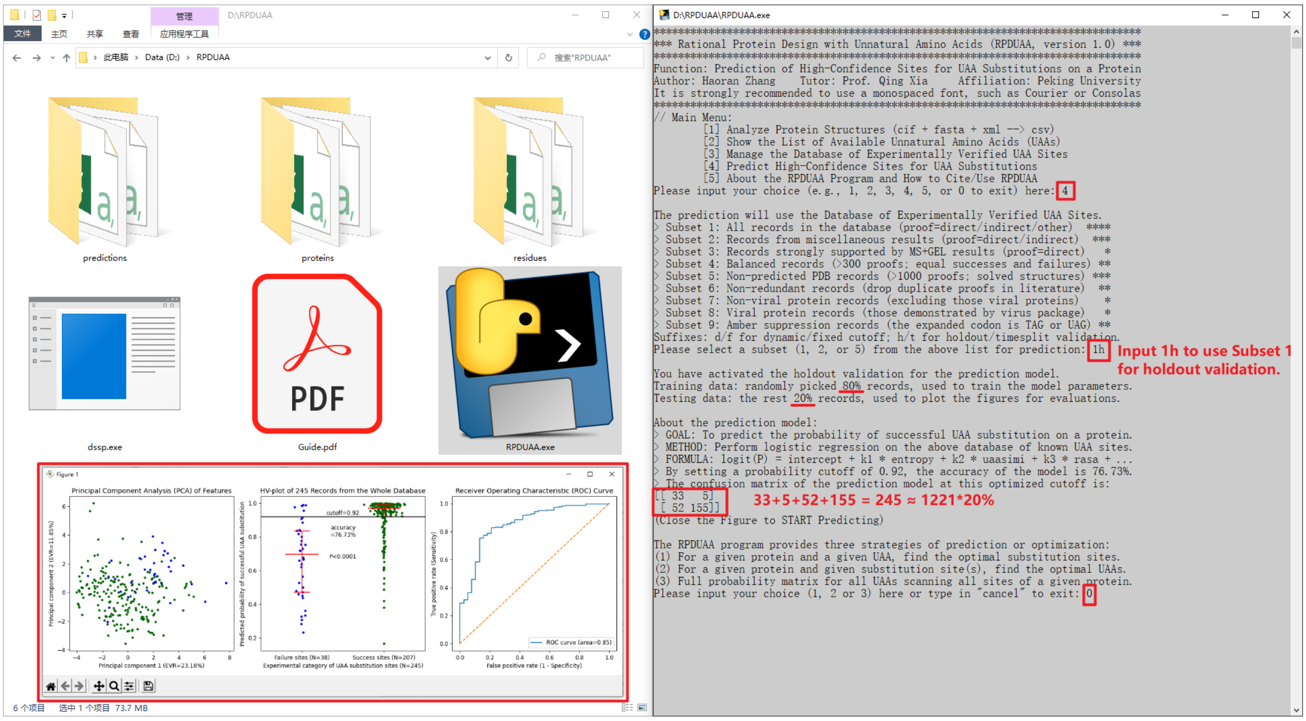

After choosing **Subset 1** with a suffix “h” (i.e., inputting “1h”), the model will be trained with 1221*80% ≈ 976 records. Then it will be tested with 1221*20% ≈ 245 = 33+5+52+155 records as the confusion matrix and reporting graph indicate. The ROC curve area of the prediction model using the 20% testing data is 0.85 in this run, which is fairly good. Since the 80% training data are randomly selected from the subset, the resulting parameters may vary in different runs. Users can perform a series of holdout validation. The holdout validation is a good method to study the performance of the prediction model.

Suffix x for 100 rounds of holdout/resampling validation

The RPDUAA program also supports performing 100 rounds of validations and exporting the key indexes. Just add a suffix “x” when selecting a subset.

By inputting “1x” when choosing a subset, you will perform 100 rounds of holdout validation using Subset 1 or the whole database. The key indexes such as optimal cutoff, accuracy, and ROC area of the 100 rounds will be reported as curves (see graphs next page) and exported into a csv file (hovdx100.csv in the “predictions” subfolder), which help you evaluate the stability of the model performance. This applies to other subsets (e.g., 2x, 5x, or 9x) except Subset 4. Typically, the optimal cutoff fluctuates around 0.84, the accuracy at optimal cutoff fluctuates around 80%, and the ROC area fluctuates around 0.85 for Subset 1.

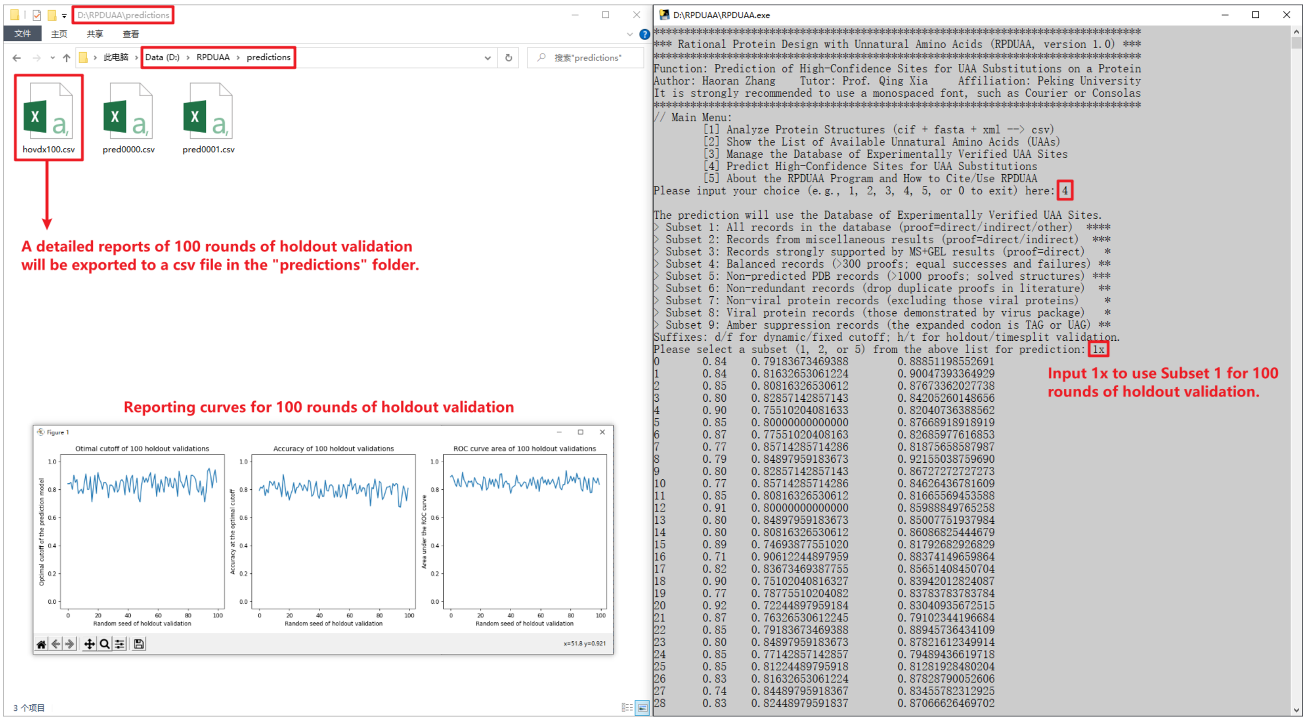

By inputting "4x" when choosing a subset, you will perform 100 rounds of resampling validation using Subset 4 or the balanced subset. All failure records are selected, but different success records (with equal number to failure records) are randomly resampled from the whole database to make Subset 4 for each round. The key indexes such as optimal cutoff, accuracy, and ROC area of the 100 rounds will be reported as curves (tested with all records in different Subset 4). Typically, the optimal cutoff fluctuates around 0.49, the accuracy at optimal cutoff fluctuates around 82%, and the ROC area fluctuates around 0.88 for Subset 4.

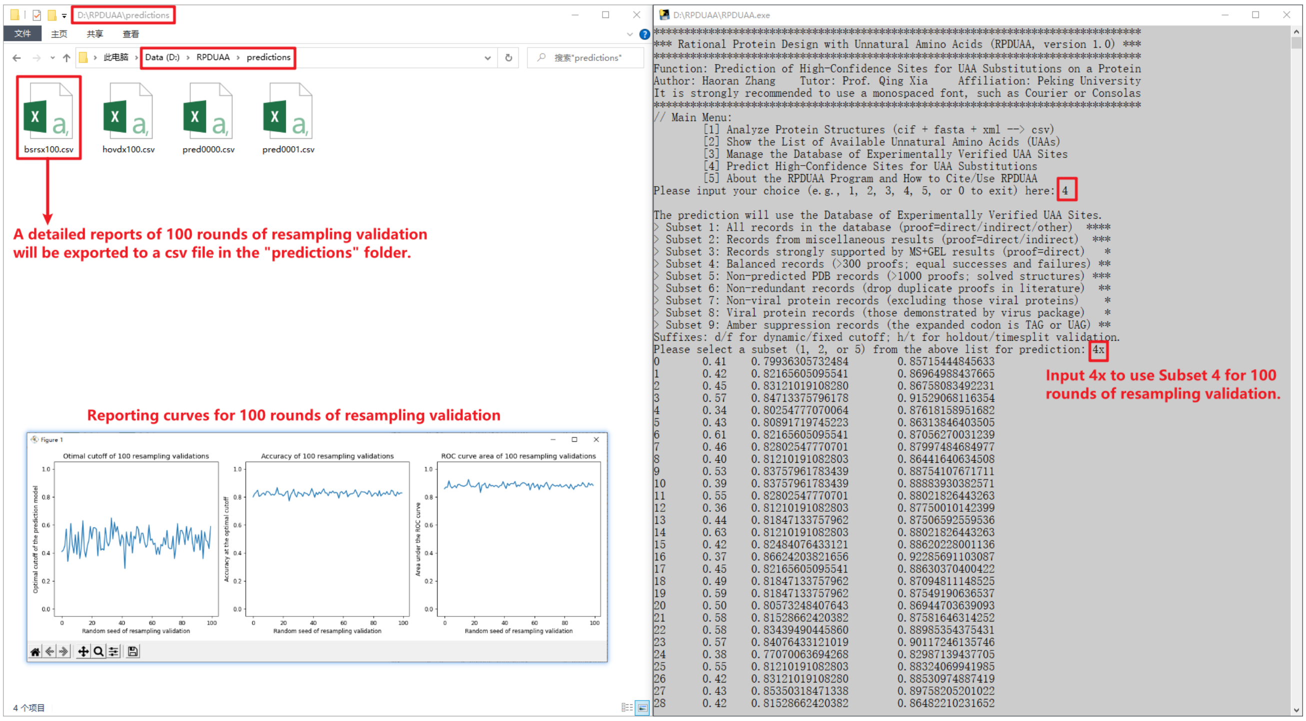

Chapter 5: Strategies of UAA Site Prediction with RPDUAA

The RPDUAA program provides three strategies of prediction or optimization:
(1) For a given protein and a given UAA, find the optimal substitution sites.
(2) For a given protein and given substitution site(s), find the optimal UAAs.
(3) Full probability matrix for all UAAs scanning all sites of a given protein.
In Chapter 1, we have showed how to use Strategy 1 to find the optimal substitution sites of NAEK (UAA) on the Herceptin protein (6mh2), and how to use Strategy 3 to get the full probability matrix for all UAAs on the Herceptin protein (6mh2). Here, we will predict the optimal UAAs for substituting either the K107 or K145 residue on the Herceptin protein (6mh2, chain A) using Strategy 2.

Prediction for user-defined sites across all UAAs (Strategy 2)

Double-click on “RPDUAA.exe” to start the program. Choose Task [4] in Main Menu. Use **Subset 1** as the train data for the prediction model. Close the emerging figure. In the prediction strategy step, select Strategy 2 by inputting “2” and pressing “Enter”. Then, the RPDUAA program will ask you to select a protein in the list. Input the PDB ID of the protein (6mh2), and select chain A in the next step.

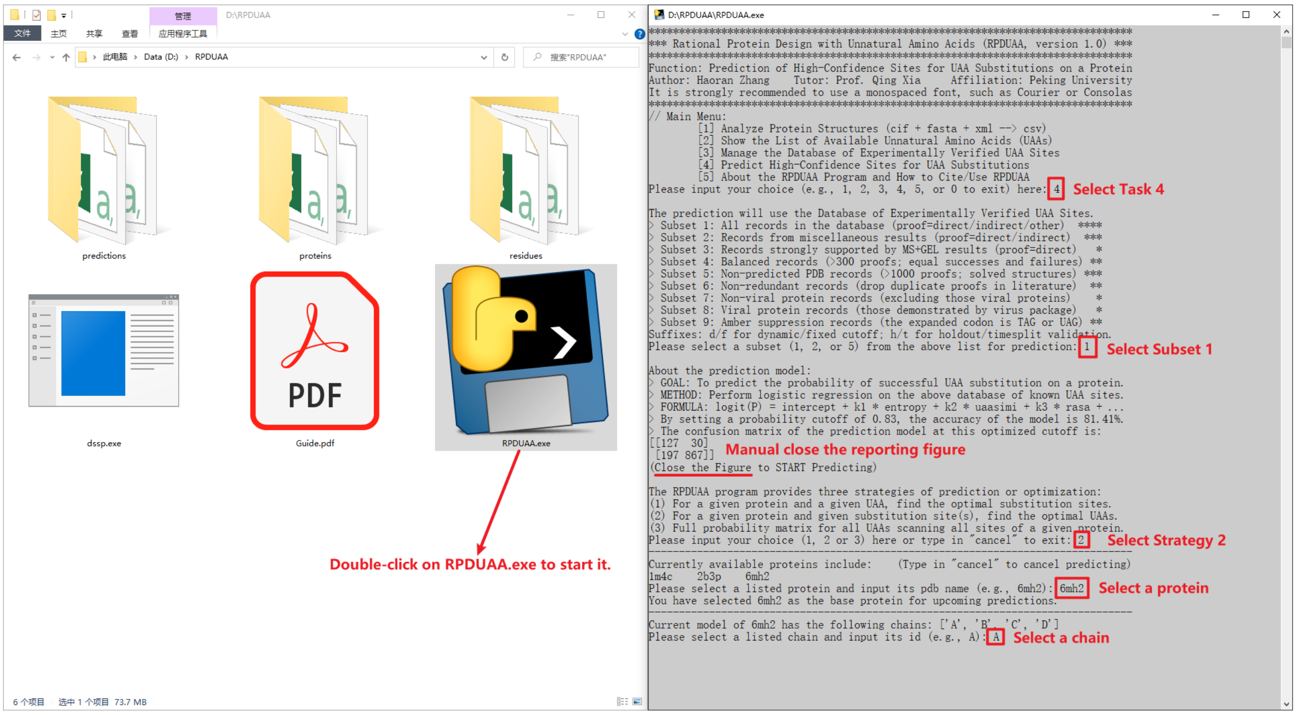

Then comes a list of available sites on chain A of 6mh2 (see the next-page figure). Input your candidate sites “K107 K145” separated by a space, and press “Enter”. If you have only one candidate site, just input it and there is no need of a space. The RPDUAA program will check whether your input candidate site(s) are in-list and ignore those out-of-list. Then the information (solvent exposure, conservation level, secondary structure, etc.) of those in-list sites will be called by RPDUAA from the previously analyzed “ready-to-use” csv file (namely, the “6mh2.csv” file in the “proteins” subfolder). The protein information will be used for predicting the probability of successful UAA substitution at these candidate sites. After setting the codon and proof level, the RPDUAA program will loop the whole available

UAA list to calculate the substitution probability. At this stage, the whole list of available UAAs in the RPDUAA program will be printed on the screen.

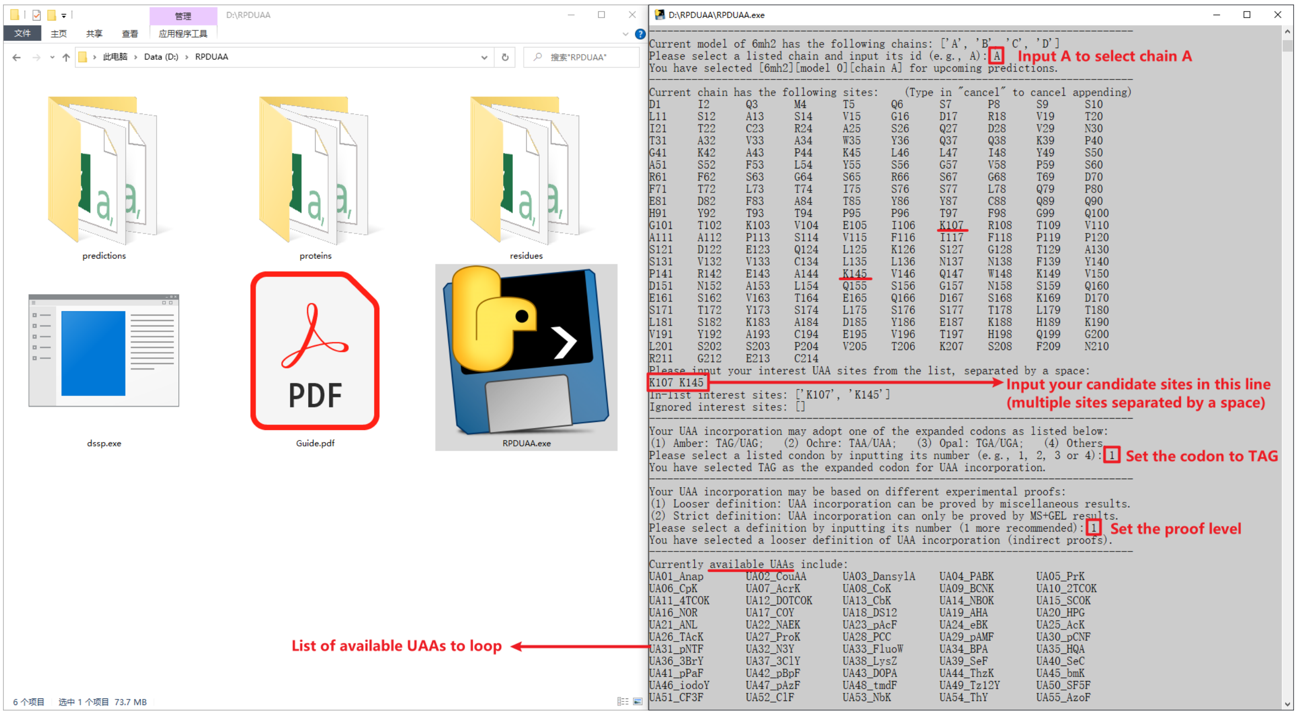

After a while, a brief report of the prediction will emerge under the available UAA list. Meanwhile, a csv file (pred000n.csv) will be created in the "predictions" subfolder, which contains the full details of the prediction. The last column of the csv file indicates the probability of successful UAA substitution, shown in a descending order. Strategy 2 reports the probability of all UAAs substituting your chosen sites on a protein (K107 and K145 on the 6mh2 protein here). User can refer to the csv file and pick the top-ranked UAAs with a probability higher than the optimal cutoff for further experimental studies.

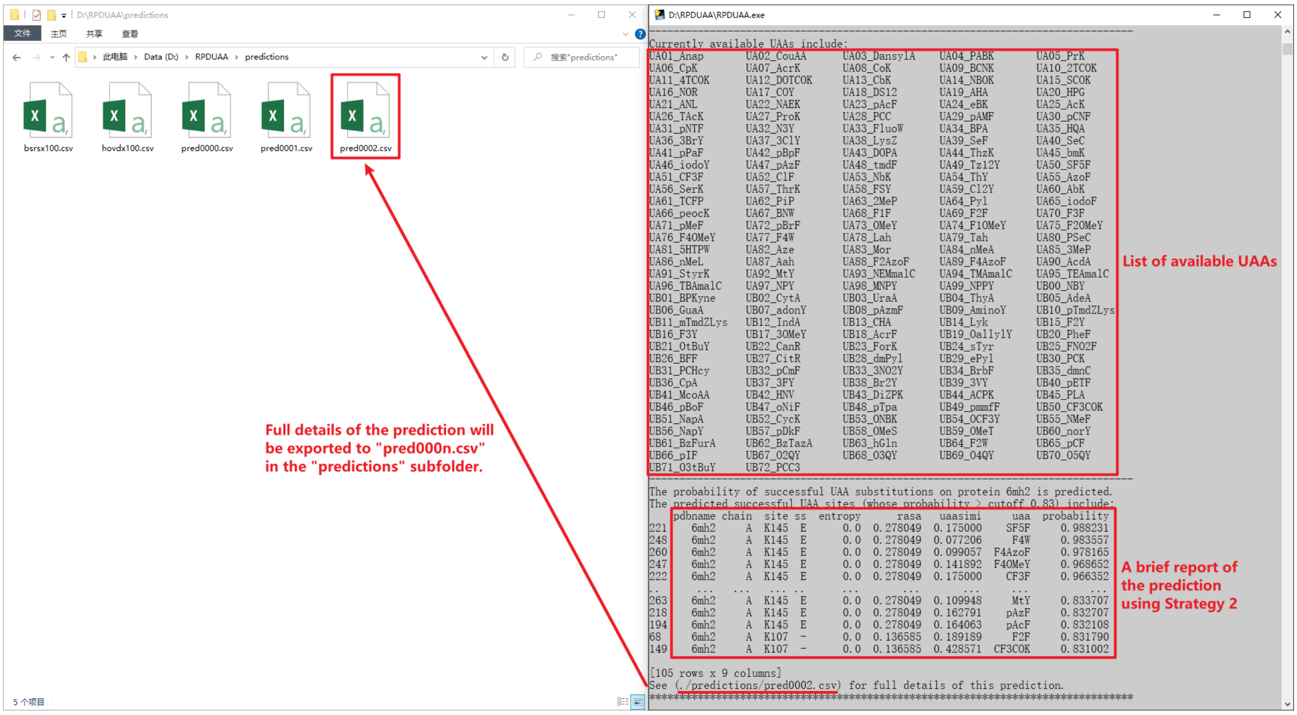

Prediction for all records in the database (Hidden p1e)

The Database of Experimentally Verified Database (known_uaa_sites.csv) is used to train and test the model. Perhaps you are interested in the predicted probability for each record in the database. The RPDUAA program realizes this goal by a hidden function, just by inputting “p1e” when selecting the subset in Task 4.

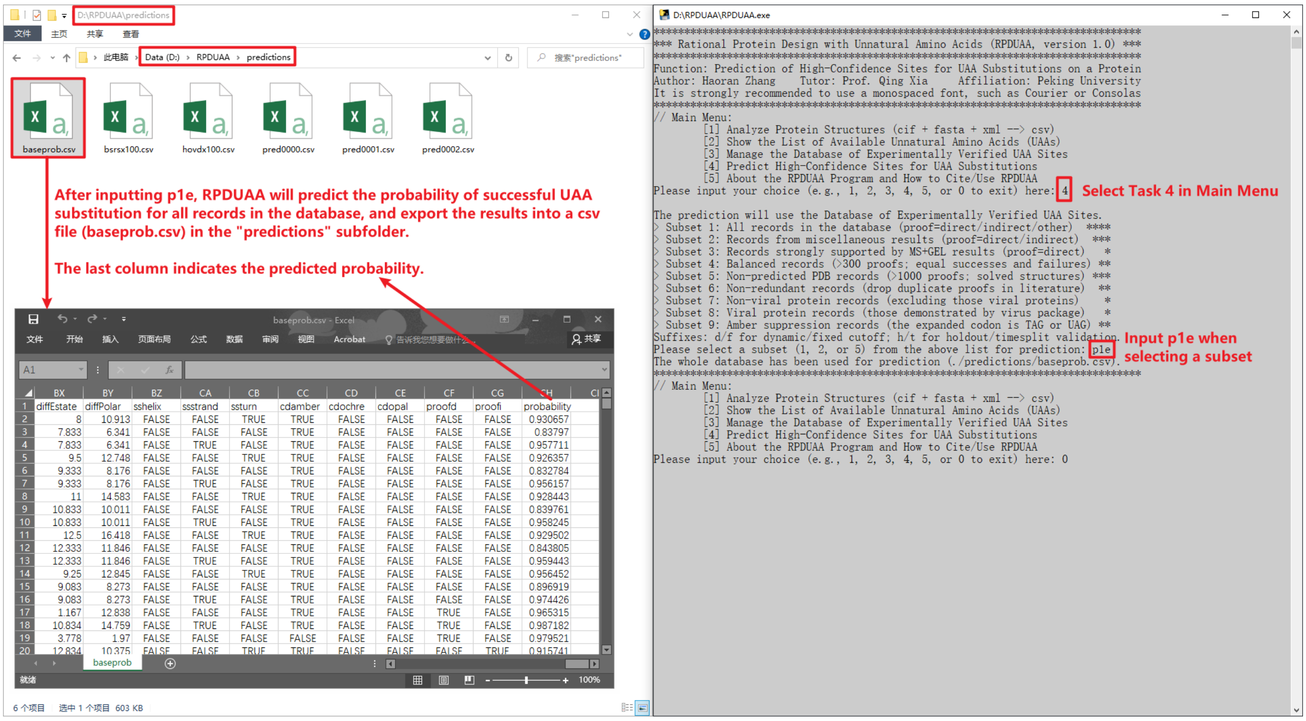

Prediction for unpublished laboratory records in the database (Hidden 4i)

You may have appended unpublished laboratory records to the database (see Chapter 3). Now you want to train the model with the published literature records, but test the model and plot the graphs with the unpublished laboratory records.

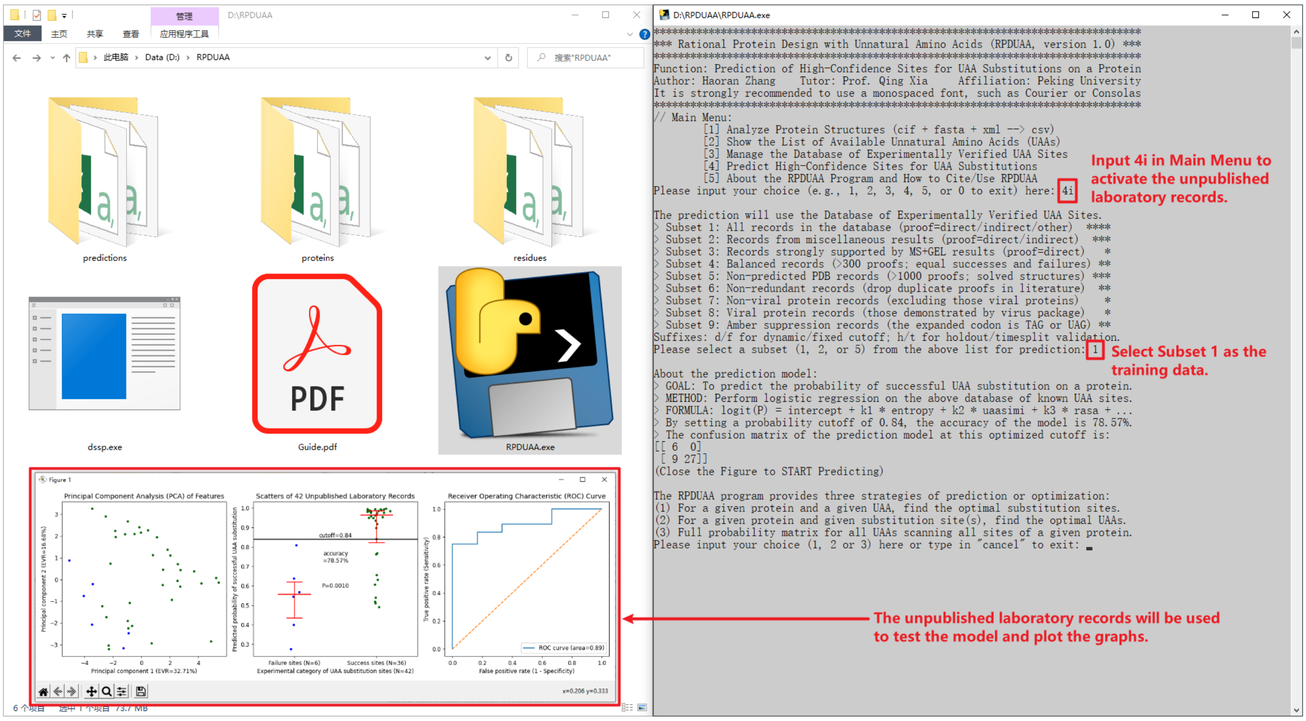

Note that the refdoi of those unpublished laboratory records in known_uaa_sites.csv should not start with 10, since the DOIs of published literature records always start with 10. The training and testing of prediction model described in previous chapters will exclude those unpublished laboratory records (42 unpublished records) and only use published literature records (1221 published records in total). Here, we can activate the unpublished laboratory records by inputting “4i” in Main Menu of the RPDUAA program, which is a hidden function. Then you need to choose a subset as the normal Task 4 requires. After choosing Subset 1 (or published records in the whole database), a 3-panel figure will emerge showing the performance on unpublished laboratory records (see last page).

Prediction based on logistic regression of absolute differences (Hidden 4a)

When preprocessing the differences between UAA and NAA on core molecular properties such as AlogP, the RPDUAA program will do direct subtraction (AloP_UAA - AlogP_NAA) as the default method. However, RPDUAA also supports another hidden preprocessing method that uses the absolute values (|AlogP_UAA – AlogP_NAA|). Just input “4a” in Main Menu of the RPDUAA program to activate the absolute differences.

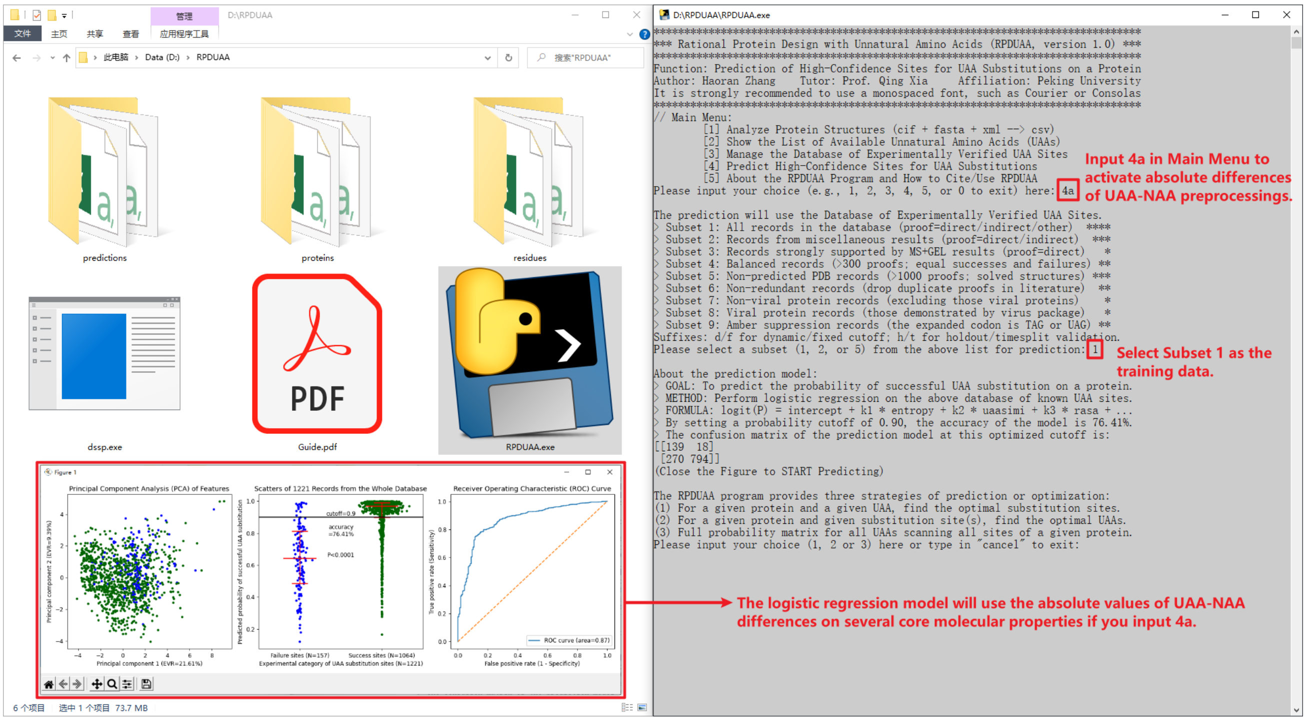

According to the tests by the author, performances of the two preprocessing methods are very close, although the default method (direct subtractions) gives slightly better performances than the hidden method (absolute differences) as shown below:

Database	Records	Direct subtractions			Absolute differences		
		Cutoff	Accuracy	ROC area	Cutoff	Accuracy	ROC area
Subset 1	1221	0.83	81.41%	0.87	0.90	76.41%	0.87
Subset 2	1100	0.86	79.55%	0.87	0.85	79.09%	0.87
Subset 3	625	0.98	68.37%	0.86	0.97	76.20%	0.84
Subset 4	314	0.53	83.76%	0.89	0.50	83.44%	0.89
Subset 5	1147	0.88	77.16%	0.87	0.87	77.94%	0.87

Database	Records	Direct subtractions			Absolute differences		
		Cutoff	Accuracy	ROC area	Cutoff	Accuracy	ROC area
Subset 6	1169	0.88	76.90%	0.86	0.89	75.88%	0.86
Subset 7	974	0.93	82.34%	0.81	0.96	67.97%	0.82
Subset 8	247	0.62	65.59%	0.70	0.68	61.64%	0.69
Subset 9	1154	0.88	76.34%	0.86	0.88	76.69%	0.86

The red font in the table indicates superior performances than the green font.

Prediction for the UAA incorporation efficiency (Hidden 4e)

Besides predicting the probability of successful UAA incorporation which is well-established, the RPDUAA program also has a hidden preliminary method for predicting the UAA incorporation efficiency by a generalized linear model. It is preliminary because the underlying database has only 124 records with their efficiencies based on exact protein yield, and urges more proofs to demonstrate the model performance. You can activate the prediction for the UAA incorporation efficiency by inputting "4e" in Main Menu of RPDUAA, a graph showing the performance of the generalized linear model (R=0.6367) will emerge, and the following prediction steps will be the same as described in Chapter 1. The only difference is that the final csv file (pred000n.csv in the "predictions" folder) will include a column of predicted UAA incorporation efficiency besides the predicted UAA incorporation probability.

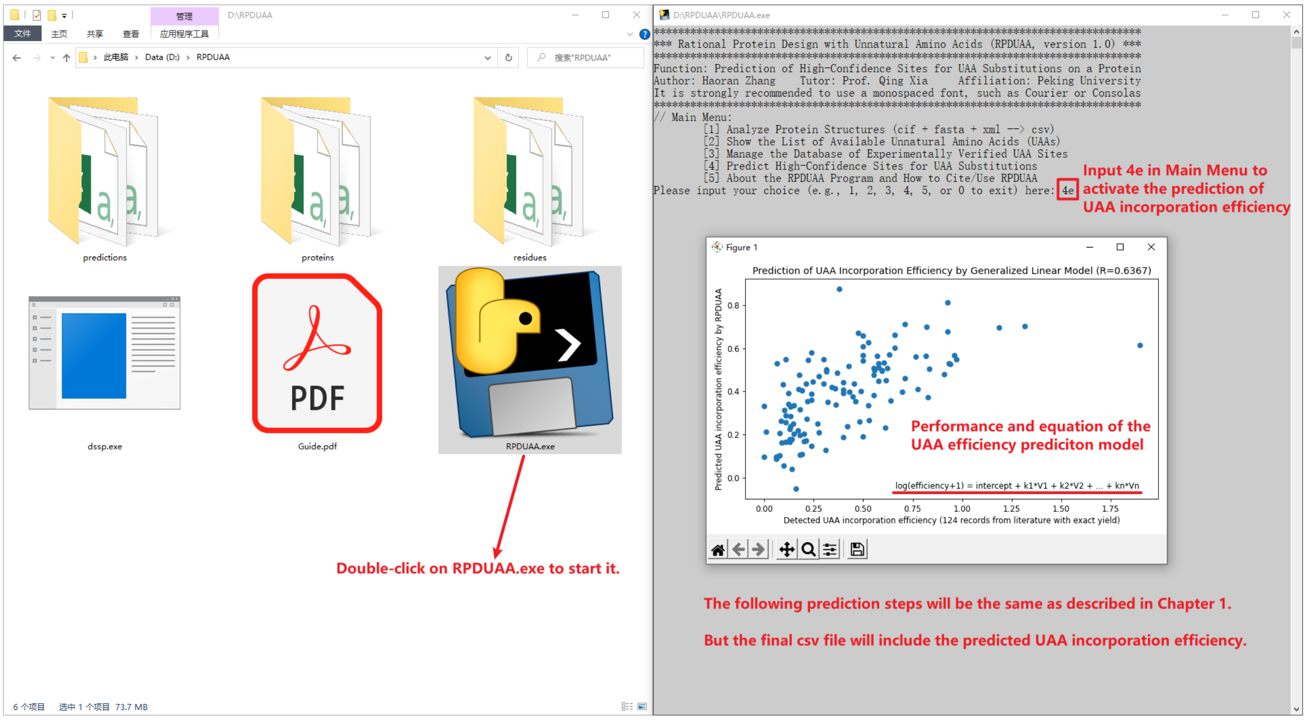

In summary, the RPDUAA program offers reliable and versatile predicting functions for the rational design of proteins incorporated with unnatural amino acids. You can use: Strategy 1 (fix UAA and scan sites), Strategy 2 (fix sites and scan UAAs) or Strategy 3 (full matrix for all combinations of UAAs and sites). Several hidden functions make RPDUAA powerful, such as 4a (absolute differences), 4i (unpublished in-laboratory data), 4e (predict efficiency) in Main Menu, and p1e in Task 4.

Chapter 6: Scope of Application and Limitation of RPDUAA

Current version (1.0) of the RPDUAA program is developed for the general case of UAA substitutions on proteins, and its application scope is shown below:

- The UAA should usually be **L-UAAs** like the natural amino acids. D-UAAs are not considered, for they may change the protein chain direction after substitution. Mixing L-UAAs and D-UAAs will decrease the creditability of prediction.
- The proteins can be **experimentally confirmed structures** from the Protein Data Bank (PDB), or **in silico predicted structures** by AlphaFold or RoseTTAFold, etc. The experimental structures are more convincing and recommended.
- The protein structure can be in **cif or pdb** format. The cif format (or PDBx/mmCIF) is strongly recommended. The pdb format is furtively supported by RPDUAA.
- For some PDB proteins, their cif or pdb files of may **contain RNA or DNA chains**. The RPDUAA program will automatically ignore these RNA or DNA chains, and only use the rest peptide chains for analysis. However, you need to **manually delete the corresponding RNA or DNA sequences in the fasta file** before performing a protein BLAST, because mixing peptide and nucleotide sequences in a fasta file can cause trouble to the protein BLAST.
- The proteins are allowed to contain **unique residues** besides the 20 canonical residues. But these unique residues will be screened off by RPDUAA.
- The **residue/site index** normally adopts the residue number in the fasta sequence, instead of the residue id or resseq in the protein structure (cif or pdb files). For example, site G128 of chain A refers to the 128th residue (glycine) in the fasta sequence of chain A, but it might have a different resseq in its protein structure. The csv report of protein analysis by RPDUAA provides both the site index and resseq. Remember **using the site index to look up in the fasta sequence**, and **using the resseq to find the correct residue in the protein structure**.
- During protein analysis, some abnormal lines may be generated in the csv report and a WARNING message will emerge indicating the abnormal line numbers. These abnormal lines are caused by the HSEB or DSSP’s failure to extract information for some start or end residues in a peptide segment. You can ignore it.

As an example, we will analyze the crystal structure of CIRV p19 bound to siRNA (PDB ID: 1rpu). The 1rpu structure contains both protein and RNA, as shown below:

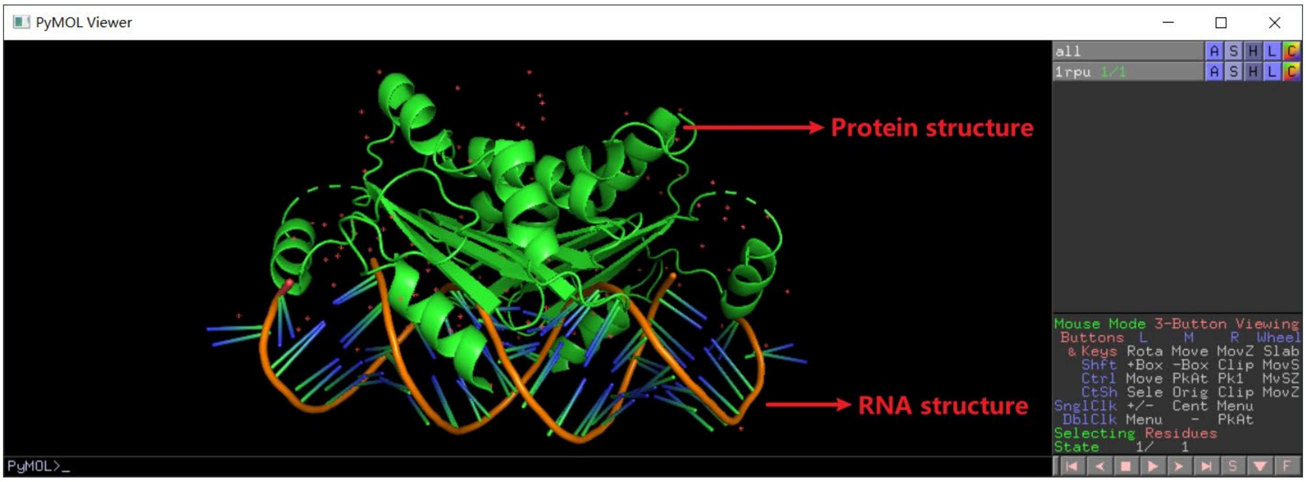

Now we want to analyze the 1rpu protein using the previously described procedure (see Chapter 1). After downloading the cif and fasta files of 1rpu from PDB, we can see mixing nucleotide and peptide sequences in the fasta file of 1rpu. We need to delete the nucleotide sequences manually and save the fasta file.

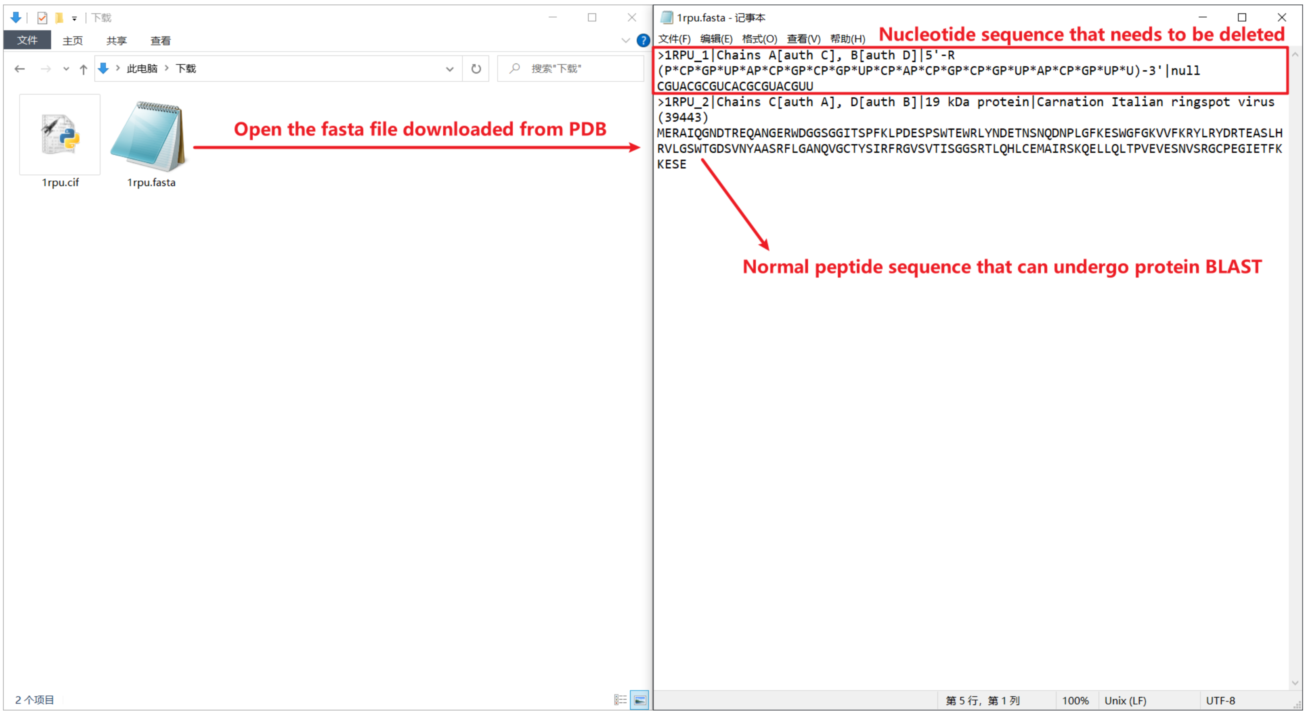

After deleting the nucleotide sequence in the fasta file of 1rpu, we can perform a protein BLAST on NCBI and save the results into a xml file. Then the cif, fasta and xml files can be renamed, placed into the "proteins" subfolder, and analyzed by RPDUAA as previously described in Chapter 1. After analyzing 1rpu, a WARNING message will emerge indicating two abnormal lines. You can check or ignore it.

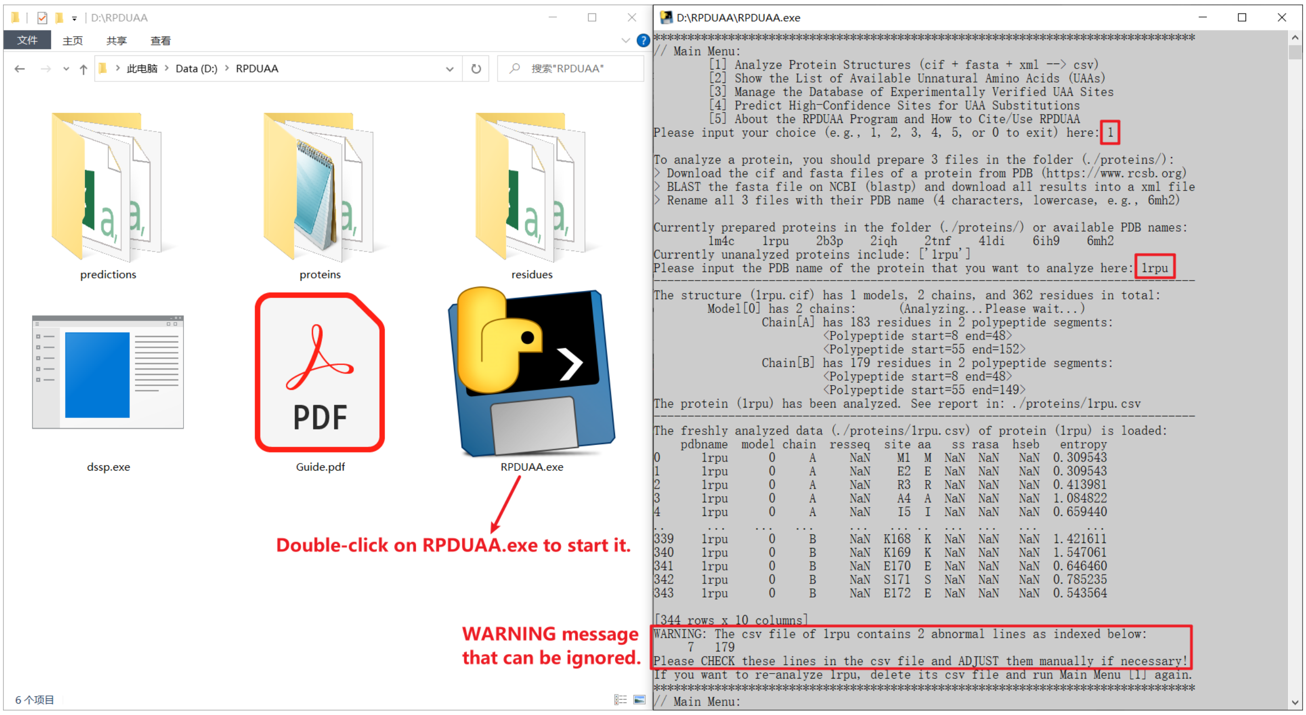

Chapter 7: Updates and New Version Maintenance of RPDUAA

The author welcomes user enquiries, suggestions, contributions or improvements of any kind on the RPDUAA program. Users can contact the author email, and the author will answer to his best. The author email (henryzhang@hsc.pku.edu.cn) can be found in the RPDUAA program.

Double-click on "RPDUAA.exe" to start the program. In Main Menu of RPDUAA, choose Task [5] by inputting "5" and pressing "Enter". The email of the author and other contributors will be shown along with other information of the RPDUAA program.

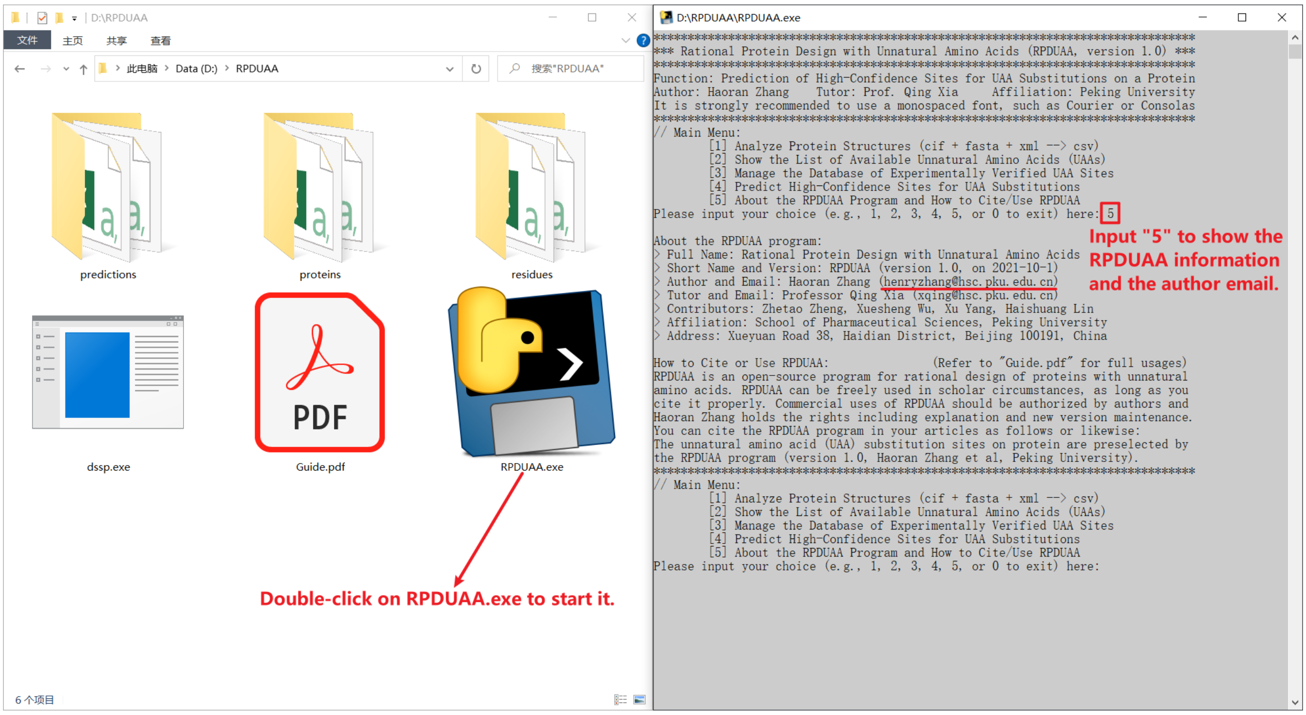

How to Cite RPDUAA:

RPDUAA is an open-source program distributed under the BSD license. RPDUAA can be freely used in scholar circumstances, as long as you cite it properly. Commercial uses of RPDUAA should inform the author in order to be authorized and Haoran Zhang reserves the rights including explanation and new version maintenance.

You can cite the RPDUAA program in your articles as follows or likewise:

The unnatural amino acid (UAA) substitution sites on protein are preselected by the RPDUAA program (version 1.0, Haoran Zhang et al, Peking University).

The author will continue updating the RPDUAA program and its related databases of UAA formulae and substitution sites. After substantial changes, new versions will be released. The current version is RPDUAA 1.0, finished on 2021-10-1. Users could contact the author email (henryzhang@hsc.pku.edu.cn) for new versions or databases. Finally, a paper that describes the functions and usages of the RPDUAA program is on the way. After publishing it on a peer-review journal, the RPDUAA program will be made open-source. We hope the RPDUAA program can benefit researchers in terms of protein engineering by rational substitutions with unnatural amino acids.

Written by Haoran Zhang (Henry Zhang)

Guidebook last modified on 2022-8-28
